# Michellamines A_6_ and A_7_, and further mono- and dimeric naphthylisoquinoline alkaloids from a Congolese *Ancistrocladus* liana and their antiausterity activities against pancreatic cancer cells†

**DOI:** 10.1039/c8ra00363g

**Published:** 2018-01-31

**Authors:** Blaise Kimbadi Lombe, Doris Feineis, Virima Mudogo, Reto Brun, Suresh Awale, Gerhard Bringmann

**Affiliations:** Institute of Organic Chemistry, University of Würzburg Am Hubland D-97074 Würzburg Germany bringman@chemie.uni-wuerzburg.de +49 931 31 84755; Faculté des Sciences, Université de Kinshasa B.P. 202 Kinshasa XI Democratic Republic of the Congo; Swiss Tropical and Public Health Institute Socinstrasse 57 CH-4002 Basel Switzerland; University of Basel Petersplatz 1 CH-4003 Basel Switzerland; Division of Natural Drug Discovery, Institute of Natural Medicine, University of Toyoma 2630 Sugitani Toyama 930-0194 Japan suresh@inm.u-toyama.ac.jp

## Abstract

Michellamines A_6_ (1) and A_7_ (2) are the first dimers of 5,8′-coupled naphthylisoquinoline alkaloids with *cis*-configured stereocenters in both tetrahydroisoquinoline subunits. They were isolated from the leaves of a recently discovered, yet unidentified Congolese *Ancistrocladus* liana that shares some morphological characteristics with *Ancistrocladus likoko*. Two further new dimeric analogs, michellamines B_4_ (3) and B_5_ (4), were obtained, along with two previously likewise unknown monomers, ancistrobonsolines A_1_ (5) and A_2_ (6), which, besides one single known other example, are the only naphthyldihydroisoquinolines with an *M*-configured biaryl axis and *R*-configuration at C-3. Moreover, five compounds earlier reported from other *Ancistrocladus* species were identified, ancistroealaine C (7), korupensamines A (8a) and B (8b), and michellamines A_2_ (9) and E (10). Their complete structural elucidation succeeded due to the fruitful interplay of spectroscopic, chemical, and chiroptical methods. Chemotaxonomically, the stereostructures of the metabolites clearly delineate this Congolese *Ancistrocladus* liana from all known related species, showing that it might be a new taxon. Ancistrobonsolines A_1_ (5) and A_2_ (6) exhibited strong preferential cytotoxicities against human PANC-1 pancreatic cancer cells under nutrient-deprived conditions, without displaying toxicity in normal, nutrient-rich medium. Against cervical HeLa cancer cells, the dimeric alkaloids michellamines A_6_ (1) and E (10) displayed the highest cytotoxic activities, comparable to that of the standard agent, 5-fluorouracil. Furthermore, ancistrobonsolines A_1_ (5) and A_2_ (6) showed weak-to-moderate antiprotozoal activities.

## Abstract 2

The first dimers of 5,8′-coupled naphthylisoquinolines with two 1,3-*cis*-configured tetrahydroisoquinoline portions and their cytotoxicities against cancer cells are described.

## Graphical abstract

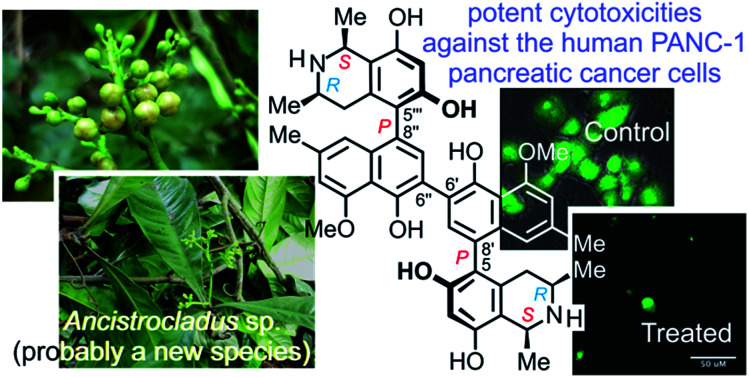

## Introduction

Plants belonging to the genus *Ancistrocladus* Wall. (Ancistrocladaceae) continue to receive much attention, mainly because they produce a class of structurally, biosynthetically, and pharmacologically unusual compounds, the naphthylisoquinoline alkaloids.^1^ The genus *Ancistrocladus* comprises 18 botanically accepted species of tropical lianas,^2–4^ of which four occur in the Democratic Republic of the Congo (DRC), namely, *A. ealaensis* J. Léonard,^5^*A. congolensis* J. Léonard,^5^*A. likoko* J. Léonard,^5^ and *A. ileboensis* G. Heubl, V. Mudogo & G. Bringmann.^4^ The number of Congolese *Ancistrocladus* species may, however, be actually much higher, as there have not yet been thorough and specific botanical campaigns covering the entire Congolese forest, which is so vast and so rich in biodiversity. This assumption is also supported by recent phylogenetic investigations on *Ancistrocladus* samples collected at different sites in DRC,^6^ which hinted at the presence of a potentially new taxon from the region near the village Bonsolerive, close to the town Mbandaka, in the northwestern part of DRC.

Most recently, likewise close to the village Bonsolerive, we have discovered another *Ancistrocladus* liana. Particularly striking were its leaves, which were much larger (about 57 cm long and 12 cm wide) than those of any other *Ancistrocladus* plant species found nearby. This liana had hooked inflorescences, which, together with the aforementioned large size of the leaves, were reminiscent of *A. likoko*, the only known Congolese species with similar – yet different – morphological characteristics.^2,5^ Comparative LC-UV-MS profiling of the leaf alkaloid pattern of this liana and that of an authentic sample of *A. likoko*, however, revealed substantial phytochemical differences between the two samples. This metabolic divergence and the fact that *A. likoko* had so far not been found to occur in that sampling area indicated that the liana might belong to a new, as yet undescribed plant species or subspecies. The isolation and structural elucidation of its alkaloids thus became a rewarding task, not only for the chemotaxonomic characterization of this liana and its possible delineation from other *Ancistrocladus* species, but also for their biological evaluation.

**Fig. 1:**
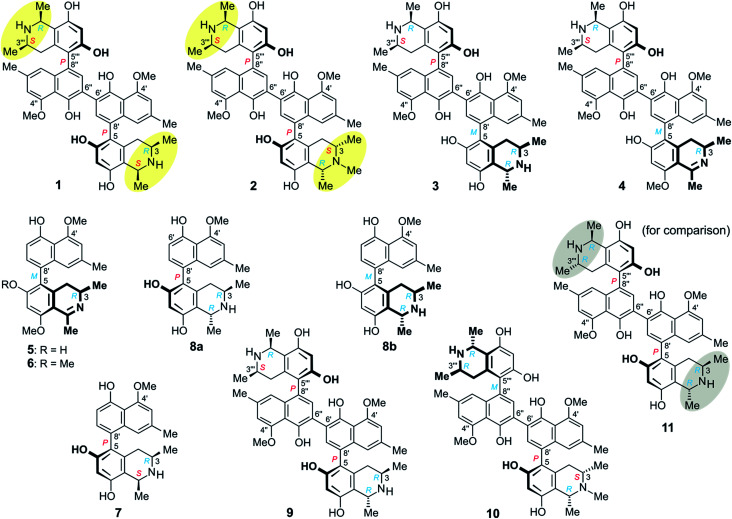
Metabolites isolated from an as yet unidentified Congolese *Ancistrocladus* liana, among them six new compounds: michellamines A_6_ (1), A_7_ (2), B_4_ (3), and B_5_ (4), ancistrobonsolines A_1_ (5) and A_2_ (6), and five previously known ones: ancistroealaine C (7), korupensamines A (8a) and B (8b), and michellamines A_2_ (9) and E (10). Yellow ellipses on 1 and 2 highlight the combination of two 1,3-*cis*-configurations, never observed in any other related dimer; for reason of comparison, see the structure of the well-known michellamine A (11) with its 1,3-*trans*-configurations (underlaid in gray) in both molecular halves – this compound is not produced by the investigated Congolese liana.

Herein we report on the isolation and structural elucidation of eleven mono- and dimeric naphthylisoquinoline alkaloids from the leaves of this yet unidentified *Ancistrocladus* liana (Fig. 1). The isolated metabolites comprise six previously unknown compounds: michellamines A_6_ (1), A_7_ (2), B_4_ (3), and B_5_ (4), ancistrobonsoline A_1_ (5) and its 6-*O*-methyl derivative, ancistrobonsoline A_2_ (6), and five analogs described from earlier work on other *Ancistrocladus* plants: ancistroealaine C (7),^7^ korupensamines A (8a) and B (8b),^8^ and michellamines A_2_ (9)^9^ and E (10).^10^ Furthermore, we discuss the chemotaxonomic position of this liana relative to other *Ancistrocladus* species. The cytotoxic activities of the isolated compounds against cervical HeLa cancer cells and their antiausterity potencies against PANC-1 human pancreatic cancer cells are also described, as well as the antiprotozoal properties of the new monomeric compounds.

## Results and discussion

### Isolation and structural elucidation of metabolites

#### Identification of the known compounds 7–10

**Fig. 2:**
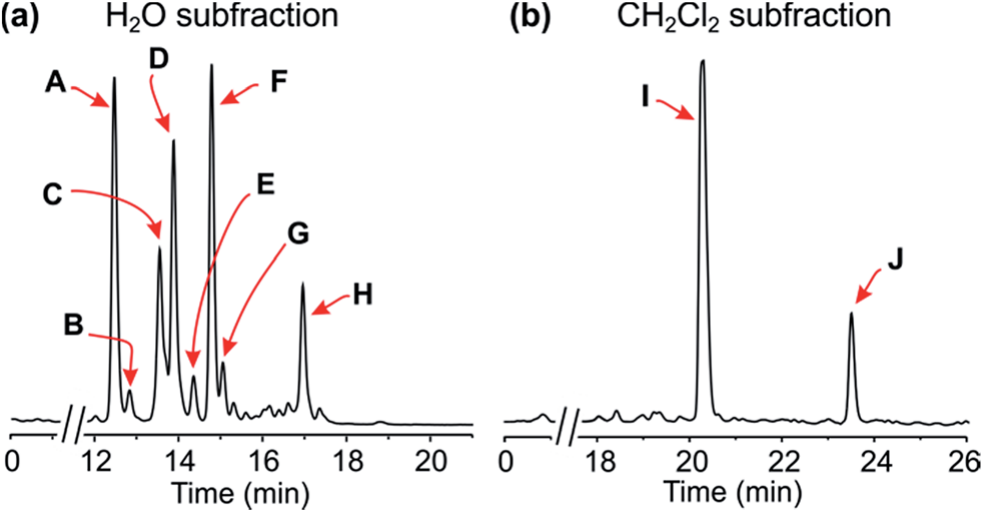
HPLC profiles of the alkaloid-enriched subfractions from the leaves of the plant.

Extraction of the air-dried leaves with a mixture of methanol and dichloromethane (1 : 1), followed by concentration under reduced pressure, provided a crude residue, which was then submitted to a cation-exchange column to remove undesired, non-basic metabolites. The resulting alkaloid-enriched fraction was partitioned between water and dichloromethane, and monitored by HPLC (Fig. 2).

Resolution of the water-phase subfraction on a preparative HPLC column permitted isolation of nine pure constituents, obtained as amorphous solids. The fastest-eluting compound (Peak A, Fig. 2a) was found to be ancistroealaine C (7), earlier known from *Ancistrocladus ealaensis*.^7^ The second alkaloid (Peak B) was korupensamine A (8a), while peak C contained two alkaloids overlapping each other, korupensamine B (8b) and michellamine A_2_ (9), and peak G was michellamine E (10). These four metabolites (8a/b–10) had previously been isolated from other *Ancistrocladus* species, yet only from Central Africa: The korupensamines A (8a) and B (8b), first known from the Cameroonian plant *A. korupensis*^8^ as well as michellamine E (10),^10^ had both also been reported from *A. likoko*,^11,12^ where they occur as major metabolites. Korupensamine A (8a) is likewise the main naphthylisoquinoline alkaloid in *A. congolensis*,^9,13^ in which michellamine A_2_ (9) was discovered^9^ before its more recent detection as a minor constituent of *A. likoko*.^12^ The other four remaining compounds from the aqueous subfraction (Peaks D, E, F, and H, Fig. 2a), as well as those from the dichloromethane subfraction (Peaks I and J, Fig. 2b), were as yet unknown.

#### Michellamine A_6_ (1)

The first new metabolite, compound 1 (Peak D, Fig. 2a), had a molecular formula C_46_H_48_N_2_O_8_, as evidenced from HRESIMS (757.3486, [M + H]^+^). ^1^H and ^13^C NMR measurements showed only a half set of signals, which indicated that this metabolite consisted of two equivalent molecular portions, but left open the question of whether the two halves were homomorphous or enantiomorphous to each other. The latter possibility (*i.e.*, the presence of an achiral *meso* compound) was excluded by its optical activity, showing that 1 was *C*_2_-symmetric.

**Fig. 3:**
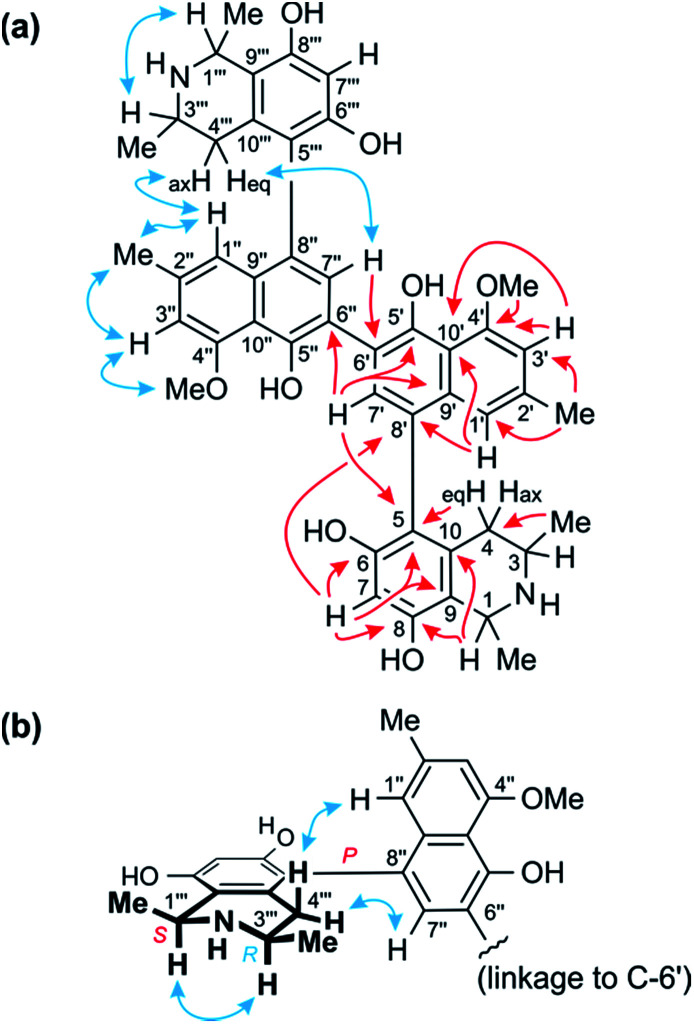
(a) Key HMBC (red single arrows) and ROESY (blue double arrows) interactions evidencing the constitution of the homodimer 1. (b) ROESY interactions indicating the relative and – given the results of oxidative degradation – absolute configurations of the two identical portions (=ancistroealaine C, 7) of 1.

The ^1^H NMR spectrum (Table 1) exhibited four aromatic protons. Two of them displayed a *meta*-coupling, typical of protons at C-1′ and C-3′,^7–10^ while the other two appeared as singlets, which suggested the presence of a symmetric dimer of 5,6′-, 5,8′-, 7,6′-, or 7,8′-coupled naphthylisoquinoline monomers. The aliphatic region, with two three-proton doublets (Me-1 and Me-3), signals for two aryl substituents (a methyl group and a methoxy function), a quartet, a multiplet, and two doublet of doublets, was, however, closely similar to that of the likewise isolated known ancistroealaine C (7), so that a 5,8′-coupled molecular framework was expected. In agreement with this assumption, one of the two aromatic singlets was assigned as H-7, from its HMBC correlations with C-6, C-8, and C-9 (Fig. 3a). This showed that the other, remaining singlet had to be at C-7′ and, hence, clearly excluded any biaryl linkage involving C-7.

**Table 1:** ^1^H and ^13^C NMR data of michellamine A_6_ (1), and of ancistrobonsolines A_1_ (5) and A_2_ (6)^a^

Position	1^b^		5		6	
	*δ*_H_	*δ*_C_	*δ*_H_	*δ*_C_	*δ*_H_	*δ*_C_
1	4.64, q (6.5)	52.4		175.8		176.3
3	3.27, m	51.0	3.70, m	49.5	3.71, m	49.6
4_eq._	2.64, dd (3.3, 17.3)	33.3	2.41, dd (5.8, 16.9)	33.6	2.42, dd (5.8, 16.9)	33.4
4_ax_	2.27, dd (12.0, 17.3)	33.3	2.48, dd (11.3, 16.9)	33.6	2.49, dd (11.3, 16.9)	33.4
5		119.4		122.5		123.6
6		156.8		167.8		168.7
7	6.47, s	102.9	6.67, s	99.3	6.83, s	95.9
8		156.5		165.8		166.4
9		113.0		108.8		109.2
10		135.2		142.9		141.6
1′	6.84, pt (0.9)	119.3	6.72, pt (1.1)	118.7	6.64, pt (1.1)	118.6
2′		137.7		138.2		138.2
3′	6.86, pd (1.3)	108.1	6.83, pd (0.9)	107.9	6.82, pd (0.9)	107.8
4′		158.2		158.2		158.1
5′		152.4		156.4		156.2
6′		120.4	6.80, d (7.9)	110.4	6.78, d (7.9)	110.3
7′	7.29, s	134.9	7.05, d (7.9)	131.7	7.00, d (7.9)	131.4
8′		124.3		123.1		123.3
9′		137.1		136.7		136.6
10′		115.3		115.0		114.8
1-Me	1.82, d (6.5)	20.0	2.79, pd (1.5)	24.8	2.82, pd (1.5)	24.9
3-Me	1.26, d (6.5)	18.9	1.26, d (6.6)	18.2	1.27, d (6.6)	18.1
2′-Me	2.37, s	22.3	2.34, pd (0.6)	22.3	2.32, pd (0.8)	22.3
6-OMe					3.83, s	57.2
8-OMe			4.04, s	56.8	4.15, s	57.0
4′-OMe	4.10, s	57.1	4.10, s	57.0	4.10, s	57.0

a ^1^H and ^13^C NMR data were recorded in methanol-*d*_4_ (*δ* in ppm).

b These data are identical for the second molecular half of the *C*_2_-symmetric dimer 1.

The two remaining possible coupling types (5,6′ or 5,8′) were further discriminated by joint correlative HMBC signals from H-7 and H-1′ to C-8′ (Fig. 3a), which excluded a 5,6′-coupling, where such interactions would not have been observed. Thus, the naphthalene and isoquinoline portions were linked to each other by a 5,8′-axis, and the two monomeric naphthylisoquinoline halves were therefore connected *via* C-6′. These couplings, in particular the 6′,6′′-linkage between the naphthalene portions, were further corroborated by the chemical shifts of C-6′ (*δ*_C_ 120.4) and of the surrounding nuclei, C-5′ (*δ*_C_ 152.4) and C-7′ (*δ*_C_ 134.9), which were near-identical to those of other dimers with similar molecular frameworks (*e.g.*, michellamine A_2_ (9):^9^*δ*_C_ 120.3 for C-6′, 152.3 for C-5′, and 134.7 for C-7′). The location of the methoxy group at C-4′ was deduced from NOE interactions of its protons with H-3′, in conjunction with joint HMBC correlations from both the *O*-methyl protons and H-3′ to C-4′ (Fig. 3a).

Based on NOESY interactions of the proton at C-3 and the one at C-1 (Fig. 3a), the relative configuration at the stereocenters C-1 and C-3 was determined to be *cis*. Ruthenium-mediated oxidative degradation^14^ of this alkaloid led to the *R*-enantiomer of 3-aminobutyric acid (derived from C-3), as monitored by GC-MSD analysis of the Mosher derivatives. This revealed C-3 to be *R*-configured and, given the aforementioned relative *cis*-configuration, C-1 had to be *S*-configured.

At the naphthalene-isoquinoline biaryl axes, within the 5,8′-coupled monomeric halves, the stereo-array was determined by NOESY interactions of the proton at C-7′ with the equatorial proton at C-4 (*δ*_H_ = 2.64 ppm; *J* = 17.3, 3.3 Hz) and between the axial proton (*δ*_H_ = 2.27 ppm; *J* = 17.3, 12.0 Hz) and the aromatic proton at C-1′. This, in conjunction with the absolute *R*-configuration at C-3, as depicted in Fig. 3b, indicated *P*-configuration at the outer axes, revealing these portions to be structurally identical to the co-occurring known ancistroealaine C (7). Hence, the metabolite was found to be the 6′-homocoupling product of 7, with the absolute stereostructure 1 presented in Fig. 1. This was further confirmed by the similarity of the ECD (electronic circular dichroism) spectrum of 1 with that of the co-occurring, likewise *P*,*P*-configured michellamine A_2_ (9). Owing to this combination of two *P*-configured outer biaryl axes, the new compound was included in the ‘series A’ of michellamines – which so far comprised michellamines A–A_5_ – and was thus named michellamine A_6_. Its 1,3-*cis*-configurations in both tetrahydroisoquinoline portions, as highlighted in Fig. 1, is a unique structural feature that had not been found in any other similarly coupled naphthylisoquinoline dimers.

#### Michellamine A_7_ (2)

The second new alkaloid, compound 2, corresponding to peak E in Fig. 2a, had a molecular weight 14 units larger than that of 1, matching with the molecular formula C_47_H_50_N_2_O_8_ (*m*/*z* 771.3655, HRESIMS). Its UV spectrum, which was very similar to that of 1, indicated that 2 was a 6′,6′′-coupled dimer, too. This was further supported by its ^1^H NMR spectrum (Table 2), which closely resembled that of the co-isolated michellamine A_2_ (9), except for the presence of one additional three-proton singlet at 3.04 ppm. In the HSQC experiment, this extra methyl signal correlated with the carbon atom at 41.4 ppm, thus indicating 2 to be an *N*-methylated michellamine-type dimer.

**Table 2:** ^1^H and ^13^C NMR data of michellamines A_7_ (2), B_4_ (3), and B_5_ (4)^a^

Position	2		3		4	
	*δ*_H_	*δ*_C_	*δ*_H_	*δ*_C_	*δ*_H_	*δ*_C_
1/1′′′	4.64, q (6.6)/4.64, q (6.6)	62.6/52.5	4.74, q (6.7)/4.64, q (6.5)	49.5/52.4	—/4.64, q (6.6)	175.8/52.4
3/3′′′	3.23, m/3.29, m	60.6/51.0	3.66, m/3.29, m	45.4/50.9	3.72, m/3.29, m	49.4/50.9
4_eq._/4′′′_eq._	2.36^b^, dd/2.65, dd (4.3, 17.8)	34.5/33.4	2.37^b^, dd/2.61, dd (3.3, 17.4)	34.1/33.3	2.52, dd^c^/2.59, dd^c^	33.6/33.3
4_ax_/4′′′_ax_	2.69, dd (11.4; 18.0)/2.28, dd (12.2, 17.8)		2.53, dd (11.7; 18.1)/2.26, dd (12.0, 17.4)		2.61, dd^c^/2.25, dd (11.9, 17.2)	
5/5′′′		119.1/119.4		119.1/119.2		122.3/119.2
6/6′′′		157.0/156.8		157.0/156.8		167.8/156.8
7/7′′′	6.47, s/6.47, s	102.6/102.9	6.46, s/6.48, s	102.2/102.9	6.69, s/6.48, s	99.4/102.9
8/8′′′		155.7/156.5		155.7/156.5		165.8/156.5
9/9′′′		113.4/113.0		113.4/113.0		108.8/113.0
10/10′′′		135.2/135.2		133.2/135.2		143.0/135.2
1′/1′′	6.78, s/6.85, s	119.5/119.4	6.84, s/6.84, s	119.4/119.3	6.77, pt (1.2)/6.84, pt (1.2)	118.9/119.4
2′/2′′		137.6/137.7		137.8/137.8		138.2/137.8
3′/3′′	6.87, s/6.86, s	108.2/108.1	6.87, s/6.87, s	108.3/108.1	6.88, d (1.2)/6.86, d (1.4)	108.3/108.1
4′/4′′′		158.3/158.2		158.2/158.2		158.2/158.3
5′/5′′		152.5/152.4		152.4/152.4		152.5/152.9
6′/6′′		120.4/120.4		120.3/120.3		120.0/120.4
7′/7′′	7.33, s/7.29, s	135.7/135.0	7.27, s/7.30, s	135.3/134.9	7.30, s/7.30, s	135.2/134.9
8′/8′′		124.0/124.3		124.3/124.3		122.6/124.3
9′/9′′		136.5/137.1		136.6/137.2		136.1/137.2
10′/10′′		115.2/115.3		115.4/115.3		115.2/115.3
1-Me/1′′′-Me	1.77, d (6.7)/1.82, d (6.6)	19.6/20.0	1.70, d (6.7)/1.82, d (6.5)	18.5/20.0	2.77, d (1.4)/1.82, d (6.6)	24.9/20.0
3-Me/3′′′-Me	1.33, d (6.6)/1.27, d (6.5)	18.2/18.9	1.28, d (6.3)/1.24, d (6.4)	19.4/18.9	1.28, d (6.7)/1.24, d (6.5)	18.2/18.9
2′-Me/2′′-Me	2.36, s/2.37, s	22.3/22.3	2.37, s/2.37, s	22.3/22.3	2.37, s/2.37, s	22.3/22.3
8-OMe					4.03, s	56.8
4′-OMe/4′′-OMe	4.12, s/4.10, s	57.1/57.1	4.11, s/4.11, s	57.2/57.2	4.11, s/4.10, s	57.1/57.2
*N*-Me	3.04, s	41.4				

a The NMR data were recorded in methanol-*d*_4_ (*δ* in ppm).

b This signal was overlapped by that of 2′-Me, but could be deduced to be dd, from the respective 2D NMR cross peaks.

c These signals overlapped each other.

**Fig. 4:**
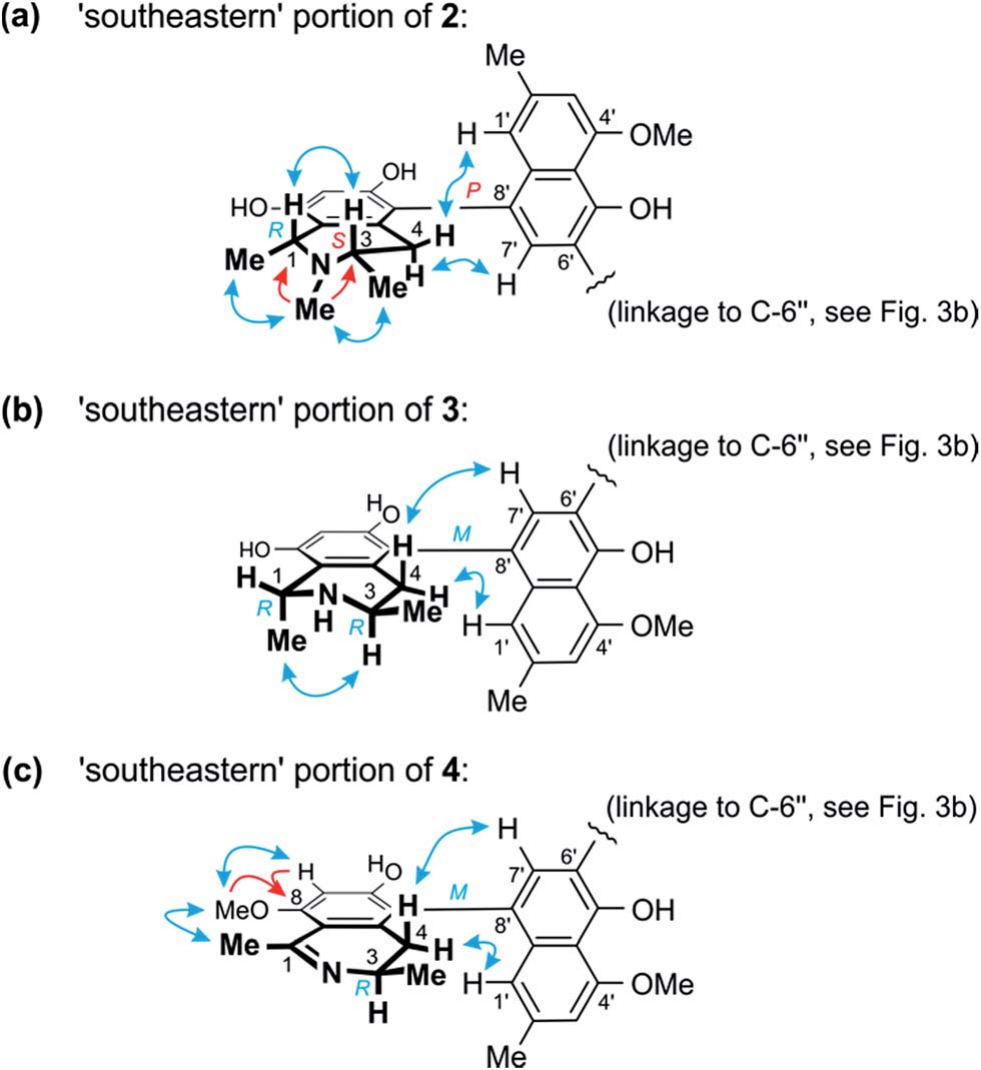
(a) Selected HMBC (red single arrows) and ROESY (blue double arrows) interactions within the ‘southeastern’ portion of 2 establishing its constitution and – together with the results of the oxidative degradation – absolute configuration (=korupensamine D, Fig. S1†). (b) ROESY correlations evidencing the relative and – based on the results of the oxidative degradation – absolute configurations in the southeastern part (=korupensamine B, 8a) of 3. (c) Key HMBC and ROESY for the ‘southeastern’ portion of 4 that, together with the results of the oxidative degradation, establish the absolute stereostructure of this portion (=the new compound ancistrobonsoline A_1_, 5).

Further 1D and 2D NMR data revealed that methyl group to be attached to the nitrogen atom in the ‘southeastern’ portion. Key evidence of the exact location of this *N*-methyl group were the HMBC correlations of its protons with both, C-1 and C-3 (Fig. 4a) and the signals of these methine carbons, which appeared deshielded (62.6 ppm for C-1 and 60.6 for C-3) as compared to those of the *N*-demethylated congeners – like, for example, in the case of 1 (Tables 1 and 2). This assignment was further supported by the ROESY series {Me-1 ↔ *N*–Me ↔ Me-3}, which, in addition, revealed a 1,3-diaxial relationship of H-1 and H-3 and, thus, a relative *cis*-configuration at the stereocenters C-1 and C-3 (Fig. 4a). For the stereocenters in the other, ‘northwestern’ part of 2, a relative *cis*-configuration was observed, too, again evidenced by the ROESY interactions of H-1′′′ with H-3′′′, as in 1 (Fig. 3b).

The ruthenium-mediated oxidative degradation of 2 yielded both, *R*- and *S*-configured 3-aminobutyric acids, showing that this alkaloid was either 3*R*,3′′′*S*- or 3*S*,3′′′*R*-configured. The simultaneous formation of the *N*-methylated analog of 3-aminobutyric acid only as its *S*-enantiomer, however, clearly indicated that the *S*-configured stereocenter was located next to the *N*-methyl group, *i.e.* at C-3. Thus, this compound was found to be 3*S*,3′′′*R*-configured, which, given the relative *cis*-configurations in both halves as mentioned above, showed that C-1 and C-1′′′ were *R*- and *S*-configured, respectively.

From ROESY interactions between H-4_ax_ and H-7′ and between H-4_eq._ and H-1′ (Fig. 4a), and based on the above-assigned *S*-configuration at C-3, the configuration at the southeastern axis was determined to be *P*, thus revealing this portion to correspond to korupensamine D, a known naphthylisoquinoline alkaloid from *A. korupensis* (for its structure, see Fig. S1†).^8^ At the other, northwestern axis of 2, the ROESY interactions were complementary: H-4′′′_ax_ with H-1′′ and H-4′′′_eq._ with H-7′′, which, together with the opposite *R*-configuration at C-3′′′ established above, indicated that axis to be *P*-configured, too, as in 1 (Fig. 3b). This metabolite thus had to be a cross-coupling product of ancistroealaine C (7) and korupensamine D (Fig. S1†),^8^ and possessed the absolute structure 2 presented in Fig. 1. It was named michellamine A_7_, due to its *P*,*P*-configured outer biaryl axes, as in 1. It is the second dimer of 5,8′-coupled naphthylisoquinolines with the unusual combination of two 1,3-*cis*-configured halves.

#### Michellamine B_4_ (3)

Compound 3, with a slightly longer retention time than those of 1 and 2 (Peak F, Fig. 2a), was revealed to be isomeric to 1 by mass spectrometry (HRESIMS: *m*/*z* 757.3475 [M + H]^+^, molecular formula: C_46_H_48_N_2_O_8_). Both, its ^1^H and ^13^C NMR spectra (Table 2) displayed full sets of signals, which showed that, different from 1, it consisted of two non-identical molecular halves.

The ^1^H NMR spectrum of 1 matched the half-set of signals of 3 nearly perfectly (Δ*δ*_H_ ≤ 0.02, see Tables 1 and 2), which suggested that the two compounds had one molecular portion in common. Further analysis of the 1D and 2D NMR data of 3 established the constitution of 3 to be identical to that of 1, that is, two 5,8′-coupled naphthylisoquinoline halves connected to each other *via* C-6′ (Fig. 3a).

In one portion, the northwestern part of 3, the relative configuration at the stereocenters C-1′′′ *versus* C-3′′′ was deduced to be *cis* from ROE interactions of H-1′′′ and H-3′′′, as also (even twice) in 1 (Fig. 3b). In the southeastern part of 3, however, the stereocenters were *trans*-configured, as evidenced from ROESY interactions between H-3 and Me-1 (Fig. 4b). The ruthenium-mediated oxidative degradation of 3 yielded 3-aminobutyric acid as its *R*-enantiomer exclusively, which demonstrated that both, C-3 and C-3′′′ were *R*-configured. This, together with the aforementioned *cis*- and *trans*-arrays, established the absolute configurations at C-1′′′ and C-1 to be *S* and *R*, respectively.

From ROESY interactions of H-4′′′_ax_ with H-1′′ and of H-4′′′_eq._ with H-7′′ and in view of the absolute *R*-configuration at C-3′′′, the absolute axial configuration in the northwestern half was assigned to be *P*, revealing this portion to be identical to 7, as in 1 (Fig. 3b). In the other, southeastern part, the respective protons interacted in a complementary way: H-4_ax_ with H-7′ and H-4_eq._ with H-1′, as shown in Fig. 4b. These correlations, together with the known *R*-configuration at C-3 (see above), evidenced the southeastern biaryl axis to be *M*-configured, thus showing the corresponding moiety to have the same structure as the likewise isolated korupensamine B (8b) (Fig. 4b). Therefore, compound 3 had to be the 6′,6′′-cross-coupling product of ancistroealaine C (7) and korupensamine B (8b), with the absolute stereostructure given in Fig. 1. It was named michellamine B_4_, in continuation of the series of michellamines with opposite configurations at the two outer biaryl axes (type B).

#### Michellamine B_5_ (4)

Compound 4, the slowest-eluting major metabolite of the aqueous fraction (Peak H, Fig. 2a), gave a monoprotonated molecule at *m*/*z* 769.3484 in HRESIMS, *i.e.* 12 units more than that of 3. Its ^1^H NMR spectrum displayed a full set of signals, with in particular one quartet (instead of two), thus indicating that this metabolite consisted of a naphthyl-1,3-dimethyl*tetra*hydro- and a naphthyl-1,3-dimethyldihydroisoquinoline subunit. In addition, this dimer had three methoxy functions (4.11, 4.10, and 4.03 ppm), *i.e.* one *O*-methyl group more than the other three compounds, 1–3. Comparison of its ^1^H NMR spectrum with those of 1–3 showed that these four metabolites had one common, nearly perfectly matching half-set of signals, corresponding to the northwestern portions of 1–3, which were equivalent to ancistroealaine C (7). The northwestern portion of 4 was therefore expected to be identical to 7, too, and its southeastern part was assumed to be the naphthyl-1,3-dimethyldihydroisoquinoline moiety, possessing two methoxy groups.

This assumption was corroborated by 1D and 2D NMR data, which led to a molecular architecture similar to that of 3, but with one additional methyl group attached to the oxygen function at C-8 and a double bond between C-1 and the adjacent nitrogen atom in the southeastern portion (Fig. 4c). Key NMR features of the location of the methoxy function at C-8 were the joint HMBC interactions from H-7 and OMe-8 to C-8 and the ROESY series {H-7 ↔ OMe-8 ↔ Me-1} (Fig. 4c).

The oxidative degradation procedure delivered only (*R*)-3-aminobutyric acid, showing that both, C-3 and C-3′′′ were *R*-configured. In the northwestern portion, ROESY interactions at the stereocenters C-1′′′ *versus* C-3′′′ and across the naphthalene–isoquinoline linkage were the same as in 1 (Fig. 3b), so that C-1 was *S*-configured and the biaryl axis had the *P*-configuration. In the southeastern portion, the ROESY interaction between H-4_ax_ and H-7′ indicated these two spin systems to be on the same side of the molecule, as depicted in Fig. 4c, which evidenced an *M*-configuration at the biaryl axis. Therefore, this metabolite possessed the absolute stereostructure 4, as shown in Fig. 1. It was named michellamine B_5_, due to its stereochemical similarity with michellamine B_4_ (3). Within a list of now 16 known natural michellamine-type dimers, it is only the third example that possesses a dihydroisoquinoline ring system; the other two analogs with such a structural peculiarity are michellamines F^10^ and A_4_.^9^

#### Ancistrobonsoline A_1_ (5)

The dichloromethane subfraction contained two major, nicely resolved metabolites (Fig. 2b). They were obtained as amorphous solids by resolution on a preparative reverse-phase HPLC column. The more polar one (Peak J, Fig. 2b), compound 5, gave an *m*/*z* at 392.1843 [M + H]^+^, corresponding to the molecular formula C_24_H_25_NO_4_. In the ^1^H NMR spectrum, the presence of two methoxy signals, together with the absence of a quartet around 4.5 ppm, which typically indicates a proton located at C-1, hinted at a naphthyl-1,3-dimethyldihydroisoquinoline alkaloid, presumably equivalent to the southeastern portion of 4. This was further corroborated by the fact that the signals in the aliphatic region of the ^1^H NMR spectrum of 5 matched very well with those assigned to the protons of the southeastern half of 4. Detailed analysis of the 1D and 2D NMR spectra of this monomeric alkaloid established it to have the same molecular skeleton as the southeastern portion of 4, yet with an additional proton at C-6′.

**Fig. 5:**
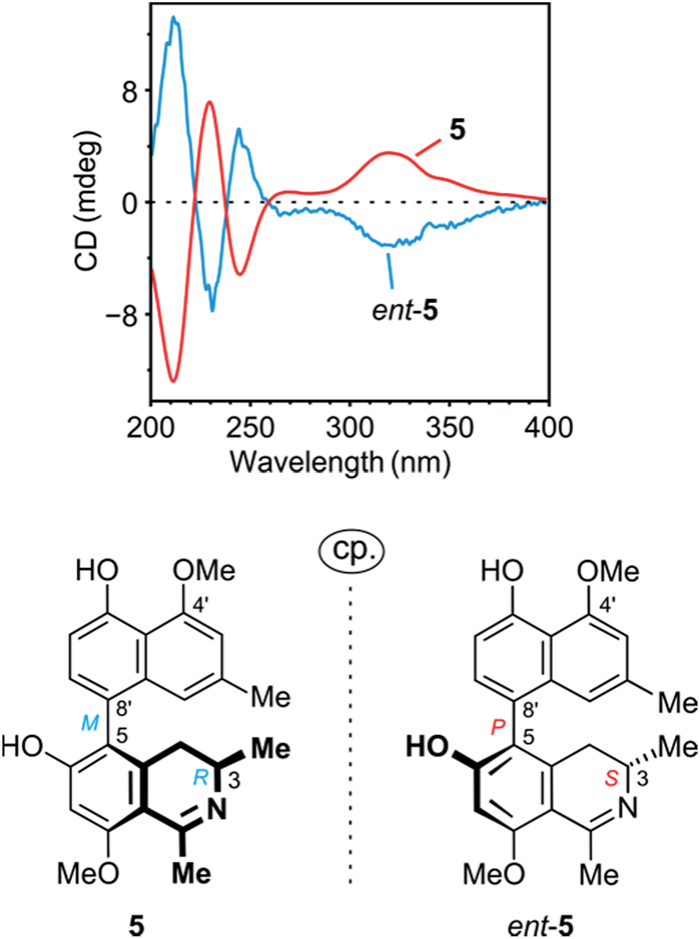
Comparison of the ECD spectrum of 5 with that of its previously known natural enantiomer, *ent*-5 (=6,5′-*O*,*O*-didemethylancistroealaine A),^15^ further confirming the absolute stereostructure of 5.

The ROESY interactions across the biaryl axis of 5 were all similar to those in the southeastern portion of 4 (see Fig. 4c), as were also the results of the oxidative degradation. This monomeric alkaloid thus possessed the absolute stereostructure 5, as presented in Fig. 1. It was, consequently, the as yet undescribed enantiomer of the known alkaloid 6,5′-*O*,*O*-didemethylancistroealaine A.^15^ In agreement with their opposite absolute configurations, their ECD spectra were fully mirror-image like (Fig. 5). Instead of the rational, but long name *ent*-6,5′-*O*,*O*-didemethylancistroealaine A, the new compound was named ancistrobonsoline A_1_, after the Congolese village Bonsolerive, where the plant had been collected.

#### Ancistrobonsoline A_2_ (6)

The sixth new compound discovered during these investigations (Peak I, Fig. 2b) was attributed the molecular formula C_25_H_27_NO_4_ according to the observation of its mono-protonated molecule at *m*/*z* 406.2013 in HRESIMS. Its ^1^H NMR spectrum was very similar to that of 5, indicating the presence of a 1,3-dimethyldihydroisoquinoline, yet with the signal of an additional *O*-methyl group at 3.83 ppm, linked either to *O*-6 or *O*-5′. NOE interactions of the protons of that *O*-methyl group with H-7, together with their joint HMBC correlations to C-6, clearly located that methoxy function at C-6.

The absolute configuration at the stereocenter C-3 of 6 was determined to be *R* by oxidative degradation, which, as in the case of 5, provided (*R*)-3-aminobutyric acid. Across the biaryl axis the specific relationships between the diastereotopic protons at C-4 and the aromatic protons H-1′ and H-7′ were the same as in 5 (Fig. 4c): H-4_ax_ (‘up’) interacted with H-7′ and H-4_eq._ (‘down’) with H-1′, which was consistent with an *M*-configuration at the 5,8′-axis. This absolute stereochemical assignment was further corroborated by the close similarity of the ECD spectrum of 6 with that of 5. Therefore, the new alkaloid had to possess the absolute stereostructure 6, as presented in Fig. 1, and was, thus, the 6-*O*-methylated derivative of 5. It was named ancistrobonsoline A_2_. With their *R*-configuration at the stereocenter and *M* at the biaryl axis, ancistrobonsolines A_1_ (5) and A_2_ (6) belong to a very small subgroup of naphthyldihydroisoquinolines displaying such stereochemical features, with previously only one single representative, ancistrolikokine F (Fig. S1†).^12^

### Chemotaxonomic and biosynthetic significance of the isolated metabolites

The molecular architectures of the alkaloids thus isolated from this as yet unidentified *Ancistrocladus* taxon are remarkable in many respects. Firstly, it is striking that all of these compounds belong to the same 5,8′-coupling type and, where dimers are concerned, they are all based on a central 6′,6′′-axis. Such a specific phenol-oxidative coupling – and, thus strict enzymic assistance – have so far been observed only in *A. likoko*.^11,12,16^

These findings thus reveal a close phylogenetic relationship between the two taxa (in addition to their aforementioned morphological similarities), but delineate one from the other by the fact that the naphthalene moieties of the metabolites produced by the plant from the proximity of Bonsolerive are, all of them, *O*-methylated only at C-4′ (and C-4′′, in the case of dimers), not at C-5′ (nor at C-5′′). And their configuration at C-3 (or/and C-3′′′, for dimeric compounds) is always *R*, except for the *N*-methylated isoquinoline portions, where an exclusive *S*-configuration is observed (see Fig. 1). Such a regioselective *O*-methylation in the naphthalene subunits and a stereospecificity at C-3 (and/or C-3′′′) are not known from *A. likoko* (or from any other related plant). Likewise demarcating these two taxa is the fact that *A. likoko* is a virtually exclusive producer of monomeric alkaloids (out of 21 metabolites, only two dimers have so far been isolated from this species),^12^ whereas the plant investigated here is a rich source of dimeric compounds.

Moreover, the remarkable occurrence of dimers with a symmetric 6′,6′′-coupling at the central axis chemotaxonomically delineates this taxon from the other – likewise still unidentified – liana that occurs in the same area.^6^ The latter is known to produce a drastically different metabolic pattern, including dimers with unsymmetric, 6′,1′′-coupled central axes,^17^ with additional oxygen bridges^18^ and/or acetal linkages,^21^ but so far no symmetric, 6′,6′′-coupled ones, not even in traces.

Secondly, it is particularly noteworthy that this liana produces the 1,3-*trans*-configured korupensamine A (8a) only in small quantities (Peak B, Fig. 2a) and shows no hints at the presence of its dimer, michellamine A (11), not even in traces.^20^ This is exceptional, since all of the plants known to produce michellamine-type alkaloids, *viz. A. korupensis*,^10,20^*A. congolesis*,^9^ and *A. likoko*,^12^ always contain michellamine A (11) as one of the main dimeric compounds. Moreover, 11 is always accompanied by its monomer, korupensamine A (8a), likewise occurring as a major metabolite in those plants.^9,10,20^ For this reason, it has even been suggested^9^ that other (minor) dimeric analogs differing from michellamine A (11) by the configurations at the stereocenters may originate from the preformed ‘parent’ dimer michellamine A (11), *i.e.* by modification after the dimerization step. In the case of the metabolites of the plant investigated here, however, one of the main constituents (Peak D, Fig. 2a), michellamine A_6_ (1), is twofold 1,3-*cis*-configured and can be regarded as a bis-epimer of michellamine A (11) at C-1 and C-1′′′. Since 1 is accompanied by an unusually large quantity of its – likewise *cis*-configured – monomer (Peak A, Fig. 2a), ancistroealaine C (=1-*epi*-korupensamine A) (7), it seems likely that 1 (as also 2) originates from the correspondingly preformed *cis*-monomers.

### Biological activities of the isolated metabolites

#### Antiprotozoal properties

Owing to the pronounced antiprotozoal activities of some naphthylisoquinoline alkaloids,^1,19,21–23^ the two new monomers ancistrobonsolines A_1_ (5) and A_2_ (6) were tested against the pathogens causing malaria (*Plasmodium falciparum*), human African sleeping sickness (*Trypanosoma brucei rhodesiense*), Chagas' disease (*T. cruzi*), and visceral leishmaniosis (*Leishmania donovani*). The other isolated new dimeric metabolites were not evaluated, since their analogs had previously been found inactive.^8,10^ As shown in Table 3, compounds 5 and 6 exhibited weak to moderate inhibitory properties. Of particular interest is that the results of 5 and 6 showed the OMe/OH substitution pattern to play a crucial role for the bioactivities: an *O*-methylation seems to be favorable for the antiplasmodial and antitrypanosomal activities, but disadvantageous for the antileishmanial activity. This finding complements previous results on the antileishmanial activities of related alkaloids, yet with *P*-configuration at the axis and *S* at C-3.^15^ Likewise interesting are the antiprotozoal activities of 5 and its enantiomer (Table 3), which are – in the case of *T. cruzi* – drastically different from one to another, documenting, once again, the impact of the absolute stereostructure.

**Table 3:** Antiprotozoal activities of the new naphthyldihydroisoquinoline alkaloids 5 and 6, and the previously reported data of the enantiomer of 5 (ref. 15)

Compound	IC_50_ (μM)					
	*P. falciparum*		*T. brucei rhodesiense* STIB900	*T. cruzi* Tulahuen C2C4 Lac Z	*L. donovani* axenic amastigotes MHOM/ET/67/L82	Cytotoxicity (L6 cells)
	K1	NF54				
5	2.7	2.1	44.4	109.3	87.9	114.9
*ent*-5^a^	5.4	n. d	24.8	16.3	43.4	>230
6	1.8	2.4	12.6	80.1	246.6	52.8
Standard	0.31^b^	0.01^b^	0.04^c^	5.69^d^	1.08^e^	0.01^f^

a Values reported earlier (see, ref. 15).

b Chloroquine.

c Melarsoprol.

d Benznidazole.

e Miltefosine.

f Podophyllotoxin.

#### Anti-HeLa potential: cytotoxicity and effects on cell morphology

As part of our ongoing investigations on the potential of naphthylisoquinoline alkaloids towards different cancer cell lines,^12,24–26^ the isolated compounds were tested for their cytotoxic activity against HeLa human cervical cancer cells (Table 4). Interestingly, the new twofold 1,3-*cis*-configured dimeric compounds, michellamines A_6_ (1) and A_7_ (2), and the likewise new naphthyldihydroisoquinoline alkaloids, ancistrobonsolines A_1_ (5) and A_2_ (6), displayed strong cytotoxic activity, with IC_50_ values between 14.8 and 21.5 μM. The most potent cytotoxicity (IC_50_ = 8.8 μM) was displayed by michellamine E (10), which was even more active than the positive control 5-fluorouracil (IC_50_ = 13.9 μM), an anticancer drug in clinical use.^27^

**Table 4:** Growth-inhibitory activities of the isolated compounds 1–10 against HeLa cervical cancer cells (IC_50_) and, following the antiausterity strategy, against the human PANC-1 pancreatic cancer cell line (PC_50_)

Compound	HeLa (IC_50_ in μM)	PANC-1^a^ (PC_50_ in μM)
1	14.8	54.2
2	20.6	24.3
3	46.3	50.3
4	29.8	60.2
5	14.3	7.5
6	21.5	12.1
7	30.5	>100
8a	48.3	>100
8b	37.8	94.9
9	32.1	19.3
10	8.8	18.9
Standard	13.9^b^	0.8^c^

a Concentration at which 50% of the PANC-1 pancreatic cancer cells were killed preferentially in nutrient-deprived medium (NDM).

b 5-Fluorouracil.

c Arctigenin.

**Fig. 6:**
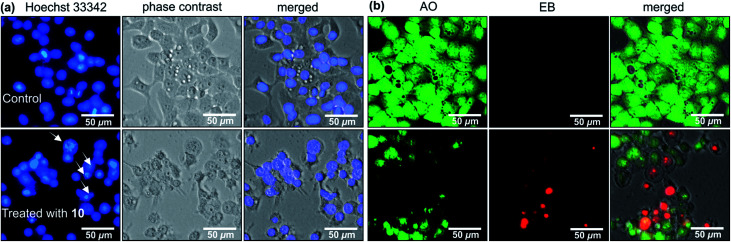
Morphological changes of HeLa human cervical cancer cells induced by 12.5 μM of michellamine E (10) in comparison to untreated control: (a) Hoechst 33342 nuclei staining and (b) acridine orange (AO) – ethidium bromide (EB) staining.

Michellamine E (10) was, therefore, investigated for its effects on cell morphology and apoptosis using two distinct staining methods, the Hoechst 33342 staining and the ethidium bromide–acridine orange (EB–AO) double staining assay. In the Hoechst 33342 staining, the dye penetrates through the cell membrane and intercalates with DNA and emits blue fluorescence.^28^ As shown in Fig. 6a, untreated HeLa cells (the control) displayed regular cell morphology with the intact nuclei. However, treatment with 12.5 μM of 10 induced nuclear fragmentation, suggestive of cells undergoing apoptosis, as indicated by fragmented nuclei exemplarily shown by white arrows in Fig. 6a. The EB–AO assay, on the other hand, allows visualizing the cellular morphology as well as the distinction between the live and dead cells. Acridine orange (AO) is a cell-membrane permeable dye emitting bright-green fluorescence in live cells, and ethidium bromide (EB) penetrates only the membrane of dying or dead cells staining them red.^29^ As shown in Fig. 6b, untreated HeLa cells (the control) displayed intact, regular cell morphology with exclusive bright green fluorescence in AO–EB staining. Treatment of the tumor cells with 12.5 μM of 10, however, disrupted the cellular integrity leading to rounding of the cell membrane, membrane rupture, and disintegration of cellular contents resulting in an increased population of red EB fluorescence of dead cells (Fig. 6b).

#### Antiausterity activities: preferential cytotoxicity against PANC-1 human cancer cell line and effects on cell morphology

Following the antiausterity strategy as recently developed,^30–32^ the isolated naphthylisoquinoline alkaloids 1–10 were tested against PANC-1 pancreatic cancer cells. Pancreatic tumors are hypovascular (limited blood vessels) in nature and are, consequently, constantly exposed to nutrient and oxygen starvation in their microenvironment. However, pancreatic cancer cells show a remarkable tolerance for nutrition starvation, allowing them to adapt and survive in such inadequate supply of nutrients.^30^ Discovery of anticancer agents by targeting this tolerance of nutrition starvation is the key approach of antiausterity strategy in anticancer drug discovery. The new dimer michellamine A_7_ (2) and its known analogs michellamines A_2_ (9) and E (10) exhibited significant preferential cytotoxicities against PANC-1 cells in a concentration-dependent manner (Fig. S2†). Their PC_50_ values (*i.e.* the concentration at which 50% of the cells are preferentially killed under nutrient-deprived conditions, without cytotoxicity in normal, nutrient-rich medium) ranged from 18.9 to 24.3 μM, but were surpassed by the even higher activities of the new monomeric compounds ancistrobonsolines A_1_ (5) and A_2_ (6), with PC_50_ values of 7.5 and 12.1 μM, respectively (Table 4).

**Fig. 7:**
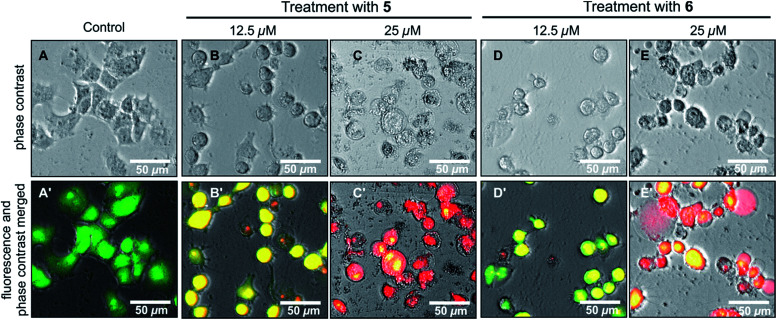
Morphological changes of PANC-1 human pancreatic cancer cells induced by the new compounds ancistrobonsolines A_1_ (5) and A_2_ (6) in comparison to the untreated ones (control).

The effects of ancistrobonsolines A_1_ (5) and A_2_ (6), as representatives of the compounds showing potent antiausterity activities, on the cell morphology and apoptosis were further investigated by the ethidium bromide (EB) – acridine orange (AO) double-staining fluorescence assay as described above. As shown in Fig. 7(A and A′), the untreated PANC-1 cells, serving as the control, emitted the typical bright-green color, characteristic of living cells, exhibiting an intact cellular morphology. This, however, changed significantly when the cells were treated with 12.5 μM of 5 and 6, as revealed by the yellow fluorescence resulting from the overlapping of light emitted by AO (green) and EB (red) (Fig. 7B′ and D′). With 25 μM, both compounds induced a dramatic morphological alteration and disintegration of cellular organelles, leading to total cell death as illustrated by the virtually exclusive red stain in Fig. 7(C′ and E′). All these results suggest that naphthylisoquinoline alkaloids are promising lead structures for anticancer drug development.

## Experimental section

### General experimental procedures

UV/Vis spectra were measured with a Shimadzu UV-1800 spectrophotometer, IR spectra with a Jasco FT-IR-4600 type A spectrometer, and optical rotations with a Jasco P-1020 polarimeter. ECD spectra were obtained on a J-715 spectropolarimeter (Jasco) at room temperature, using a standard cell (0.02 cm) and spectrophotometric-grade methanol, and are reported in Δ*ε* values (cm^2^ mol^−1^) at the given wavelength *λ* (nm). GC-MSD analyses were performed on a GCMS-QP 2010SE (Shimadzu). 1D and 2D NMR spectra were recorded on Bruker Avance III HD 400 (400 MHz) and 600 (600 MHz) instruments in deuterated methanol. Chemical shifts (*δ*) are reported in parts per million (ppm) with the ^1^H and ^13^C signals of the solvent (^1^H, *δ* = 3.31 ppm; ^13^C, *δ* = 49.15 ppm) as the internal reference. HRESIMS spectra were obtained on a microTOF-focus and micrOTOF-Q III mass spectrometers (Bruker). Preparative HPLC separation was performed on a Jasco HPLC system (PU-2087, UV-2077, LC-NetII/ADC), using a SymmetryPrep C18 column (Waters, 19 × 300 mm, 7 μm) with the UV absorption wavelengths set at 232, 254, and 310 nm. Organic solvents were analytical grade or distilled prior to use.

### Plant material

*Ancistrocladus* plant material, morphologically related to those of *A. likoko*, were collected in August 2015 (Global Positioning System coordinates: 00°06.572S, 018°20.146E, 315 ± 22 m) by one of us (B. K. L.) in the vicinity of the village Bonsolerive, which is located at about 20 km southeast of the town of Mbandaka in the Democratic Republic of the Congo. A voucher specimen (No. 105) has been deposited at the Herbarium Bringmann, Institute of Organic Chemistry, University of Würzburg.

### Extraction and isolation

Air-dried leaves (200 g) were ground and repeatedly extracted by maceration with a neutral mixture of MeOH – CH_2_Cl_2_ (1 : 1, 2 L) with mechanical shaking (160 RPM). After three cycles of 24 h each, the filtrates were evaporated to dryness and the marc was extracted with an acidified (HCl) mixture of MeOH – CH_2_Cl_2_ (1 : 1, pH = 2–3, 2 L), again with mechanical shaking, for 48 h. The acidified extract was neutralized with NaOH and mixed with the neutral one, after the similarity between the two extracts was established by analytical HPLC. The resulting total crude residue was submitted to a cation-exchange column (Amberlyst-15 from Fluka, *Ø* 3 cm) to remove undesired, non-basic metabolites.^21^ The obtained alkaloid-enriched fraction was partitioned between water and dichloromethane, giving, after evaporation, 800 mg of an aqueous subfraction and 30 mg of dichloromethane subfraction. The polar subfraction was dissolved in methanol and resolved by preparative HPLC, using the following gradient: A/B: 0 min 10% B; 25 min 25% B; 30 min 28% B; 31 min 100% B; 34 min 100% B; 35 min 10% B, in which *A* = 90% H_2_O + 0.05% trifluoroacetic acid and *B* = 90% MeCN + 0.05% trifluoroacetic acid. From this subfraction, eight metabolites were obtained, including the alkaloids 1–3 and 6–10. Resolution of the lipophilic subfraction by preparative HPLC yielded compounds 4 and 5.

#### Michellamine A_6_ (1)

[*α*]^25^_D_: −35 (*c* = 0.01, MeOH); UV (MeOH) *λ*_max_ nm: 200, 228, 263, 302, 311, 316, 331, 333, 344; ECD (MeOH) cm^2^ mol^−1^: Δ*ε*_212_ −29.1, Δ*ε*_247_ +15.2, Δ*ε*_297_ −11.8, Δ*ε*_331_ +5.1; IR (ATM): *ν*_max_ cm^−1^: 3329, 1672, 1600, 1418, 1359, 1249, 1181, 1138, 1071, 836, 799, 722; HRESIMS: *m*/*z* 757.3486 [M + H]^+^ (calcd for C_46_H_49_N_2_O_8_: 757.3483). For ^1^H and ^13^C NMR data, see Table 1.

#### Michellamine A_7_ (2)

[*α*]^25^_D_: −41 (*c* = 0.08, MeOH); UV (MeOH) *λ*_max_ nm: 203, 229, 263, 302, 311, 316, 331, 333, 345; ECD (MeOH) cm^2^ mol^−1^: Δ*ε*_210_ −26.5, Δ*ε*_243_ +23.3, Δ*ε*_300_ −14.3, Δ*ε*_340_ +4.8; IR (ATM): *ν*_max_ cm^−1^: 3329, 1672, 1600, 1418, 1359, 1249, 1181, 1138, 1071, 836, 799, 722; HRESIMS: *m*/*z* 771.3655 [M + H]^+^ (calcd for C_47_H_51_N_2_O_8_: 771.3640). For ^1^H and ^13^C NMR data, see Table 2.

#### Michellamine B_4_ (3)

[*α*]^25^_D_: −25 (*c* = 0.03, MeOH); UV (MeOH) *λ*_max_ nm: 201, 229, 264, 302, 311, 316, 330, 333, 343; ECD (MeOH) cm^2^ mol^−1^: Δ*ε*_207_ +2.0, Δ*ε*_230_ −5.7, Δ*ε*_316_ +1.0, Δ*ε*_353_ −0.4; IR (ATM): *ν*_max_ cm^−1^: 3335, 1672, 1617, 1418, 1360, 1251, 1200, 1137, 1072, 837, 800, 722; HRESIMS: *m*/*z* 757.3475 [M + H]^+^ (calcd for C_46_H_49_N_2_O_8_: 757.3483). For ^1^H and ^13^C NMR data, see Table 2.

#### Michellamine B_5_ (4)

[*α*]^25^_D_: −32 (*c* = 0.008, MeOH); UV (MeOH) *λ*_max_ nm: 204, 231, 266, 301, 311, 317, 332, 345; ECD (MeOH) cm^2^ mol^−1^: Δ*ε*_210_ −22.0, Δ*ε*_229_ −8.0, Δ*ε*_237_ −10.1, Δ*ε*_262_ +14.1, Δ*ε*_304_ −9.0, Δ*ε*_327_ −6.7, Δ*ε*_350_ −11.1; IR (ATM): *ν*_max_ cm^−1^: 3354, 1670, 1625, 1577, 1408, 1354, 1326, 1262, 1199, 1132, 1071, 957, 832, 798, 720; HRESIMS: *m*/*z* 769.3484 [M + H]^+^ (calcd for C_47_H_49_N_2_O_8_: 769.3483). For ^1^H and ^13^C NMR data, see Table 2.

#### Ancistrobonsoline A_1_ (5)

[*α*]^25^_D_: +75 (*c* = 0.01, MeOH); UV (MeOH) *λ*_max_ nm: 336, 325, 315, 312, 236;ECD (MeOH) cm^2^ mol^−1^: Δ*ε*_211_ −10.1, Δ*ε*_229_ + 5.7, Δ*ε*_244_ −2.9, Δ*ε*_269_ +0.6, Δ*ε*_281_ +0.5, Δ*ε*_320_ +2.8; IR (ATM): *ν*_max_ cm^−1^: 3382, 1671, 1625, 1575, 1400, 1352, 1326, 1297, 1264, 1198, 1132, 1084, 956, 832, 799, 720; HRESIMS: *m*/*z*: 392.1843 [M + H]^+^ (calcd for C_24_H_26_NO_4_: 392.1856). For ^1^H and ^13^C NMR data, see Table 1.

#### Ancistrobonsoline A_2_ (6)

[*α*]^25^_D_: +56 (*c* = 0.009, MeOH); UV (MeOH) *λ*_max_ nm: 336, 325, 315, 313, 237; ECD (MeOH) cm^2^ mol^−1^: Δ*ε*_210_ −12.1, Δ*ε*_229_ +4.7, Δ*ε*_242_ −1.9, Δ*ε*_262_ +1.6, Δ*ε*_286_ +0.6, Δ*ε*_314_ +2.6; IR (ATM): *ν*_max_ cm^−1^: 3384, 1670, 1626, 1577, 1457, 1263, 1200, 1130, 1084, 1052, 956, 834, 798, 719; HRESIMS: *m*/*z*: 406.2013 [M + H]^+^ (calcd for C_25_H_28_NO_4_: 406.2013). For ^1^H and ^13^C NMR data, see Table 1.

#### Known alkaloids isolated, 7–10

The likewise isolated mono- and dimeric alkaloids, ancistroealaine C (7), korupensamines A (8a) and B (8b), and michellamines A_2_ (9) and E (10) were found to be identical in their spectroscopic, physical, and/or chromatographic properties with data previously reported.^7–10^

### Ruthenium-mediated oxidative degradation

Following a procedure described earlier,^14^*ca.* 0.8 mg of the compounds 1–10 were submitted to a ruthenium(iii)-catalyzed periodate oxidation, followed by derivatization of the resulting amino acids with MeOH/HCl and (*R*)-α-methoxy-α-trifluoromethylphenylacetyl chloride [(*R*)-MTPA-Cl, prepared from (*S*)-MTPA] and analyzed by GC-MSD.

### Antiprotozoal activities

The assessment of the antiprotozoal properties of the compounds 5 and 6 against *Plasmodium falciparum* (NF54 and K1), *Trypanosoma cruzi* (Tulahuen C2C4 with the Lac Z gene incorporated) amastigotes in mouse macrophages, *Trypanosoma brucei rhodesiense* (STIB900) bloodstream stages, and *Leishmania donovani* (MHOM/ET/67/L82) axenic amastigotes and the testing of the cytotoxicity against mammalian host cells (rat skeletal myoblast L6 cells) were done *in vitro* as previously described.^33^

### Cytotoxicity assay

The cytotoxicity assays against HeLa cell line (RCB0007, Tsukuba, Japan) were carried out as described previously.^24^ In brief, exponentially growing cells were harvested and plated in 96-well plates (2 × 10^3^ per well) in DMEM and allowed to attach for 24 h in the humidified CO_2_ incubator at 37 °C. The cells were then washed with phosphate-buffered saline (PBS) followed by the addition of serially diluted test samples in DMEM. For each concentration, three replications were performed. After 72 h of incubation, the cells were washed twice with PBS, and 100 μL of DMEM containing 10% WST-8 cell counting kit (Dojindo Molecular Technologies, Inc., Rockville, MD, USA) solution were added. After 3 h of incubation, the absorbance at 450 nm was measured on an EnSpire Multimode plate reader (PerkinElmer, Inc., Waltham, MA, USA). Cell viability was calculated from the mean values from three wells using the following equation:Cell viability (%) = [(Abs_test sample_ − Abs_blank_)/(Abs_control_ − Abs_blank_)] × 100%

### Antiausterity assay

The human pancreatic cancer PANC-1 (RBRC-RCB2095, Tsukuba, Japan) cell line was purchased from the Riken BRC cell bank and maintained in the standard DMEM with 10% FBS supplement under a humidified atmosphere of 5% CO_2_ in the incubator at 37 °C. For the antiausterity evaluation, exponentially growing cells were seeded in 96-well plates (1.5 × 10^4^ per well) in DMEM and incubated for 24 h for the cell attachment. After this incubation time, the cells were washed twice with PBS, the medium was changed to serially diluted test samples in both nutrient-rich medium (DMEM) and nutrient-deprived medium (NDM) with a control and a blank in each test plate. The composition of the NDM was as follows: 0.1 mg L^−1^ Fe(NO_3_)_3_ (9H_2_O), 265 mg L^−1^ CaCl_2_ (2H_2_O), 400 mg L^−1^ KCl, 200 mg L^−1^ MgSO_4_ (7H_2_O), 6400 mg L^−1^ NaCl, 700 mg L^−1^ NaHCO_3_, 125 mg L^−1^ NaH_2_PO_4_, 15 mg L^−1^ phenol red, 25 mM L^−1^ HEPES buffer (pH 7.4), and MEM vitamin solution (Life Technologies, Inc., Rockville, MD, USA); the final pH was adjusted to 7.4 with 10% aqueous NaHCO_3_. After 24 h of incubation with the respective test compound in DMEM and NDM, the cells were washed twice with PBS and replaced by 100 μL of DMEM containing 10% WST-8 cell counting kit solution. After 3 h of incubation, cell viability was measured and calculated as described above.

### Morphological assessment of cancer cells

For studies on morphological changes, HeLa and PANC-1 cells were seeded in 24-well plates (1 × 10^5^) and incubated in a humidified CO_2_ incubator for 24 h for the cell attachment. The cells were then washed twice with PBS and treated with vehicle control or test compounds in DMEM for HeLa cells; and vehicle control or test compounds in NDM for PANC-1 cells, and incubated for 24 h. For nuclei staining, two drops of the NucBlue® ready probe (Hoechst 33342) was directly added to the cells in full media and was incubated for further 25 min at the end of the experiment. For cellular morphology, 10 μL of EB/AO reagent (dye mixture: 100 μg mL^−1^ AO and 100 μg mL^−1^ EB in PBS) was added to each test well and further incubated for 10 min. The cellular images were captured in the fluorescent and phase contrast modes, using an EVOS FL digital microscope (20× objectives).

## Conclusions

In summary, this paper describes the discovery of the first twofold 1,3-*cis*-configured michellamine-type dimers, 1 and 2, and of four other mono- and dimeric naphthylisoquinoline alkaloids, 3–6, in the leaves of an as yet unidentified Congolese *Ancistrocladus* liana related to *A. likoko*. These new compounds, obtained along with five previously described analogs, 7–10, chemotaxonomically delineate this Congolese plant from all known related taxa, thus suggesting that it is probably a new species. Further taxonomic investigations, including DNA analysis, are planned. Some of the isolated metabolites have shown attractive cytotoxicities against the HeLa cell line and very good antiausterity activities against PANC-1 human pancreatic cancer cells. The weak-to-moderate antiprotozoal properties of the new monomeric metabolites 5 and 6 highlight the consequence of the stereochemical features on the biological activities and provide valuable information for the ongoing SAR investigations.

## Conflicts of interest

There are no conflicts to declare.

## Supplementary Material

RA-008-C8RA00363G-s001
