# Supplementary material for: Michellamines A6 and A7, and further mono- and dimeric naphthylisoquinoline alkaloids from a Congolese Ancistrocladus liana and their antiausterity activities against pancreatic cancer cells
Source: RSC Adv. 2018 Jan 31;8(10):5243–54. doi: 10.1039/c8ra00363g (PMC9078195; doi:10.1039/c8ra00363g)
Supplement: RA-008-C8RA00363G-s001 [file RA-008-C8RA00363G-s001.pdf]

Electronic Supplementary Material (ESI) for RSC Advances.
This journal is © The Royal Society of Chemistry 2018

Electronic Supplementary Information for

Michellamines A_6 and A_7, and further mono- and dimeric naphthylisoquinoline alkaloids from a Congolese *Ancistrocladus* liana and their antiausterity activities against pancreatic cancer cells

Blaise Kimbadi Lombe,[a,b] Doris Feineis,[a] Virima Mudogo,[b] Reto Brun,[c,d] Suresh Awale*[e] and Gerhard Bringmann*[a]

[a]*Institute of Organic Chemistry, University of Würzburg, Am Hubland, D-97074 Würzburg, Germany. Email: bringman@chemie.uni-wuerzburg.de; Fax: +49 931 31 84755*

[b]*Faculté des Sciences, Université de Kinshasa, B.P. 202, Kinshasa XI, Democratic Republic of the Congo*

[c]*Swiss Tropical and Public Health Institute, Socinstrasse 57, CH-4002 Basel, Switzerland*

[d]*University of Basel, Petersplatz 1, CH-4003 Basel, Switzerland*

[e]*Division of Natural Drug Discovery, Institute of Natural Medicine, University of Toyoma, 2630 Sugitani, Toyama 930-0194, Japan. Email: suresh@inm.u-toyama.ac.jp*

Table of contents

1. Structures of korupensamine D and ancistrolikokine F P1
2. Preferential cytotoxicities of compounds **2**, **5**, **6**, **9**, and **10** P1
3. Supplementary material for michellamine A_6 (**1**)
   - NMR spectra in methanol-d_4 P2
   - HRESIMS spectrum P9
   - CD spectrum in methanol P10
   - IR spectrum P11
   - Oxidation degradation P12
4. Supplementary material for michellamine A_7 (**2**)
   - NMR spectra in methanol-d_4 P13
   - HRESIMS P20
   - CD spectrum in methanol P21
   - IR spectrum spectrum P22
   - Oxidation degradation P23
5. Supplementary material for michellamine B_4 (**3**)
   - NMR spectra in methanol-d_4 P24
   - HRESIMS spectrum P31
   - CD spectrum in methanol P32
   - IR spectrum P33
   - Oxidation degradation P34
6. Supplementary material for michellamine B_5 (**4**)
   - NMR spectra in methanol-d_4 P35
   - HRESIMS spectrum P42
   - CD spectrum in methanol P43
   - IR spectrum P44
   - Oxidation degradation P45
7. Supplementary material for ancistrobonsoline A_1 (**5**)
   - NMR spectra in methanol-d_4 P46
   - HRESIMS spectrum P52
   - CD spectrum in methanol P53
   - IR spectrum P54
   - Oxidation degradation P55
8. Supplementary material for ancistrobonsoline A_2 (**6**)
   - NMR spectra in methanol-d_4 P56
   - HRESIMS spectrum P63
   - CD spectrum in methanol P64
   - IR spectrum P65
   - Oxidation degradation P66

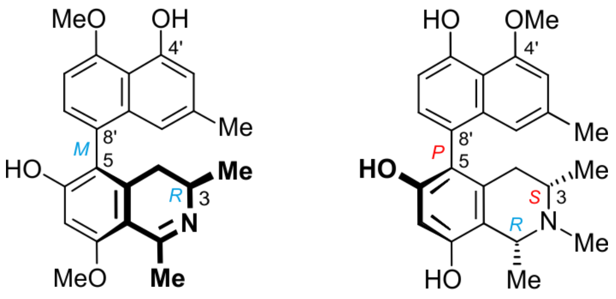

1 **2**

Fig. S1 Structures of the already known naphthylisoquinolines ancistrolikokine F (**1**) and korupensamine D (**2**).

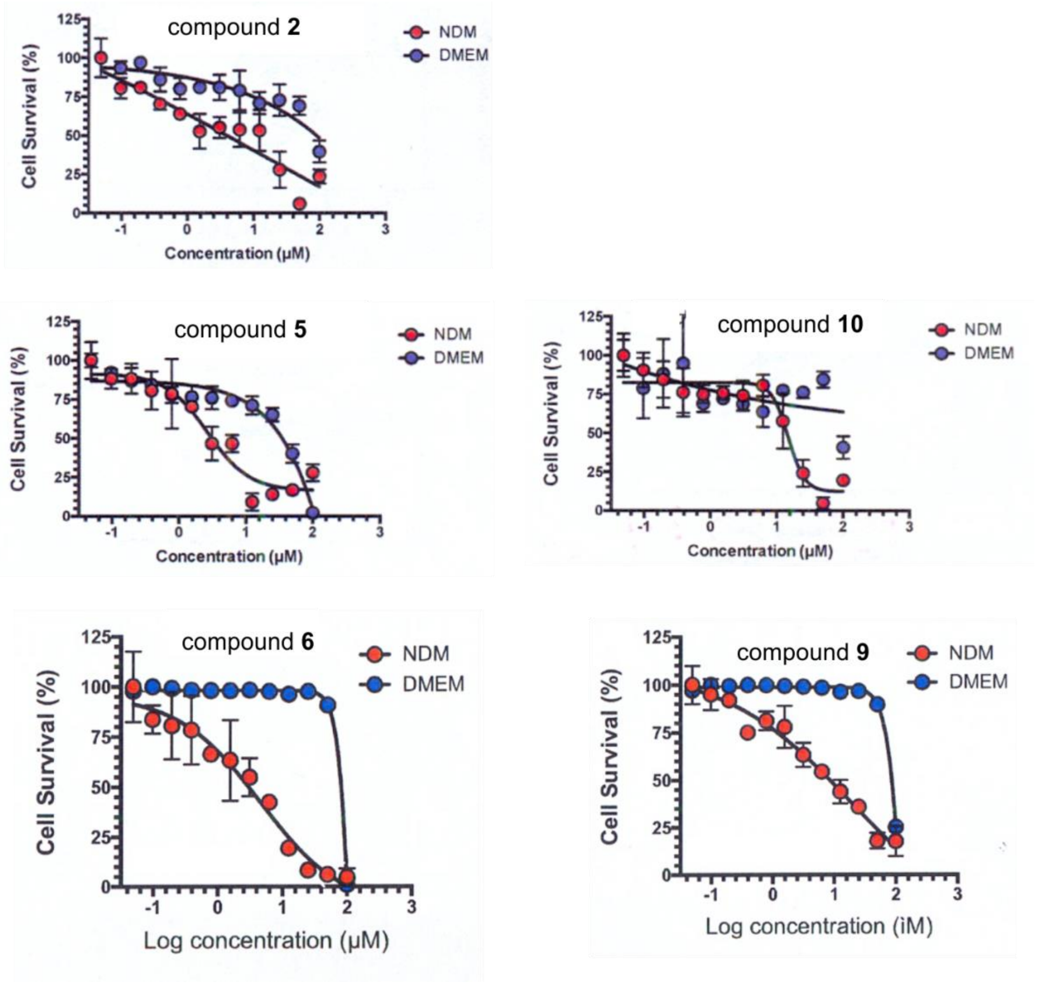

Fig. S2 Preferential cytotoxic activity of michellamines A_7 (**2**), A_2 (**9**), and E (**10**), and ancistrobonsolines A_1 (**5**) and A_2 (**6**) against the PANC-1 human pancreatic cancer cell line in nutrient-deprived medium (NDM) and Dulbecco's modified Eagle's medium (DMEM)

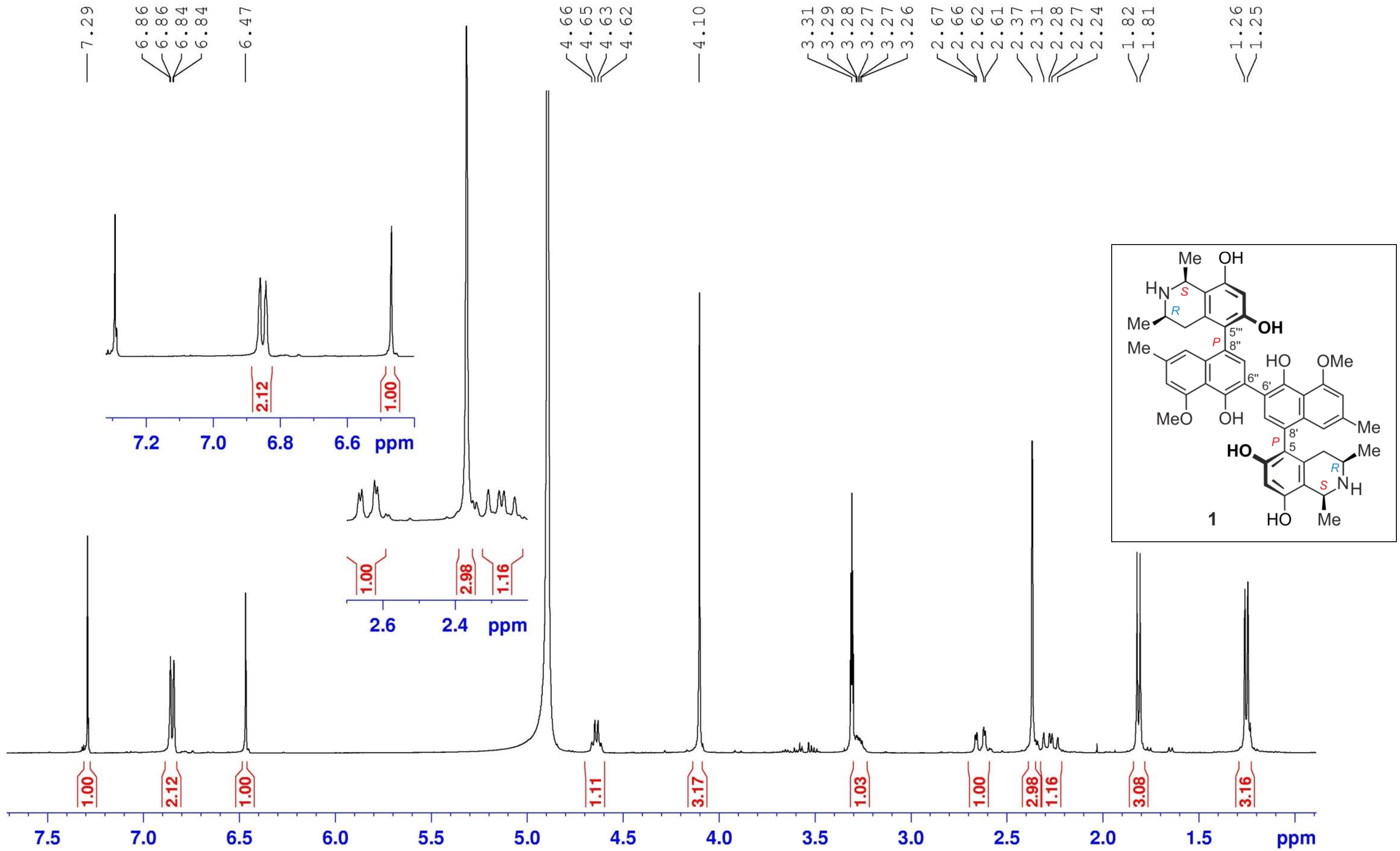

Fig. S3 ^{1}H NMR spectrum of compound **1** in methanol-d_4

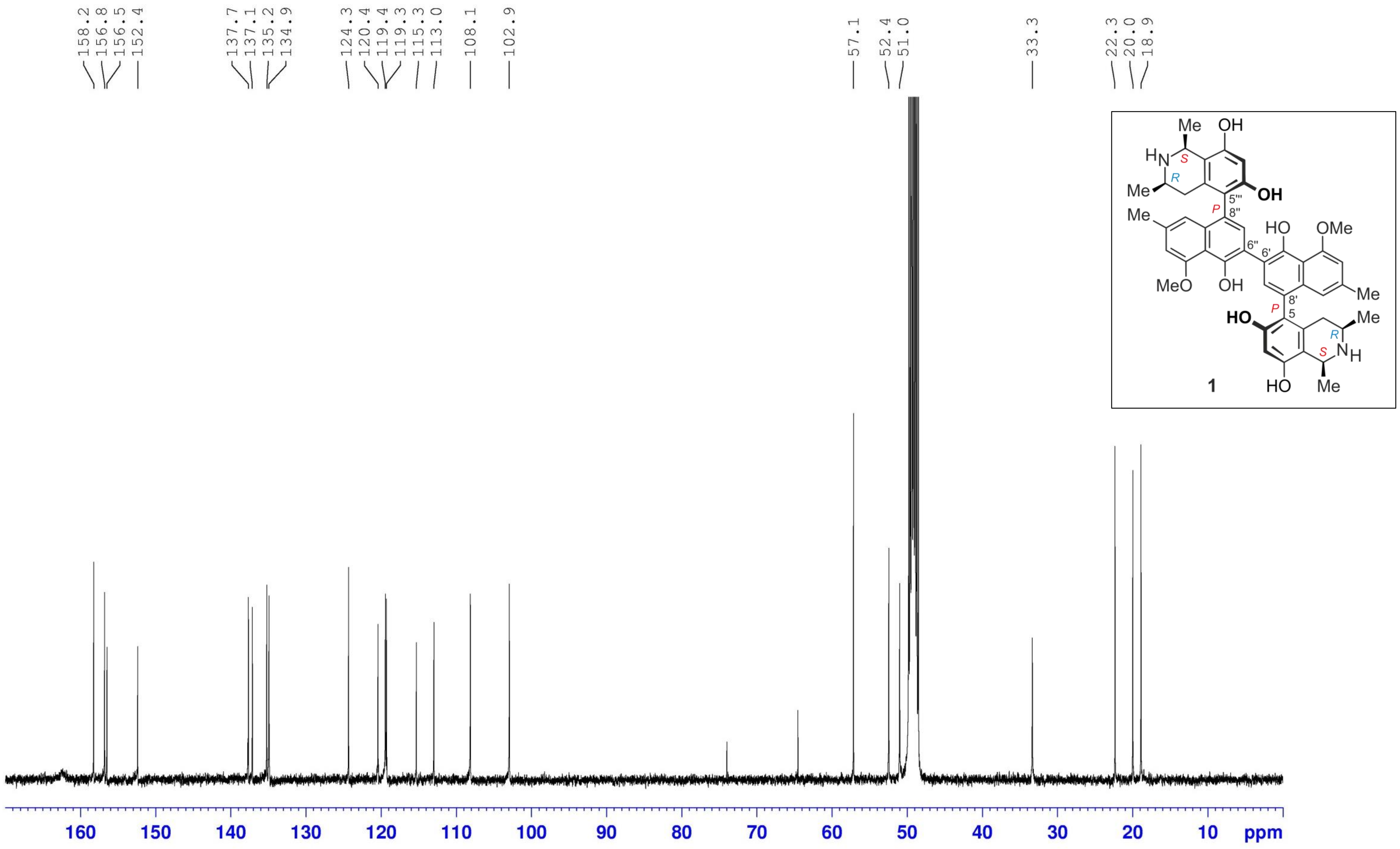

Fig. S4 ^{13}C NMR spectrum of compound **1** in methanol-d_4

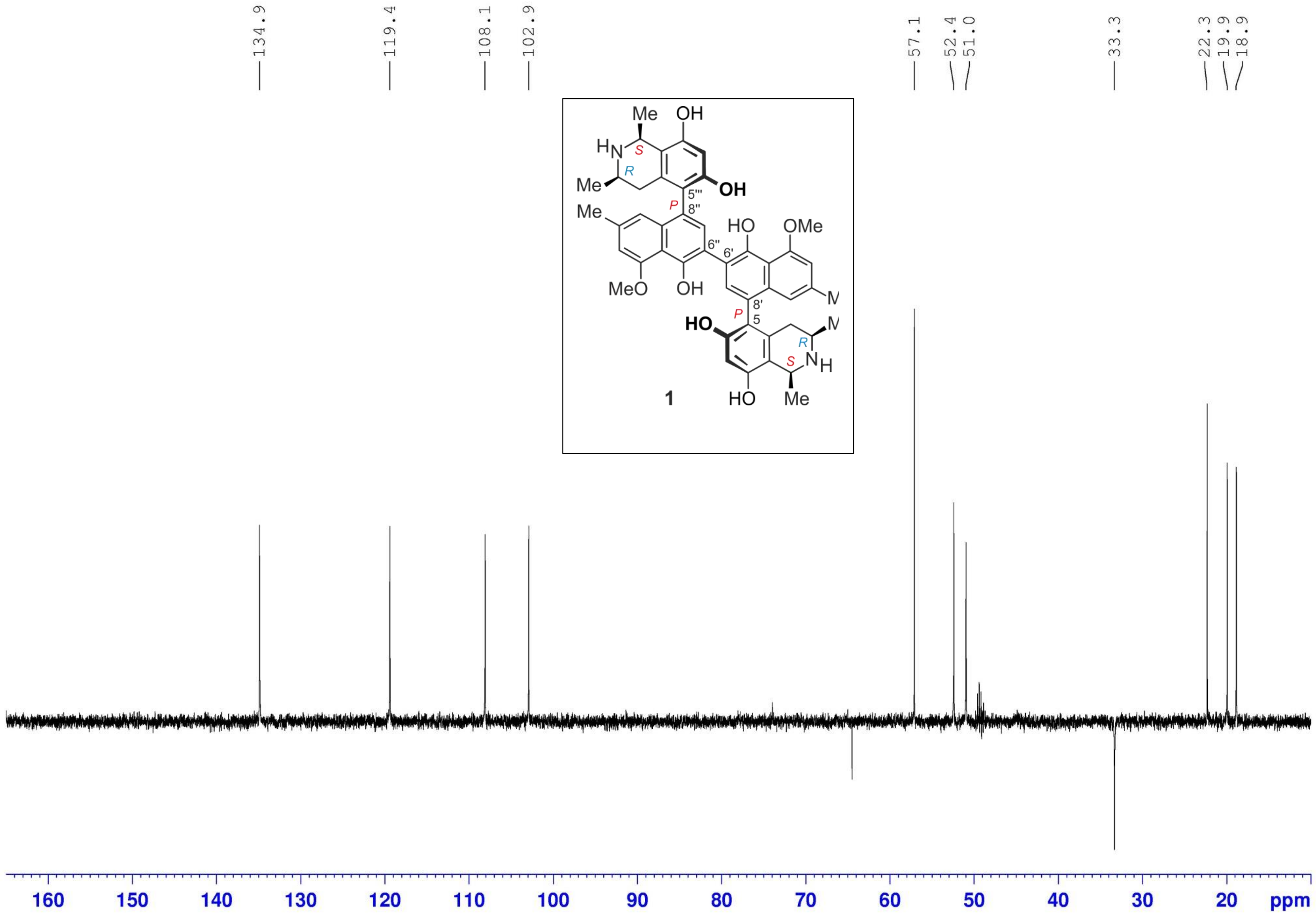

Fig. S5 DEPT-135 NMR spectrum of compound **1** in methanol-d_4

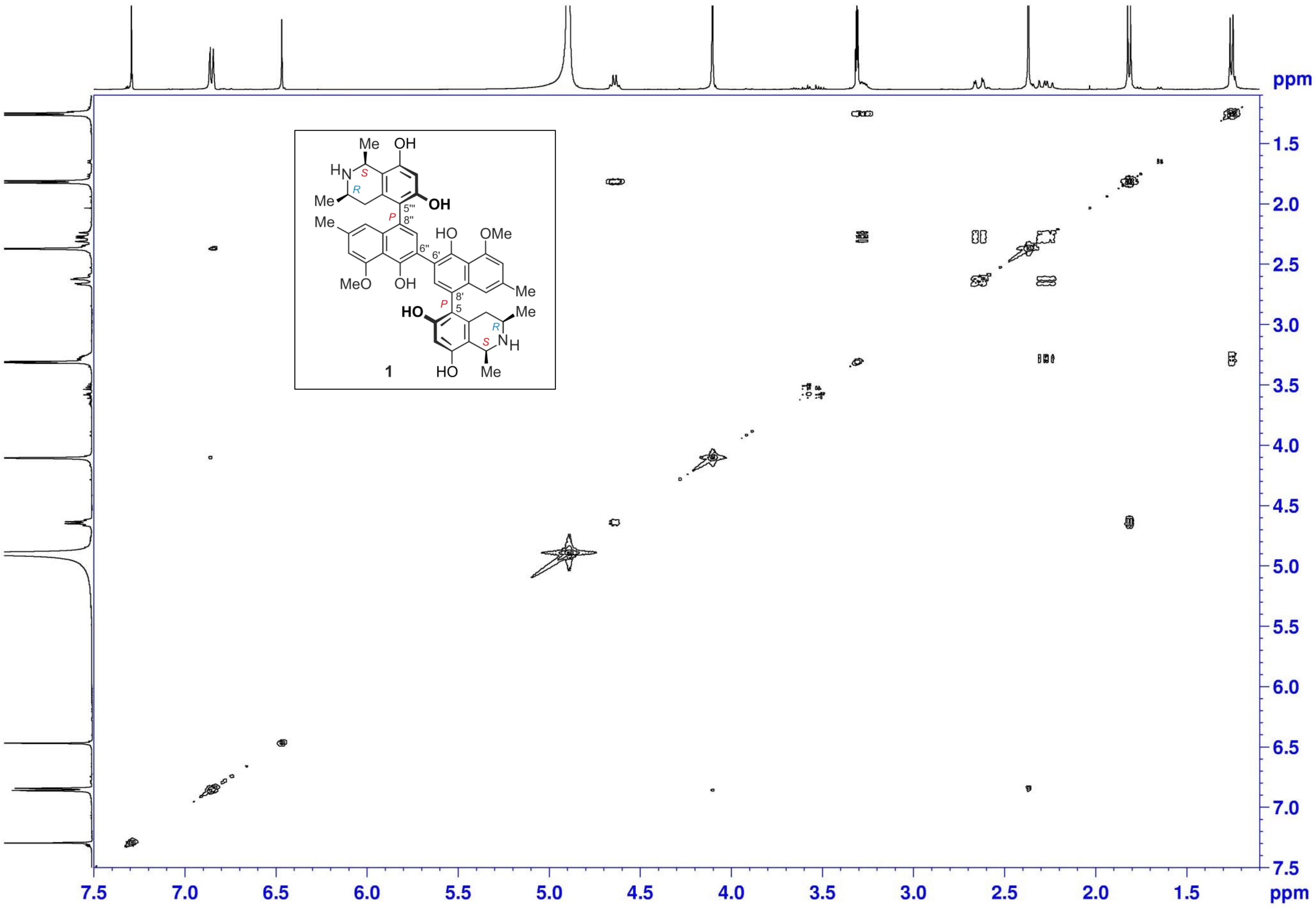

Fig. S6 COSY NMR spectrum of compound **1** in methanol-d_4

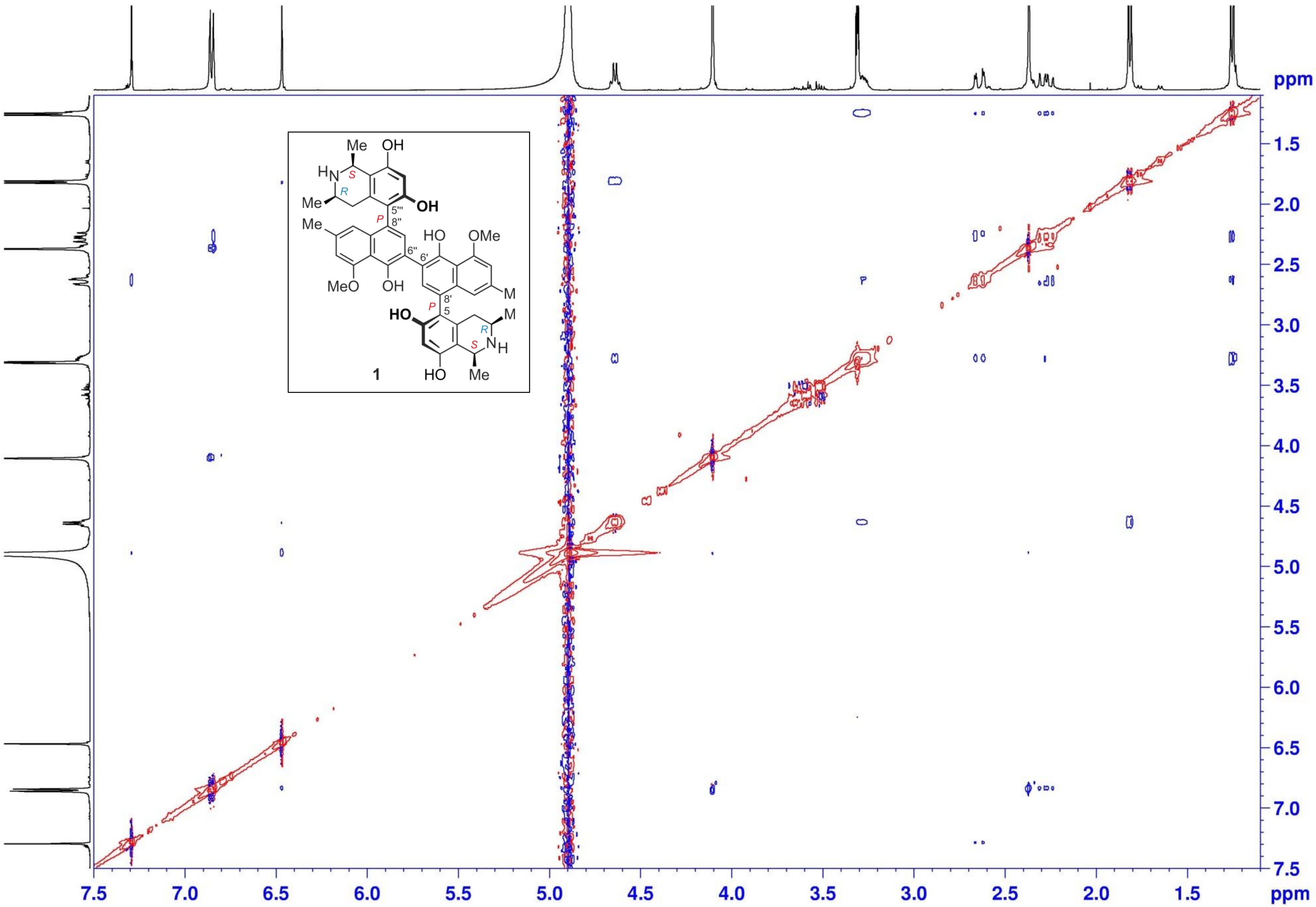

Fig. S7 NOESY NMR spectrum of compound **1** in methanol-d_4

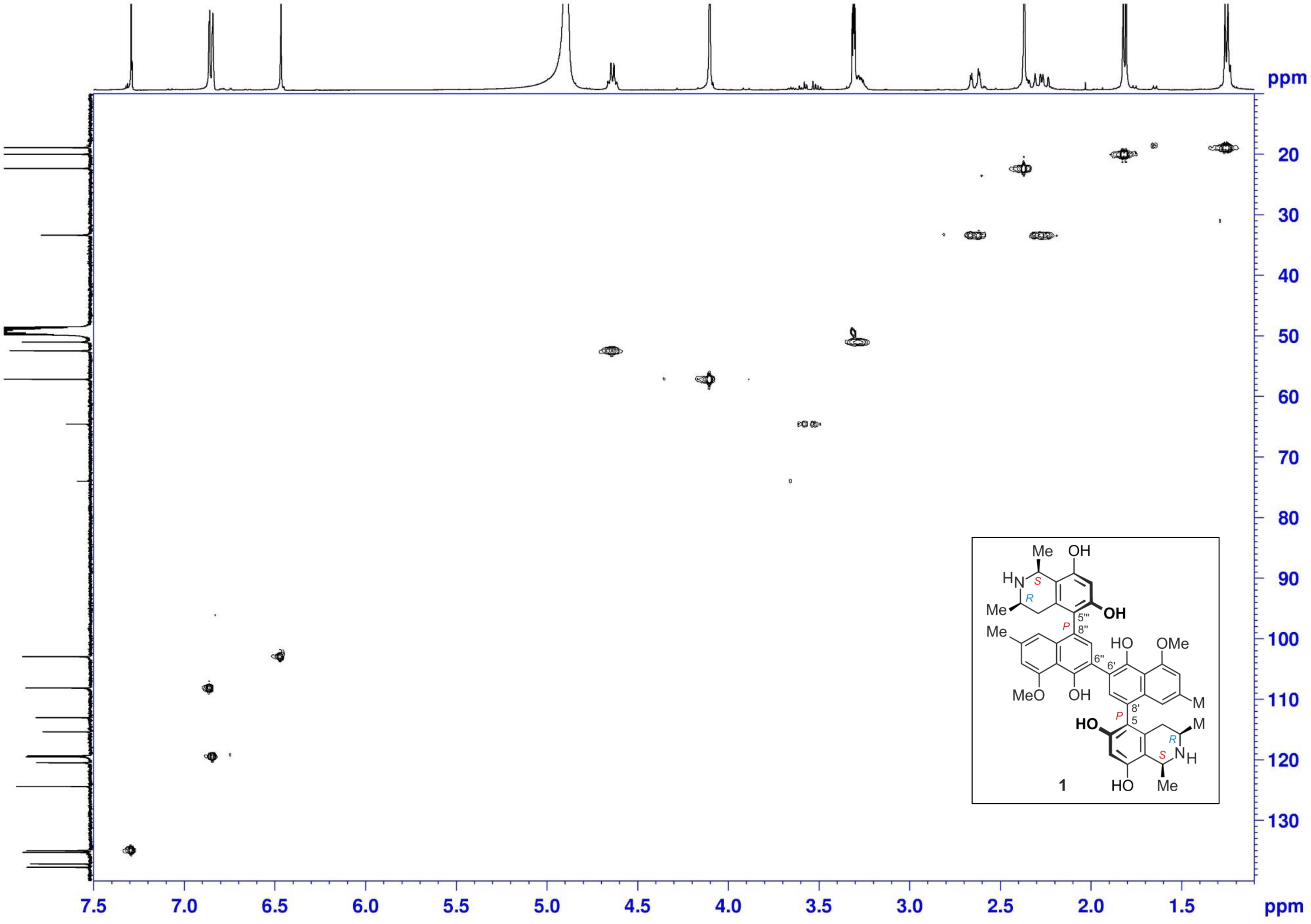

Fig. S8 HSQC NMR spectrum of compound **1** in methanol-d_4

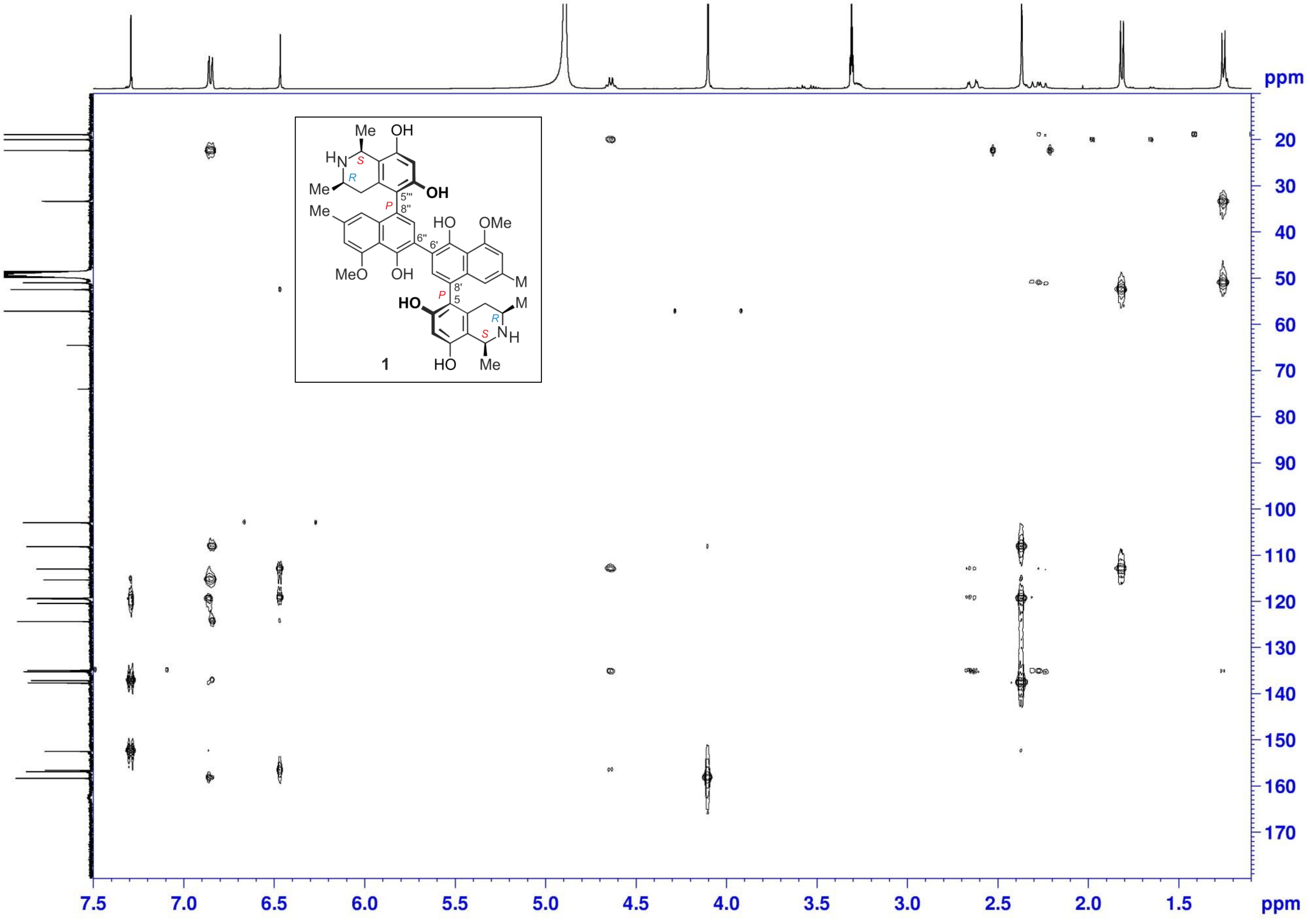

Fig. S9 HMBC NMR spectrum of compound **1** in methanol-d_4

Bruker Daltonics DataAnalysis 3.3

Mass Spectrum Molecular Formula Report

Analysis Info

Analysis Name	D:\Data\Spektren2015\2015_2603_WUE.d	Acquisition Date	14.12.2015 13:25:13
Method	esi_tune_pos_wide.m	Operator	Administrator
Comment	Blaise Kimbadi Lombe<br>BO3-H2O-4<br>8 pmol/µl in MeOH	Instrument	micrOTOF 88

Acquisition Parameter

Source Type	ESI	Ion Polarity	Positive	Set Corrector Fill	48 V
Scan Range	n/a	Capillary Exit	200.0 V	Set Pulsar Pull	804 V
Scan Begin	50 m/z	Hexapole RF	380.0 V	Set Pulsar Push	807 V
Scan End	3500 m/z	Skimmer 1	50.0 V	Set Reflector	1700 V
		Hexapole 1	23.0 V	Set Flight Tube	8600 V
				Set Detector TOF	2140 V

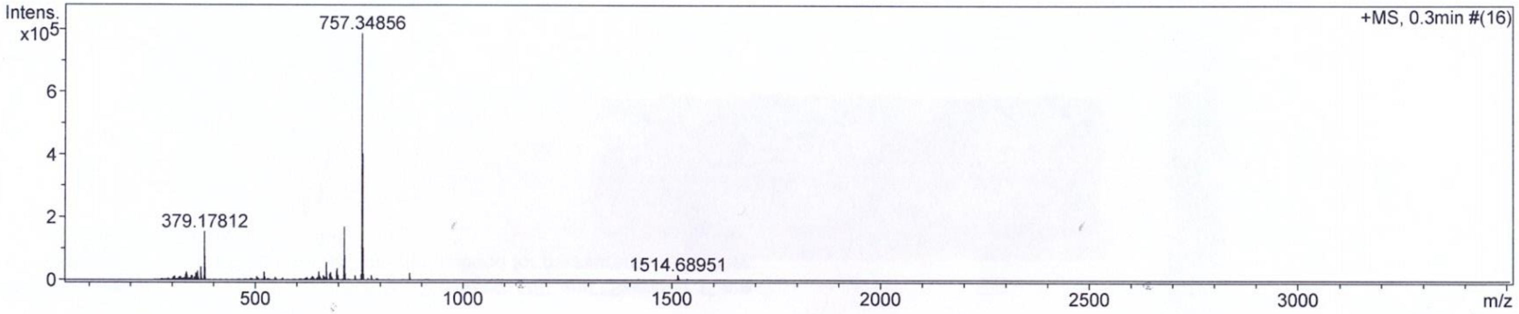

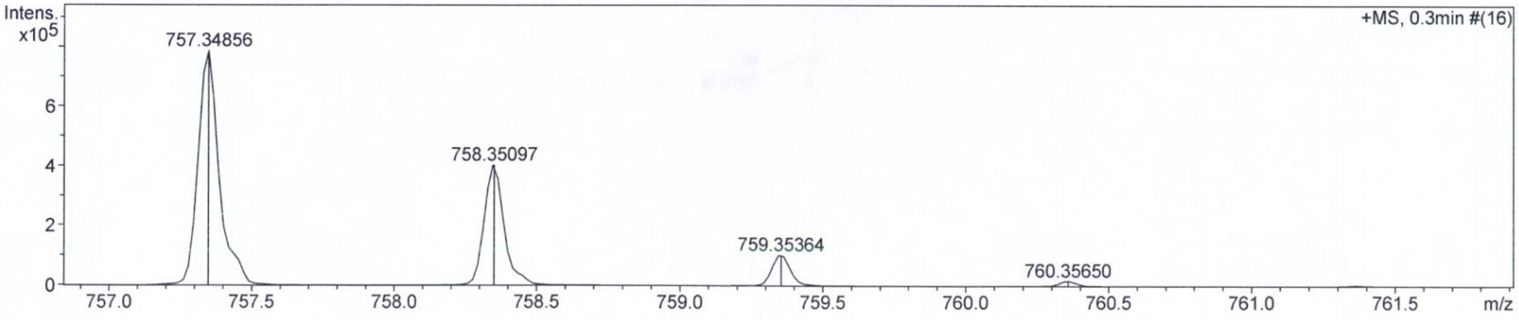

Sum Formula	Sigma	m/z	Err [ppm]	Mean Err [ppm]	rdb	N Rule	e⁻
C 46 H 49 N 2 O 8	0.01	757.34834	-0.29	0.26	23.50	ok	even

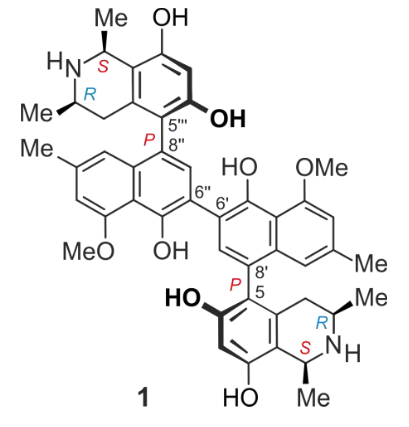

Fig. S10 HRESIMS spectrum of compound **1**

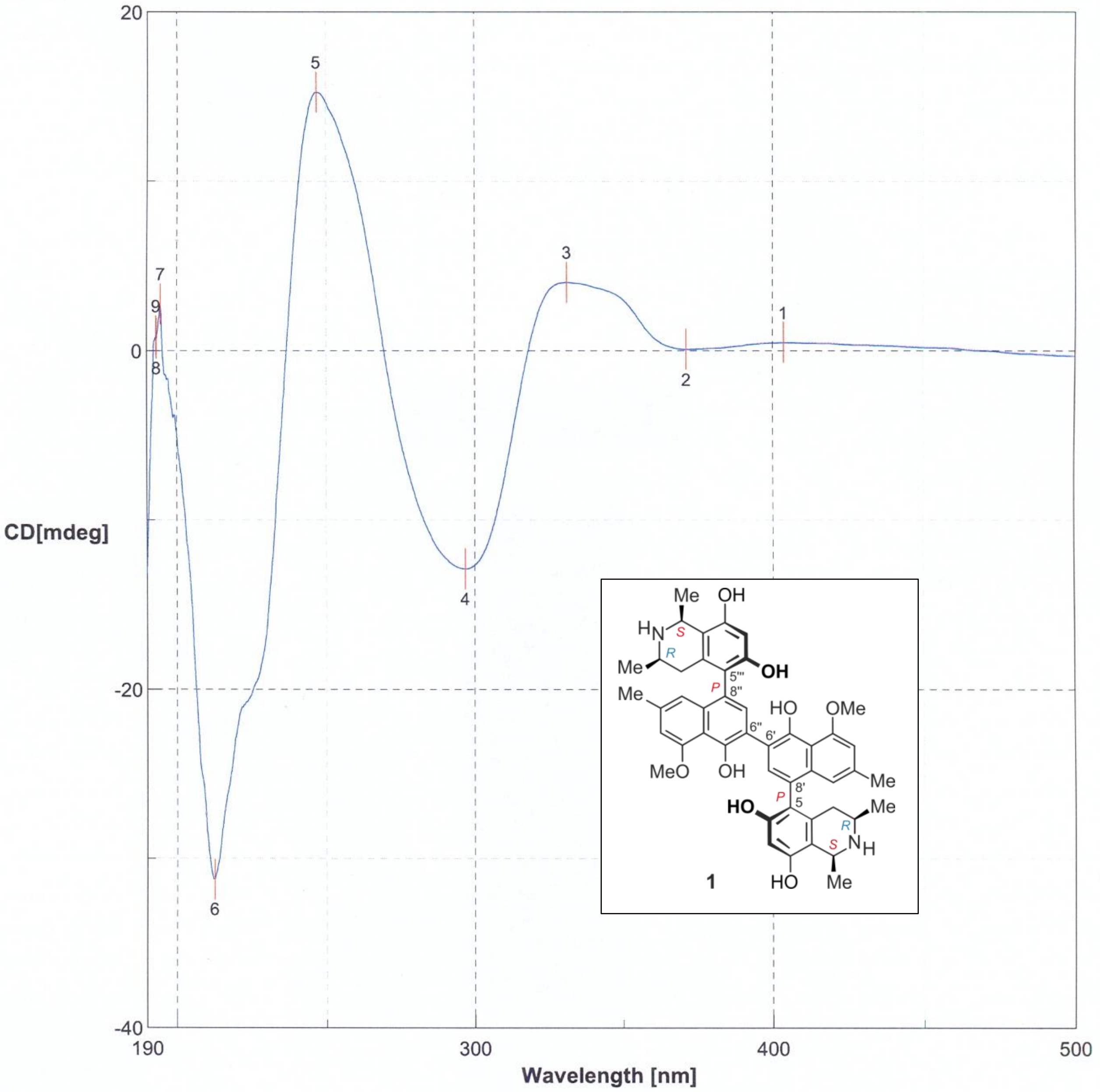

Date/Time	22.12.2015 16:21
Operator	Kimbadi
File Name	BO3-H2O-4#005.jws
Sample Name	BO3-H2O-4
Comment	offline

No.	nm	CD[mdeg]	No.	nm	CD[mdeg]	No.	nm	CD[mdeg]
1	403.6	0.467302	2	371.2	0.0578394	3	331.1	4.01686
4	296.8	-12.8825	5	246.7	15.2553	6	212.4	-31.2292
7	194.6	2.7439	8	193.2	0.727451	9	193.1	0.852667

Fig. S11 CD spectrum of compound **1** in methanol

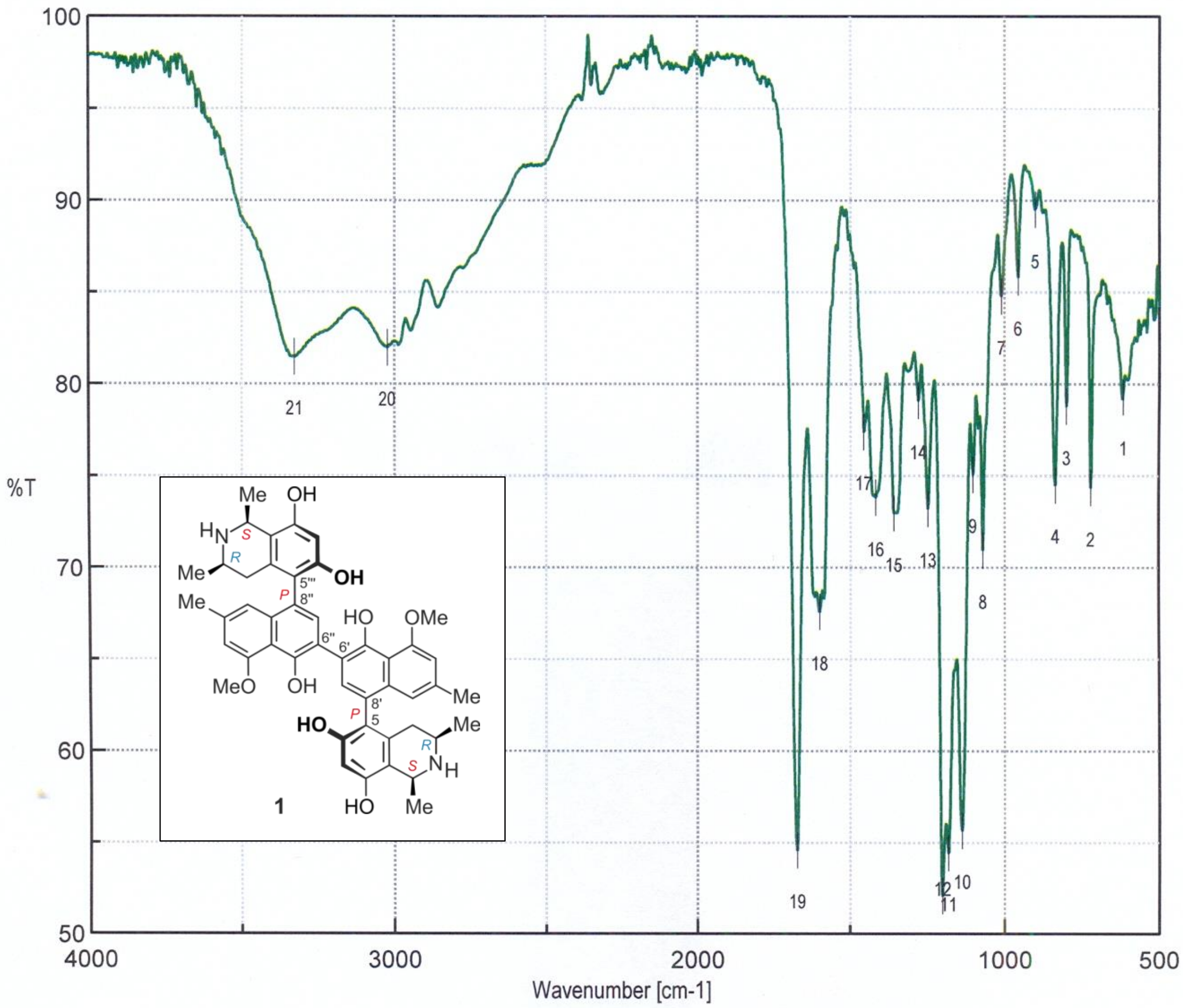

Results of Peak Find

No.	Position	Intensity	No.	Position	Intensity
1	617.109	79.2789	2	722.211	74.3236
3	799.35	78.7506	4	835.99	74.4534
5	901.558	89.4596	6	956.52	85.8008
7	1009.55	84.7557	8	1071.26	70.8984
9	1102.12	75.0456	10	1137.8	55.6355
11	1181.19	54.3946	12	1200.47	52.0827
13	1248.68	73.1612	14	1279.54	79.0713
15	1358.6	72.9091	16	1418.39	73.7761
17	1455.99	77.3635	18	1599.66	67.5549
19	1671.98	54.5506	20	3022.87	81.9626
21	3328.53	81.4322			

[Comments]

Sample name	BO3-H2O-4
Comment	drop(MeOH)
User	Kimbadi
Division	AK Bringmann
Company	Uni Würzburg

[Measurement Information]

Model Name	FT/IR-4600typeA
Serial Number	D063461786
Accessory	ATR PRO ONE
Accessory S/N	A070661809
Incident angle	45 deg
Measurement Date	20.12.2017 14:11
Light Source	Standard
Detector	TGS
Accumulation	16
Resolution	4 cm-1
Zero Filling	On
Apodization	Cosine
Gain	Auto (4)
Aperture	Auto (7.1 mm)
Scanning Speed	Auto (2 mm/sec)
Filter	Auto (30000 Hz)

Fig. S12 IR spectrum of compound **1**

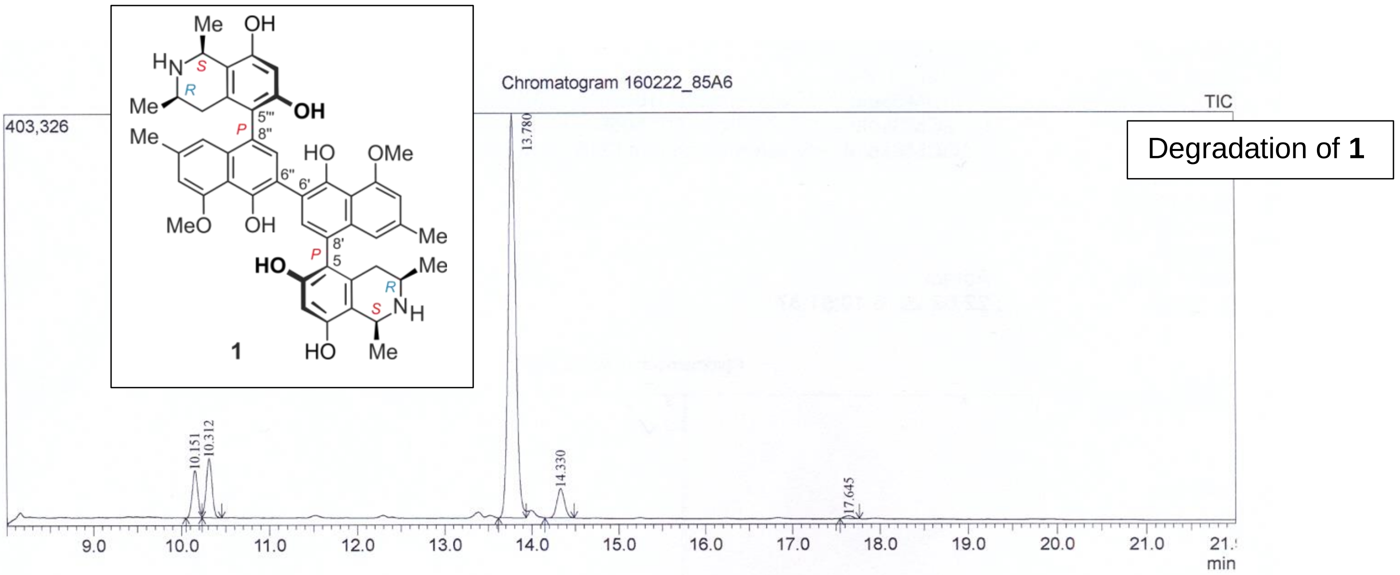

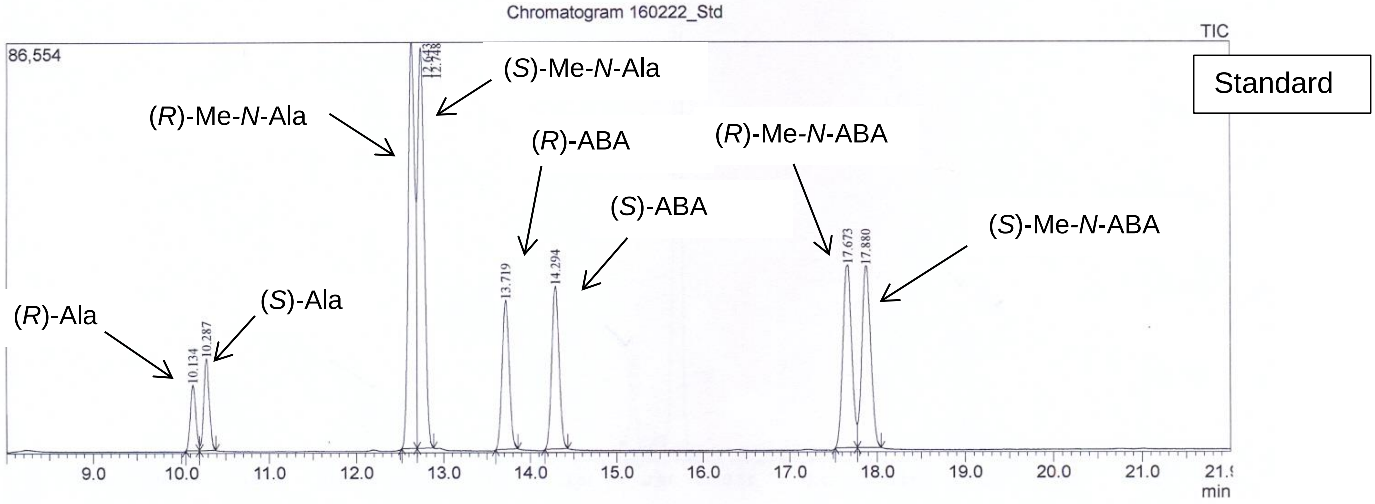

Where: Ala = alanine; Me-*N*-Ala = *N*-methylalanine;
ABA = 3-aminobyturic acid; Me-*N*-ABA = *N*-methyl 3-aminobyturic acid

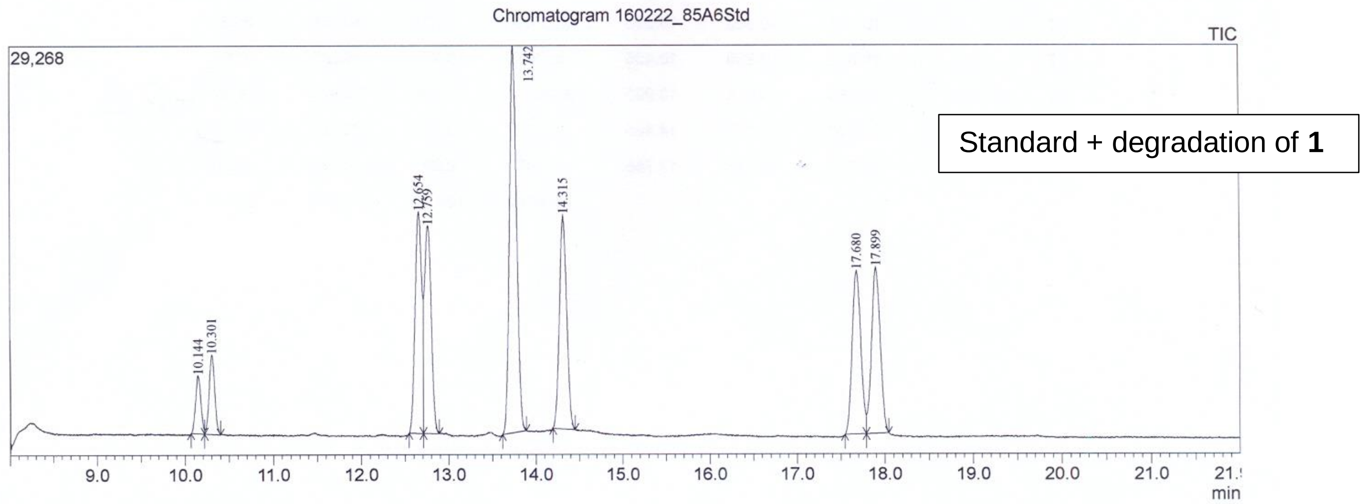

Fig. S13 Results of the oxidative degradation of compound **1**

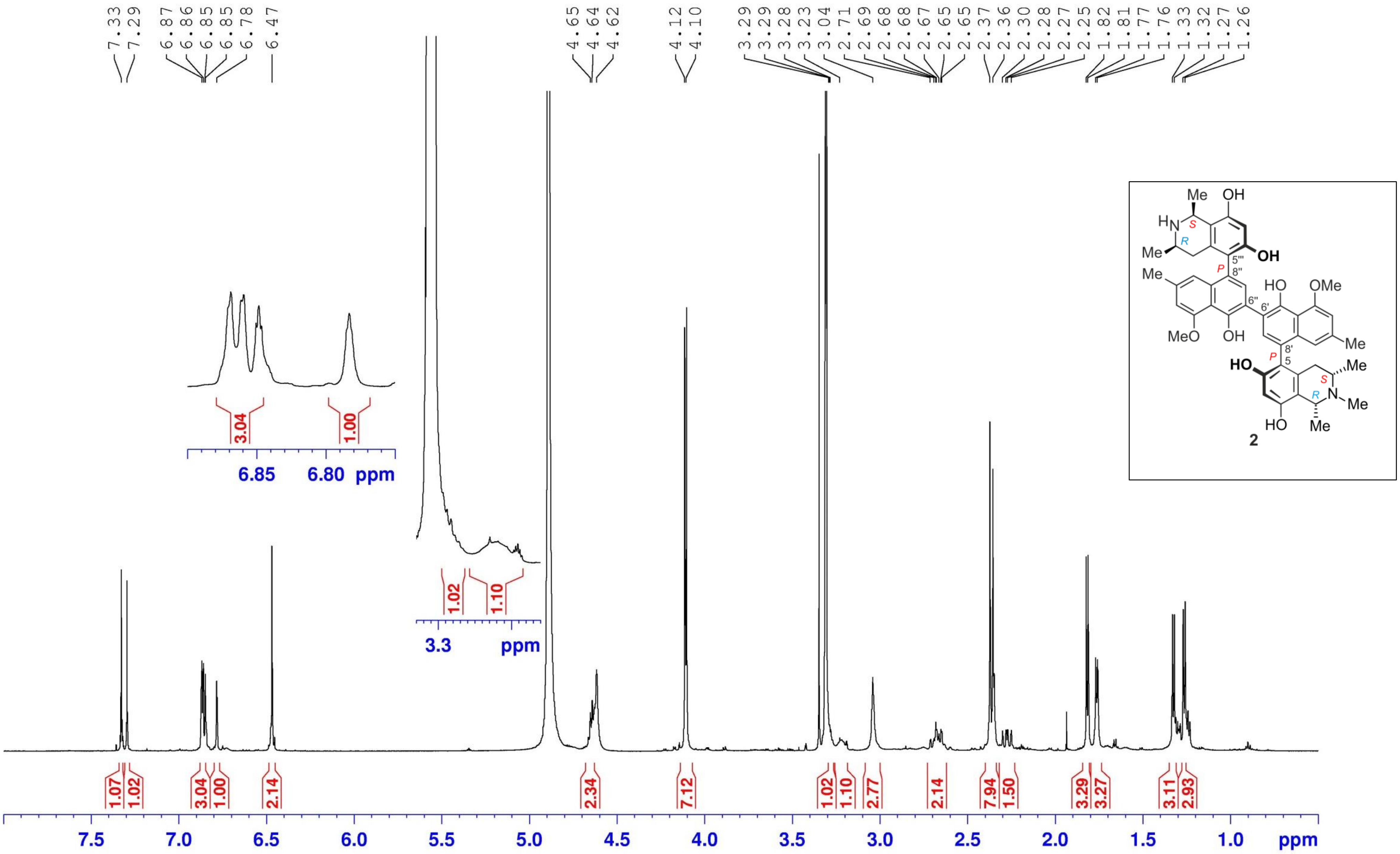

Fig. S14 ^{1}H NMR spectrum of compound **2** in methanol-d_4

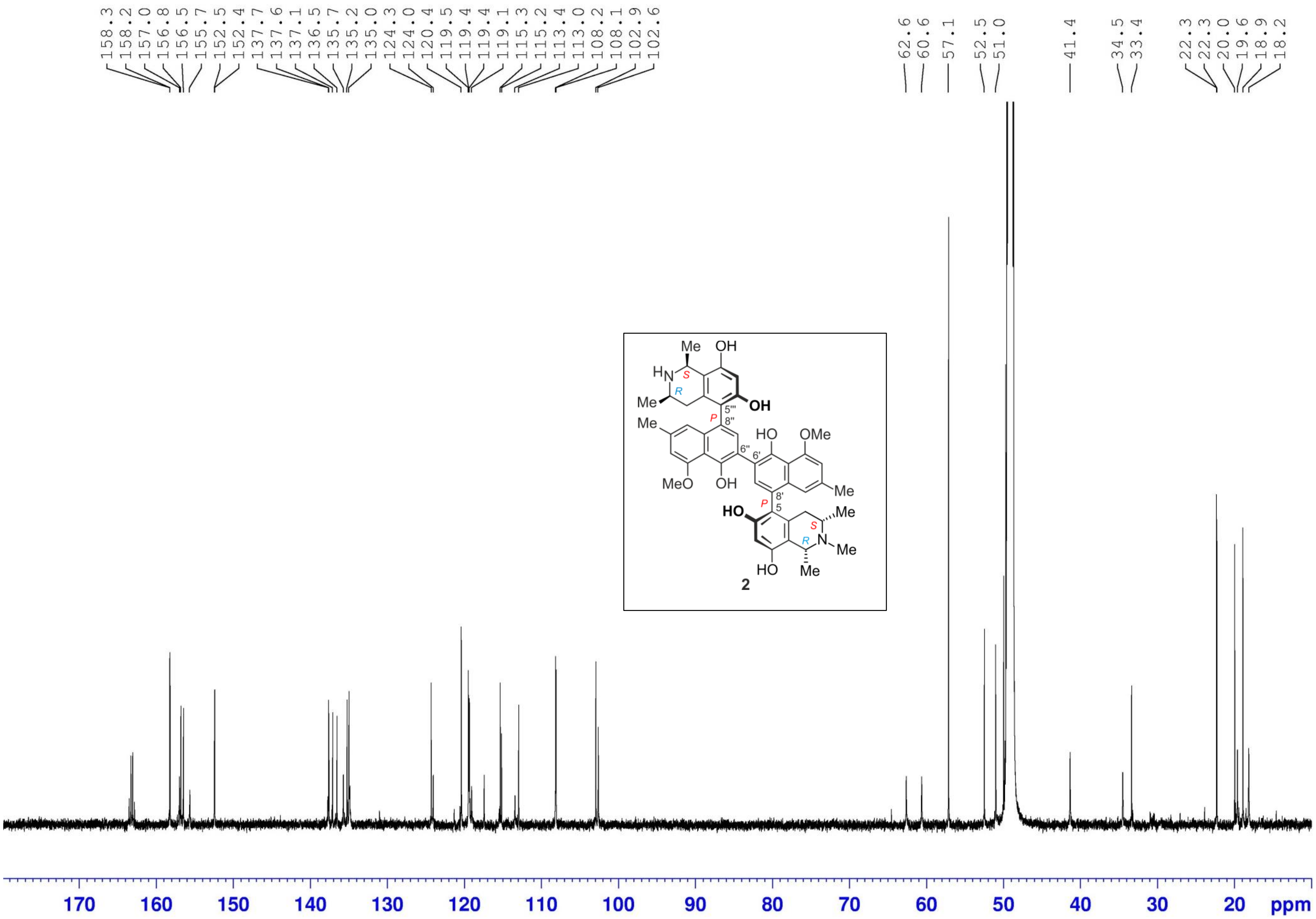

Fig. S15 ^{13}C NMR spectrum of compound **2** in methanol-d_4

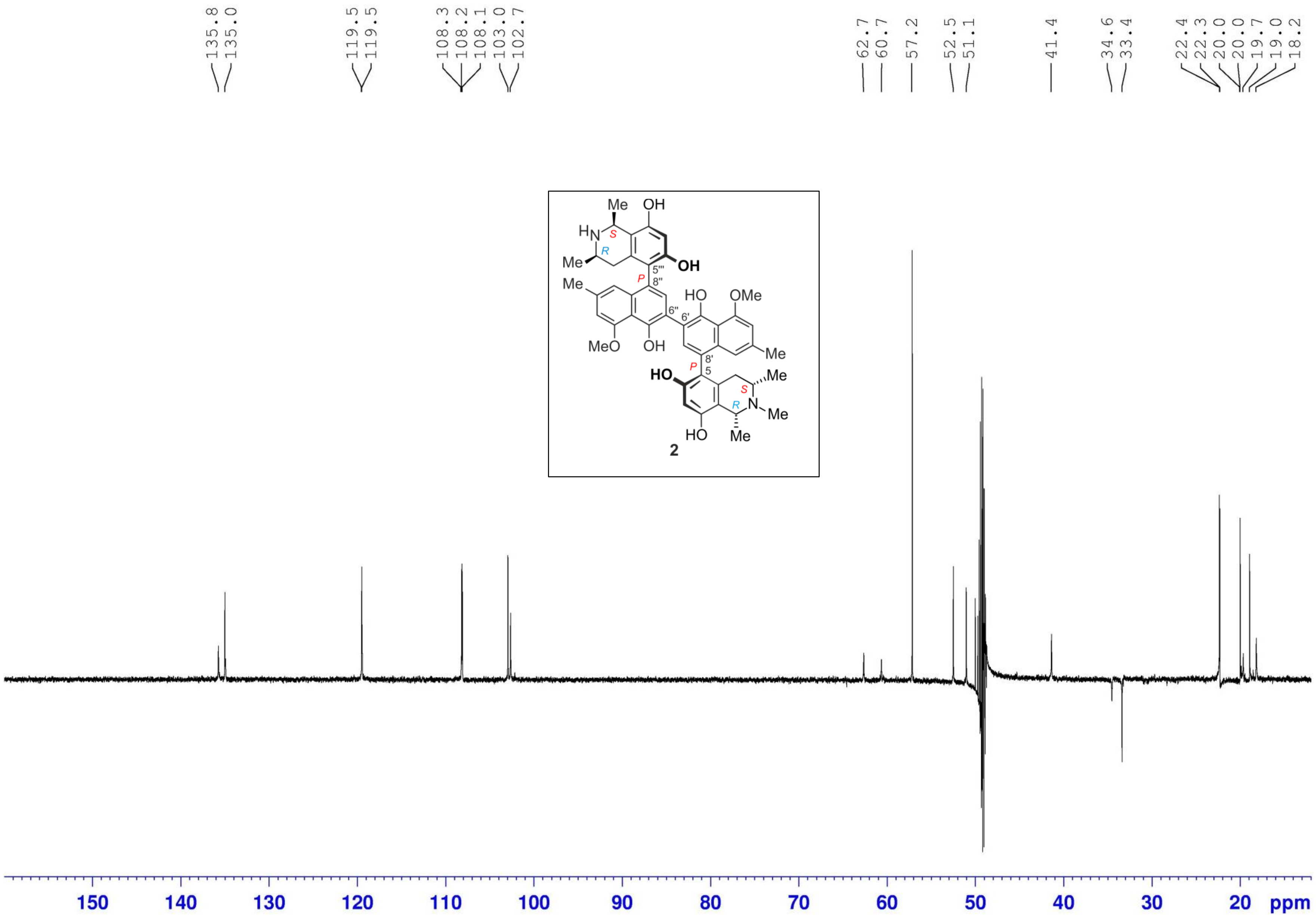

Fig. S16 DEPT-135 NMR spectrum of compound **2** in methanol-d_4

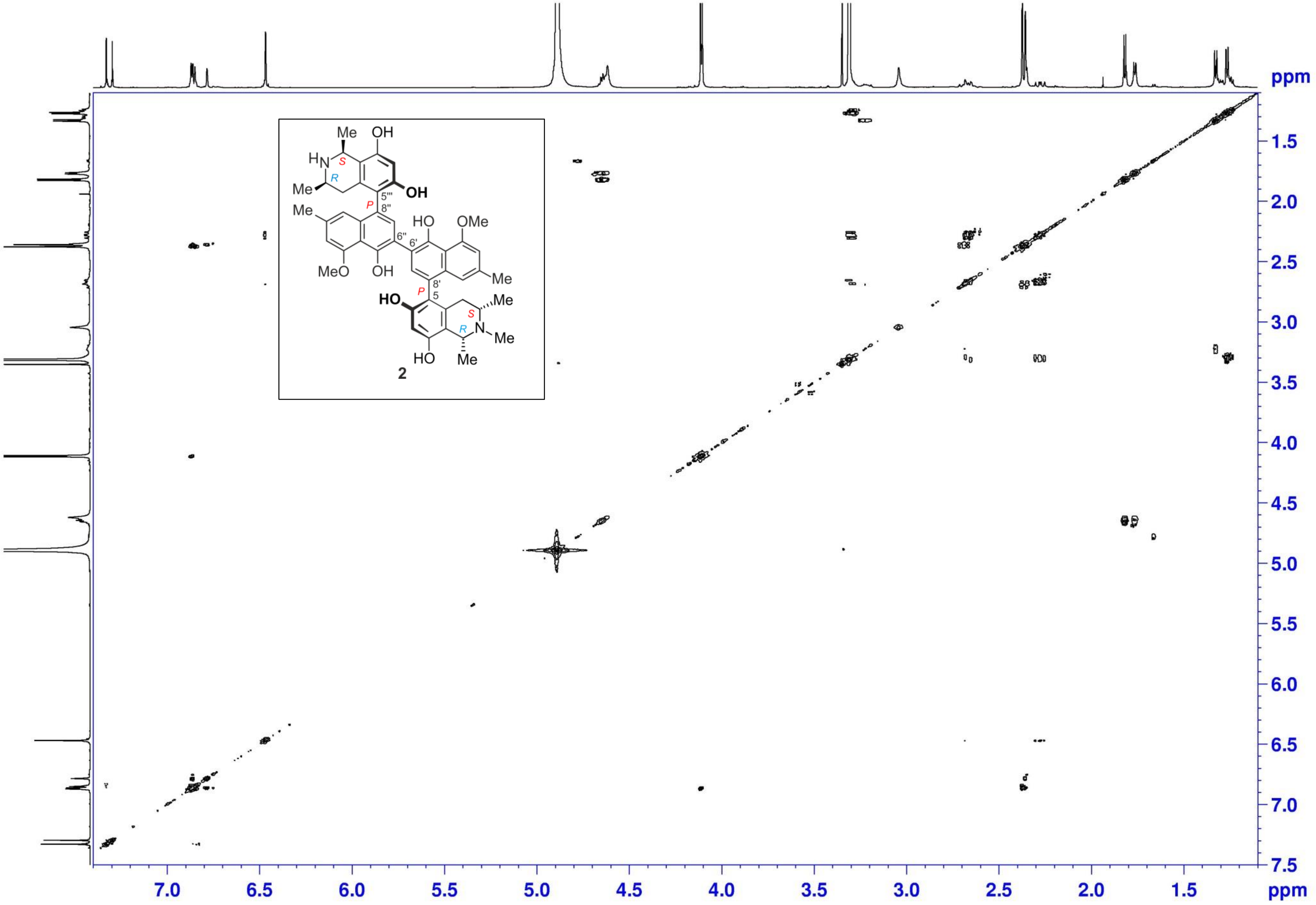

Fig. S17 COSY NMR spectrum of compound **2** in methanol-d_4

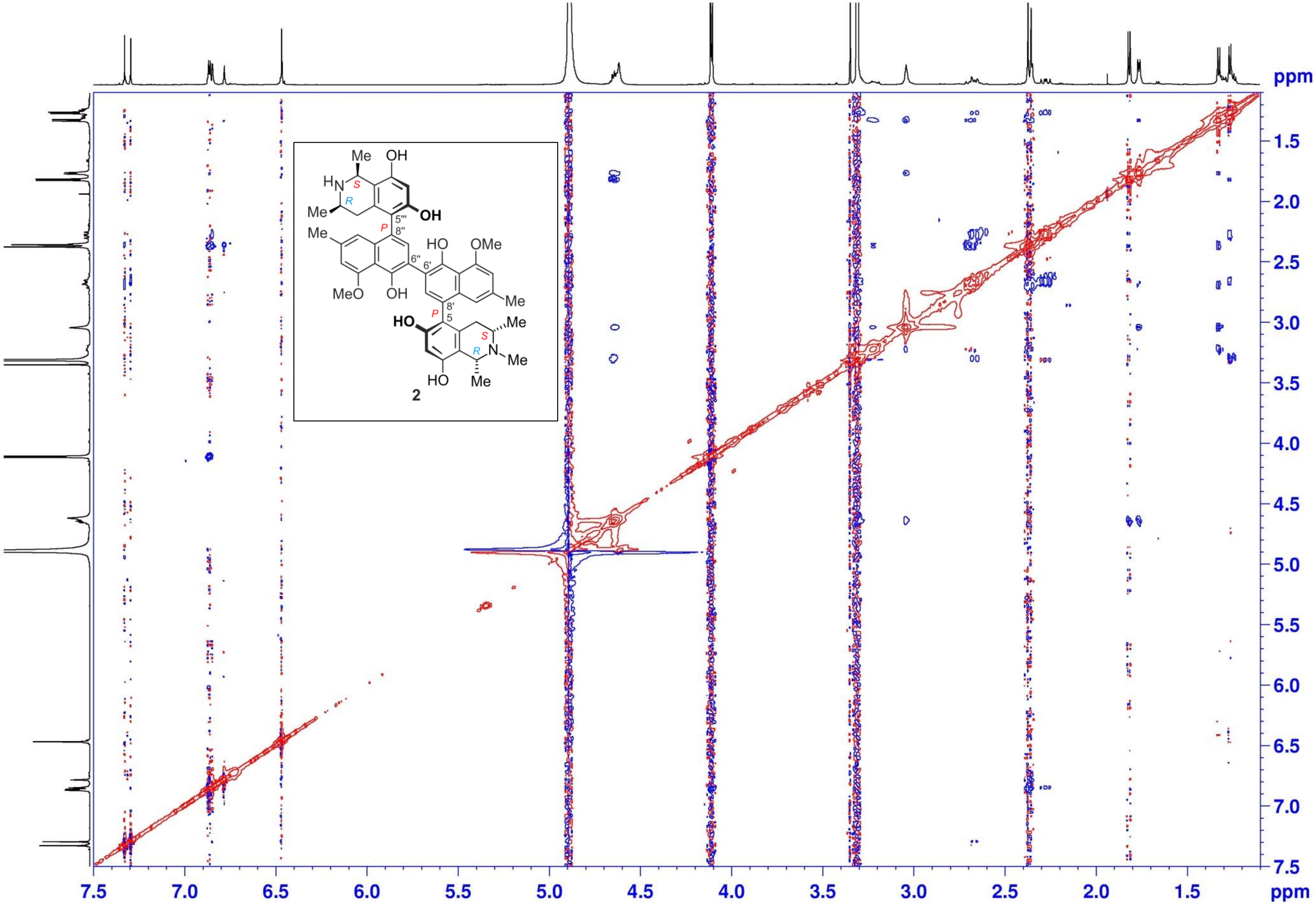

Fig. S18 ROESY NMR spectrum of compound **2** in methanol-d_4

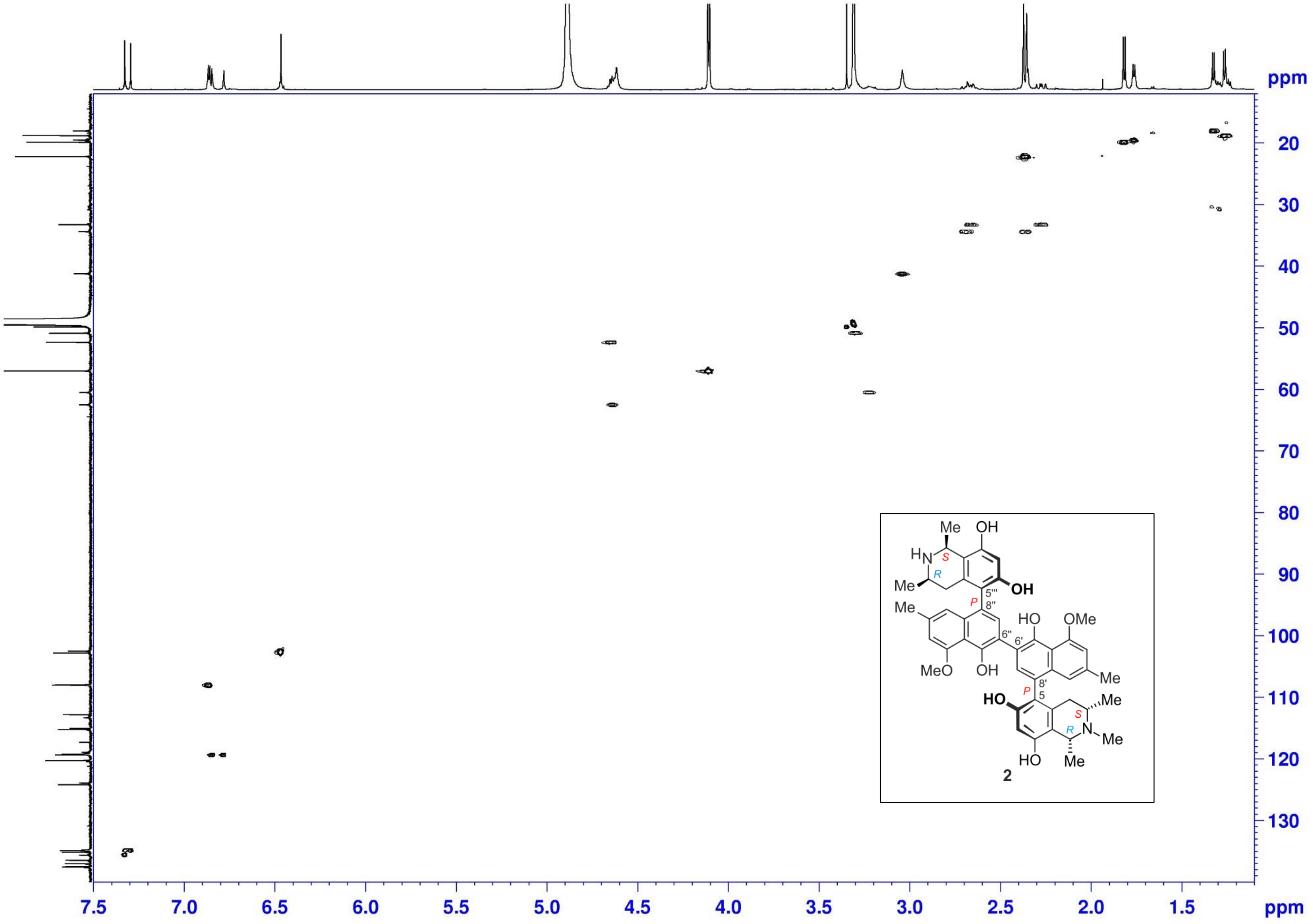

Fig. S19 HSQC NMR spectrum of compound **2** in methanol-d_4

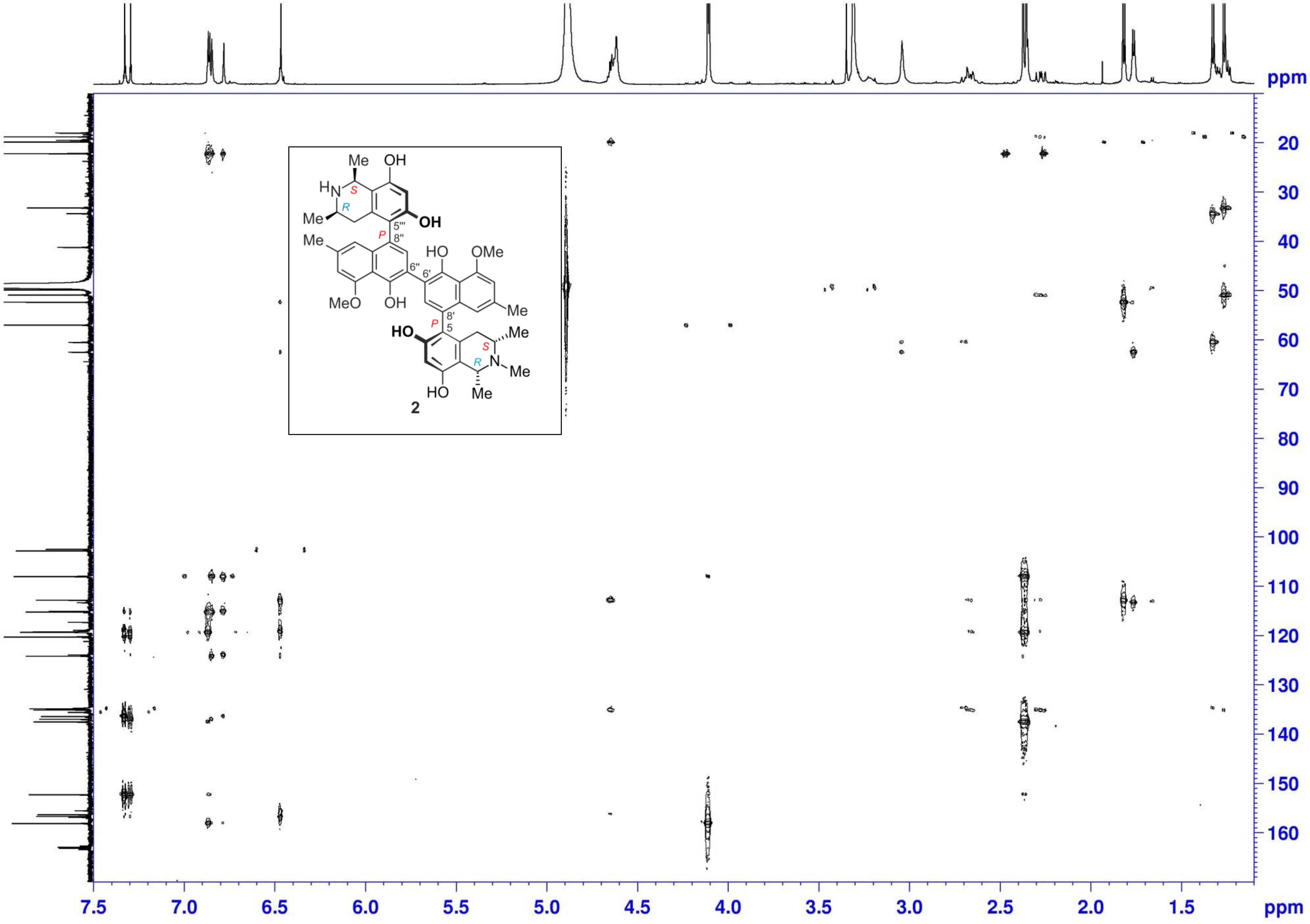

Fig. S20 HMBC NMR spectrum of compound **2** in methanol-d_4

Bruker Daltonics DataAnalysis 3.3

Mass Spectrum Molecular Formula Report

Analysis Info

Analysis Name	D:\Data\Spektren2015\2015_2655_BRI.d
Method	esi_tune_pos_wide.m
Comment	Blaise Kimbadi Lombe<br>B03-H2O-5<br>8 pmol/µl in MeOH
Acquisition Date	17.12.2015 14:43:32
Operator	Administrator
Instrument	micrOTOF 88

Acquisition Parameter

Source Type	ESI	Ion Polarity	Positive	Set Corrector Fill	48 V
Scan Range	n/a	Capillary Exit	280.0 V	Set Pulsar Pull	804 V
Scan Begin	50 m/z	Hexapole RF	480.0 V	Set Pulsar Push	807 V
Scan End	3500 m/z	Skimmer 1	50.0 V	Set Reflector	1700 V
		Hexapole 1	23.0 V	Set Flight Tube	8600 V
				Set Detector TOF	2240 V

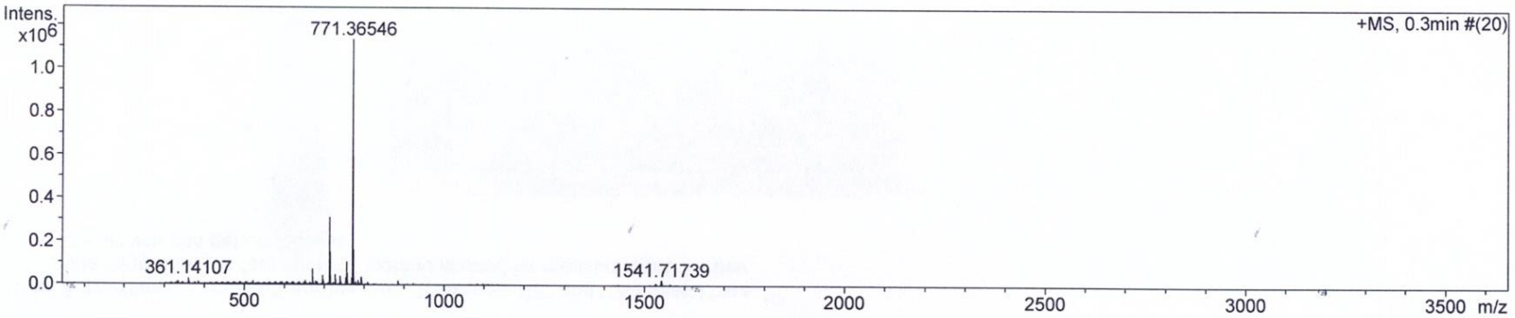

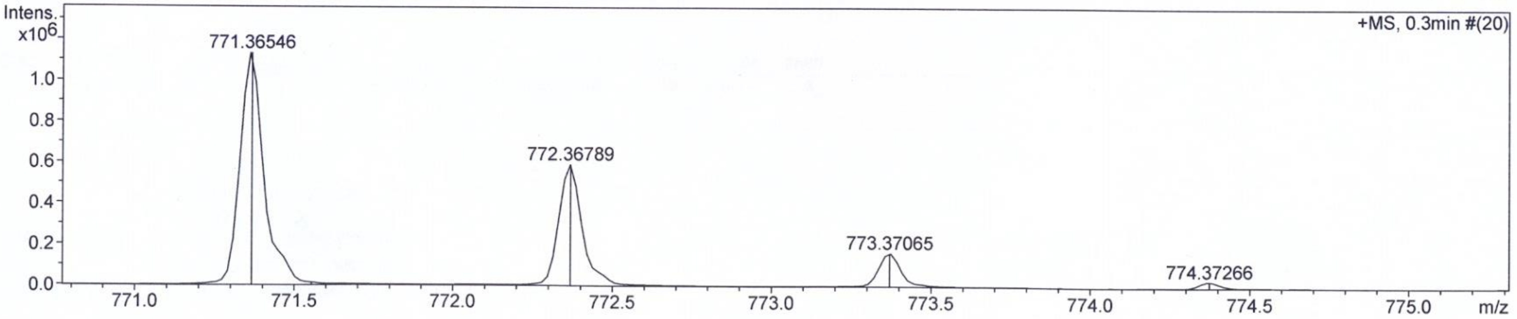

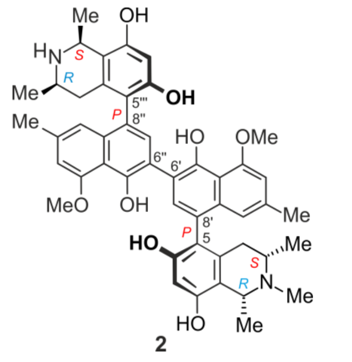

Sum Formula	Sigma	m/z	Err [ppm]	Mean Err [ppm]	rdb	N Rule	e⁻
C 47 H 51 N 2 O 8	0.01	771.36399	-1.90	-1.34	23.50	ok	even

Fig. S21 HRESIMS spectrum of compound **2**

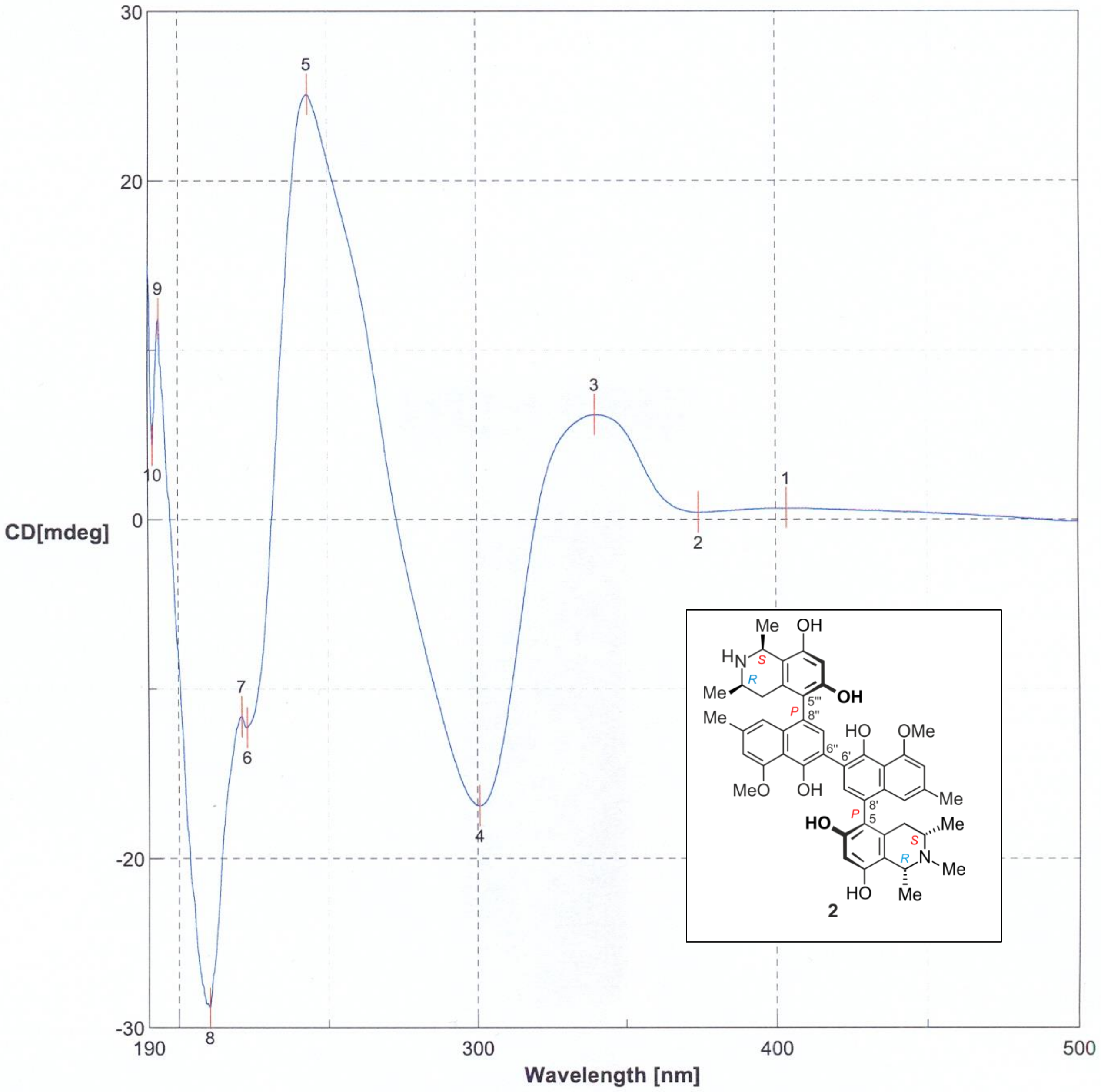

Date/Time	02.02.2016 15:40
Operator	Kimbadi
File Name	BO3-H2O-5#001.jws
Sample Name	BO3-H2O-5
Comment	offline

No.	nm	CD[mdeg]	No.	nm	CD[mdeg]	No.	nm	CD[mdeg]
1	403.5	0.655446	2	374.3	0.40599	3	339.7	6.18278
4	300.7	-16.9044	5	243.3	25.0888	6	222.9	-12.2832
7	221	-11.6263	8	210.2	-28.8258	9	193.4	11.8764
10	191.5	4.39749						

Fig. S22 CD spectrum of compound **2** in methanol

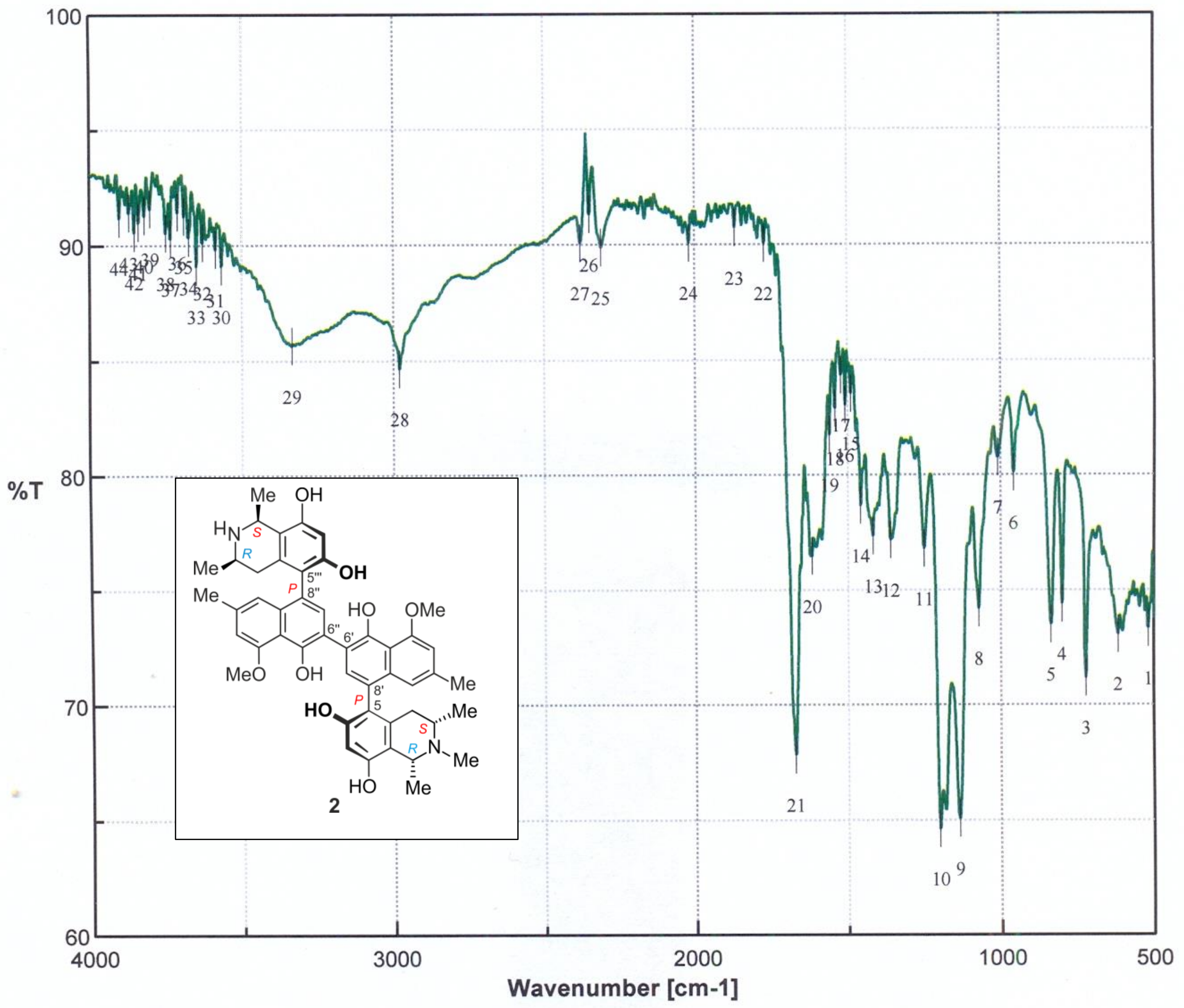

Results of Peak Find

No.	Position	Intensity	No.	Position	Intensity
1	517.793	73.3029	2	617.109	73.0542
3	722.211	71.1573	4	800.314	74.3847
5	836.955	73.472	6	957.484	80.0722
7	1010.52	80.7374	8	1072.23	74.1811
9	1136.83	65.0382	10	1200.47	64.5911
11	1250.61	76.7915	12	1359.57	77.1416
13	1418.39	77.3169	14	1456.96	78.6479
15	1488.78	83.5136	16	1507.1	83.0143
17	1520.6	84.3287	18	1540.85	82.8773
19	1559.17	81.7209	20	1617.02	76.4555
21	1671.98	67.8095	22	1771.3	90.0412
23	1868.68	90.719	24	2019.1	90.0556
25	2310.3	89.8648	26	2348.87	91.3138
27	2378.76	90.0698	28	2980.45	84.6094
29	3335.28	85.6191	30	3566.7	89.0788
31	3586.95	89.8216	32	3628.41	90.1109
33	3648.66	89.0997	34	3674.69	90.3252
35	3689.16	91.2494	36	3711.33	91.4469
37	3734.48	90.2764	38	3749.9	90.5335
39	3801.01	91.5978	40	3820.29	91.2772
41	3838.61	90.9839	42	3853.08	90.5647
43	3870.43	91.3998	44	3902.25	91.1807

[Comments]

Sample name	BO3-H2O-5
Comment	drop(MeOH)
User	Kimbadi
Division	AK Bringmann
Company	Uni Würzburg

[Measurement Information]

Model Name	FT/IR-4600typeA
Serial Number	D063461786
Accessory	ATR PRO ONE
Accessory S/N	A070661809
Incident angle	45 deg
Measurement Date	20.12.2017 15:03
Light Source	Standard
Detector	TGS
Accumulation	16
Resolution	4 cm−1
Zero Filling	On
Apodization	Cosine
Gain	Auto (4)
Aperture	Auto (7.1 mm)
Scanning Speed	Auto (2 mm/sec)
Filter	Auto (30000 Hz)

Fig. S23 IR spectrum of compound **2**

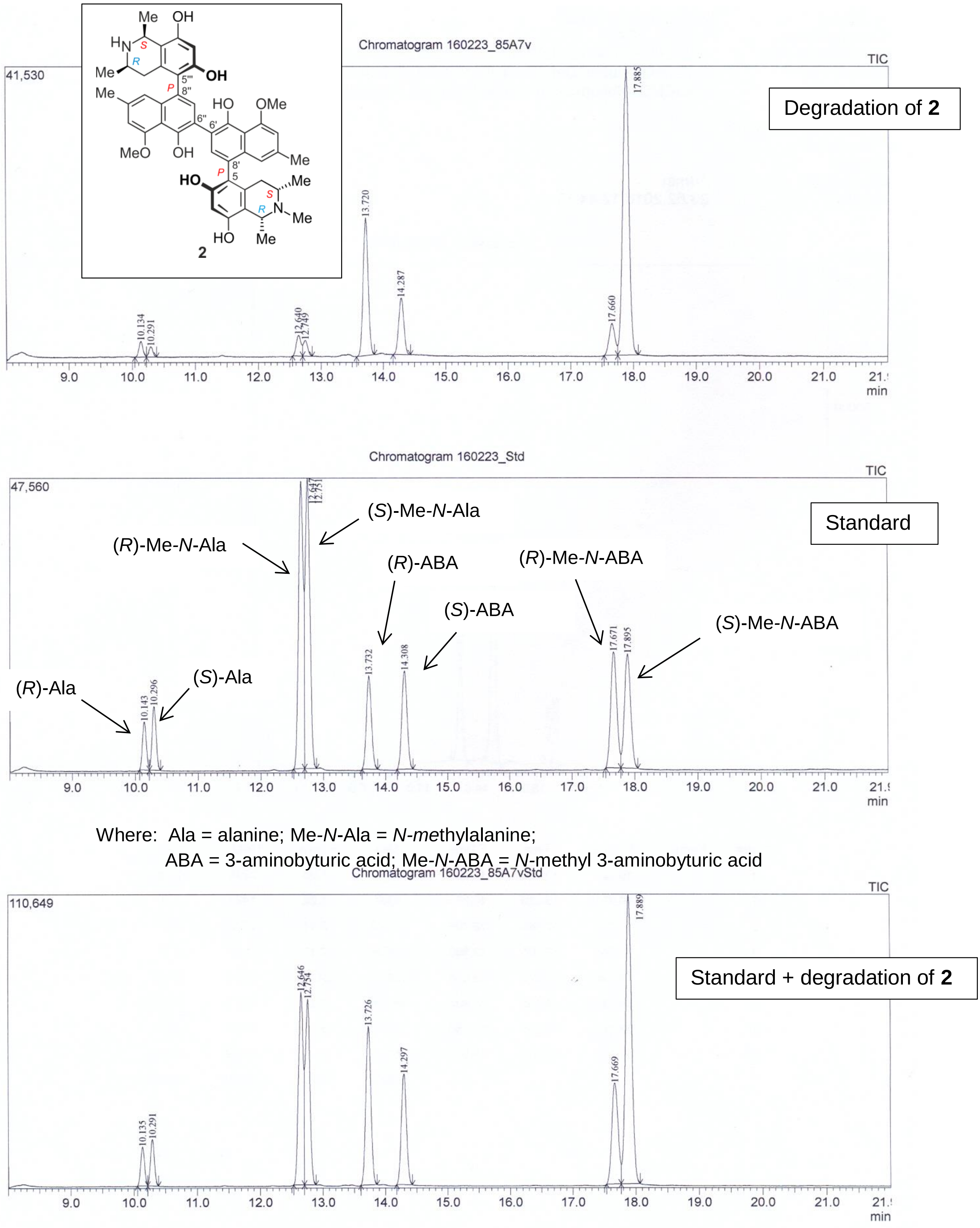

Fig. S24 Results of the oxidative degradation of compound **2**

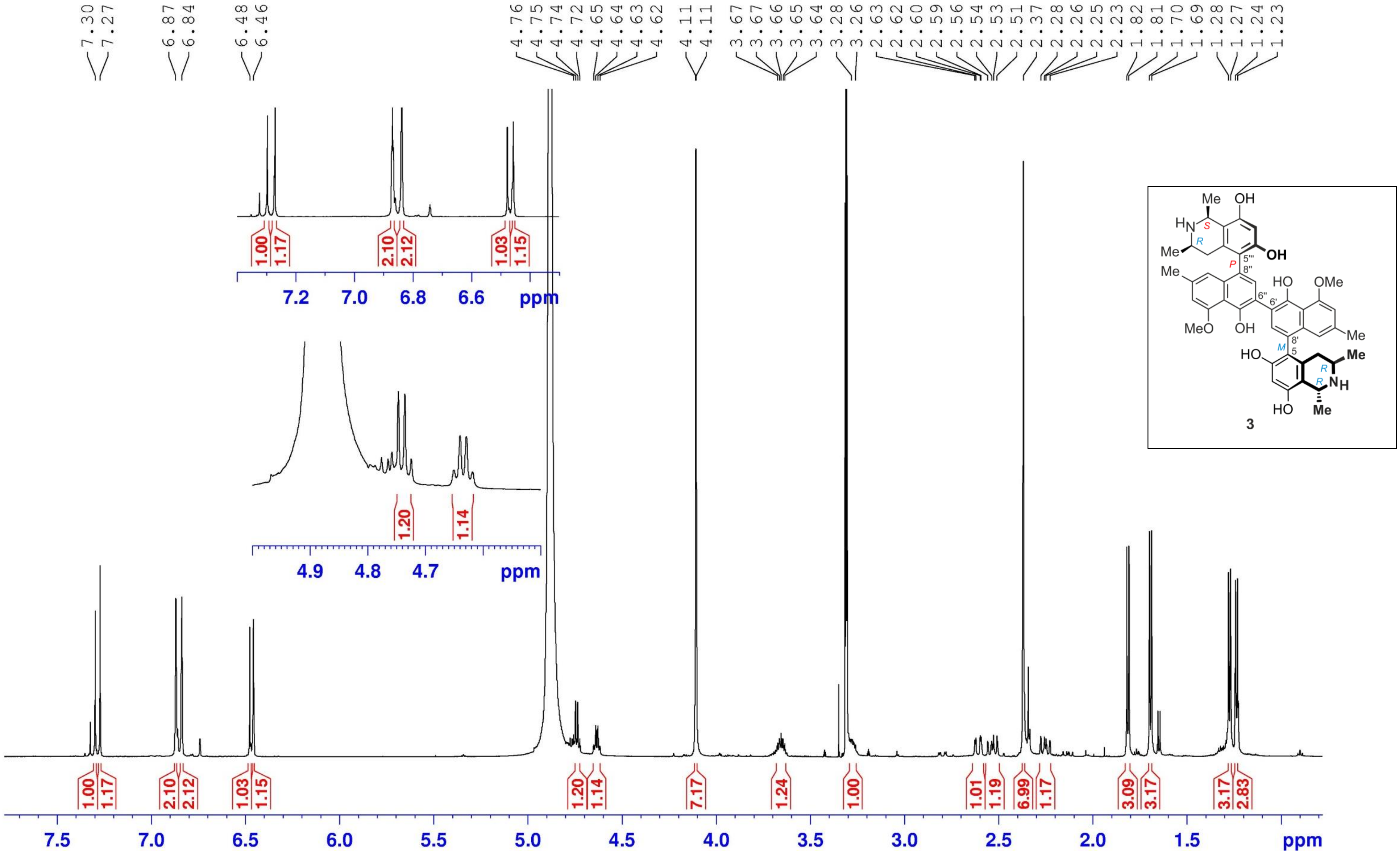

Fig. S25 ^{1}H NMR spectrum of compound **3** in methanol-d_4

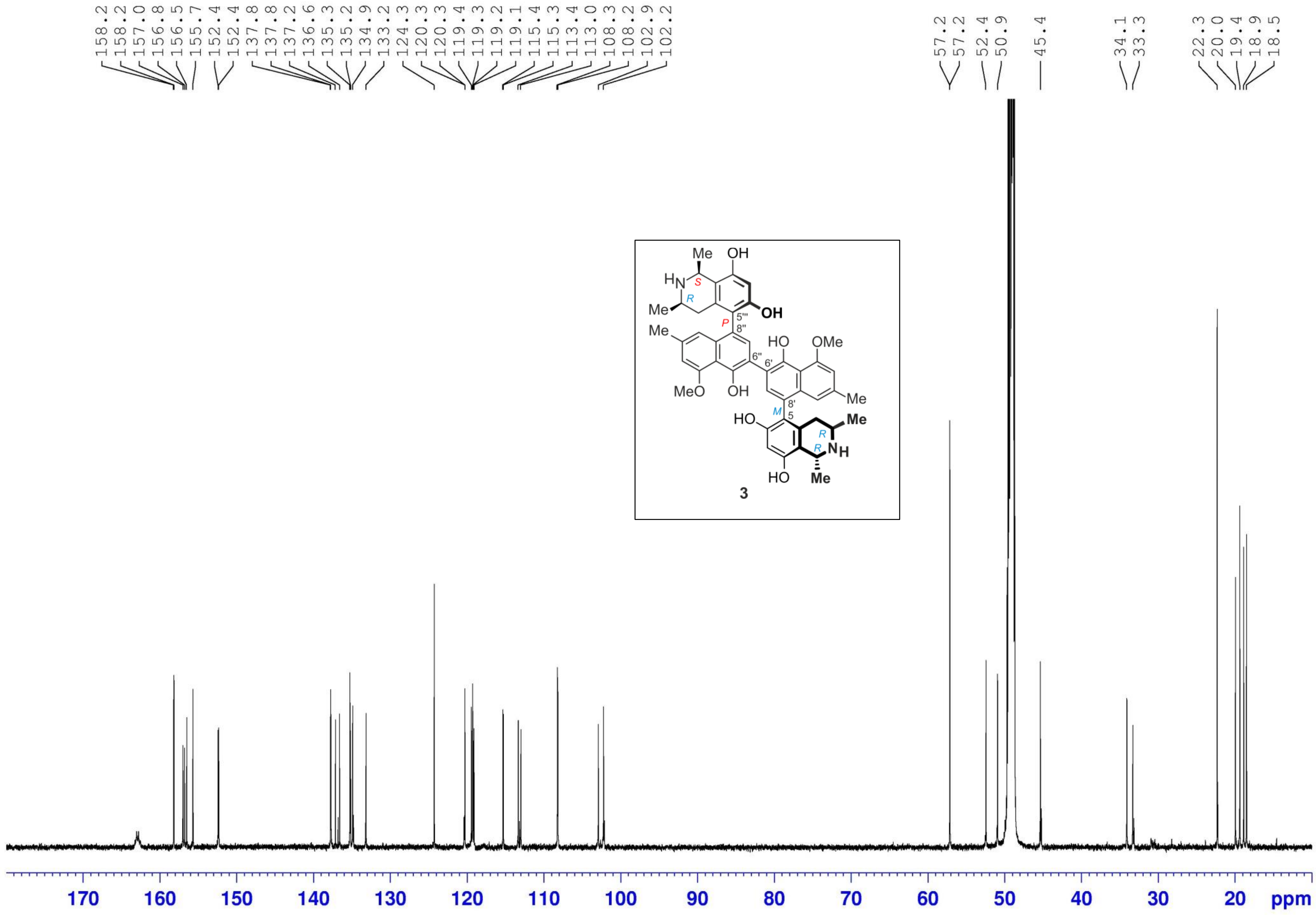

Fig. S26 ^{13}C NMR spectrum of compound **3** in methanol-d_4

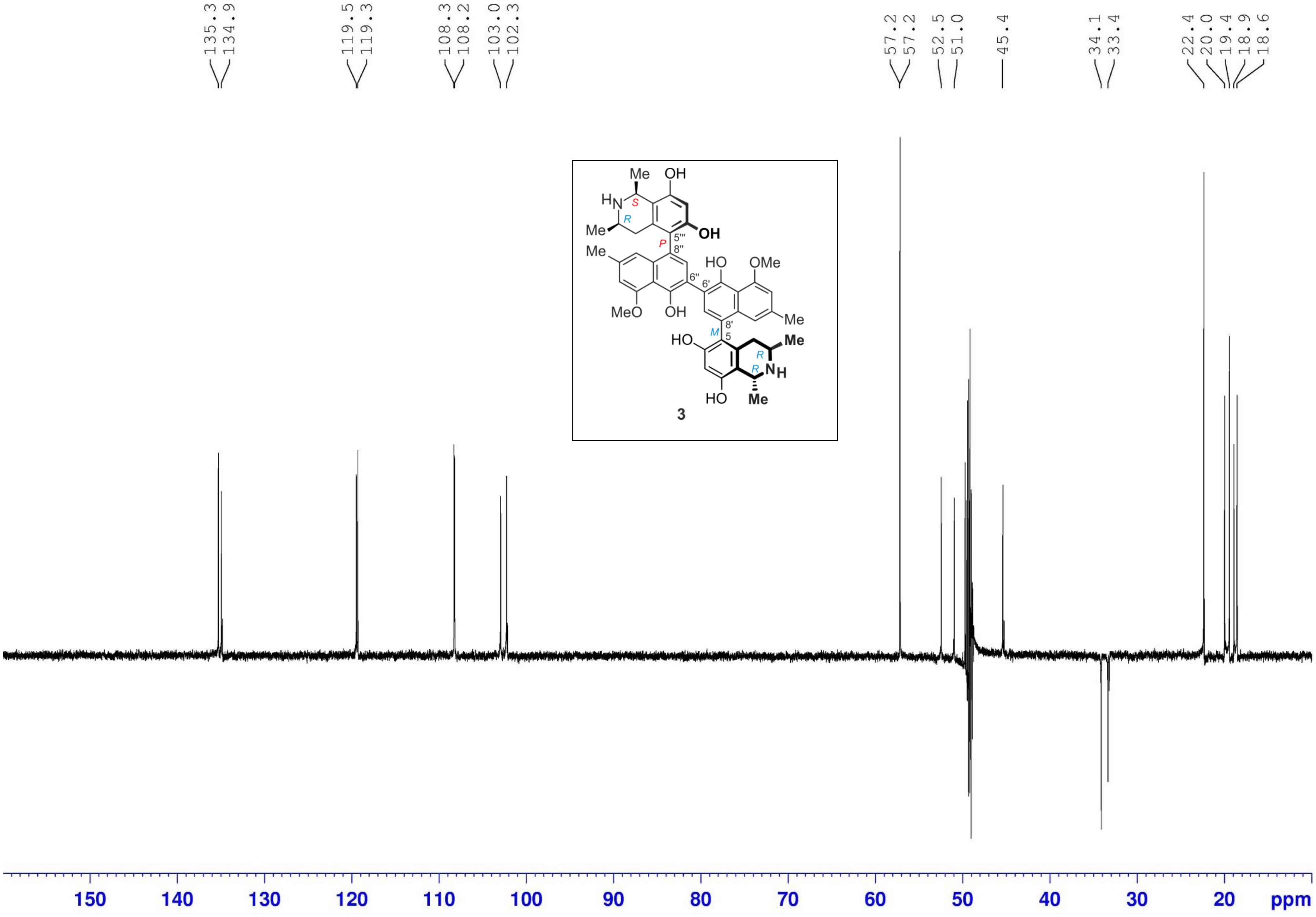

Fig. S27 DEPT-135 NMR spectrum of compound **3** in methanol-d_4

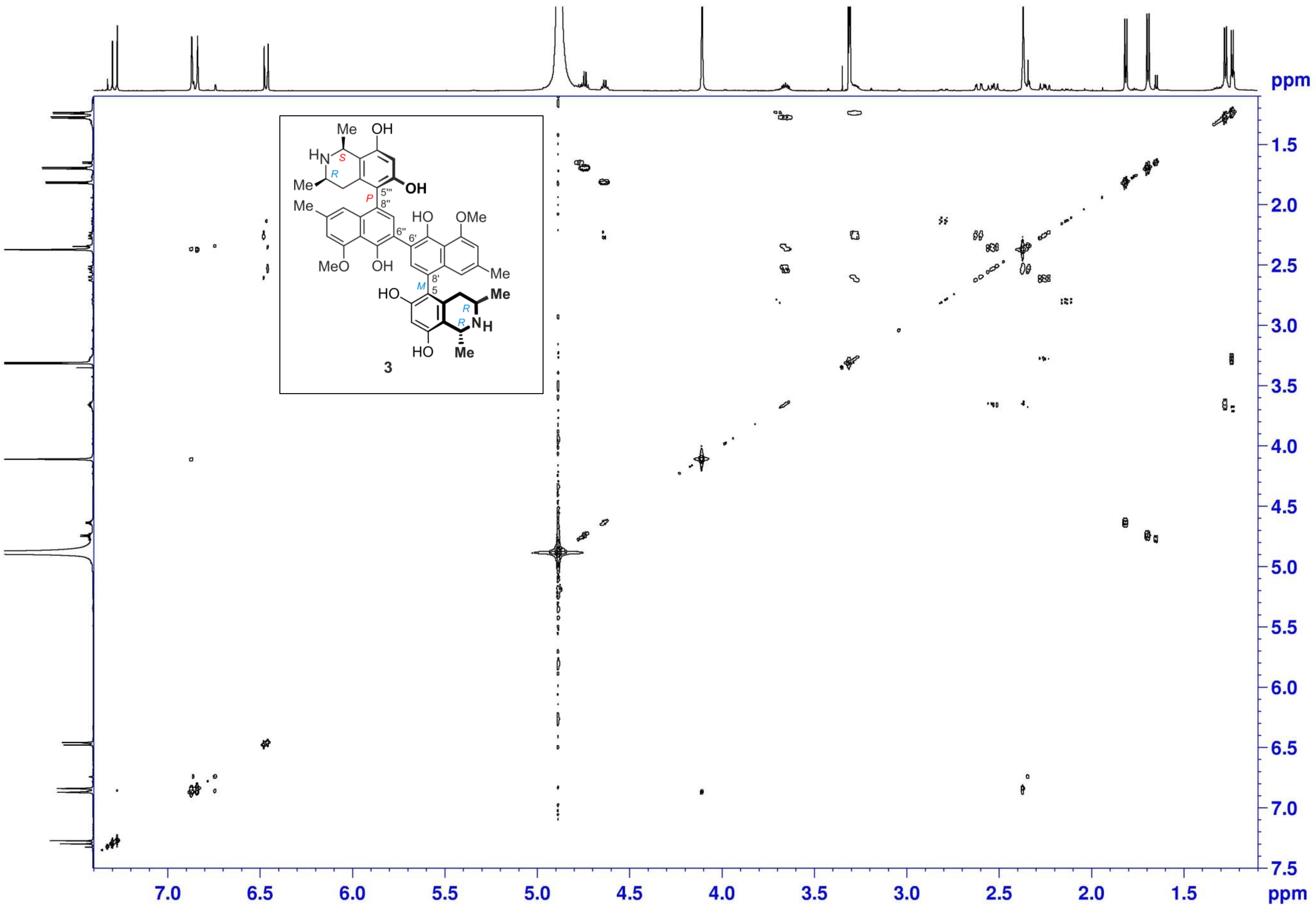

Fig. S28 COSY NMR spectrum of compound **3** in methanol-d_4

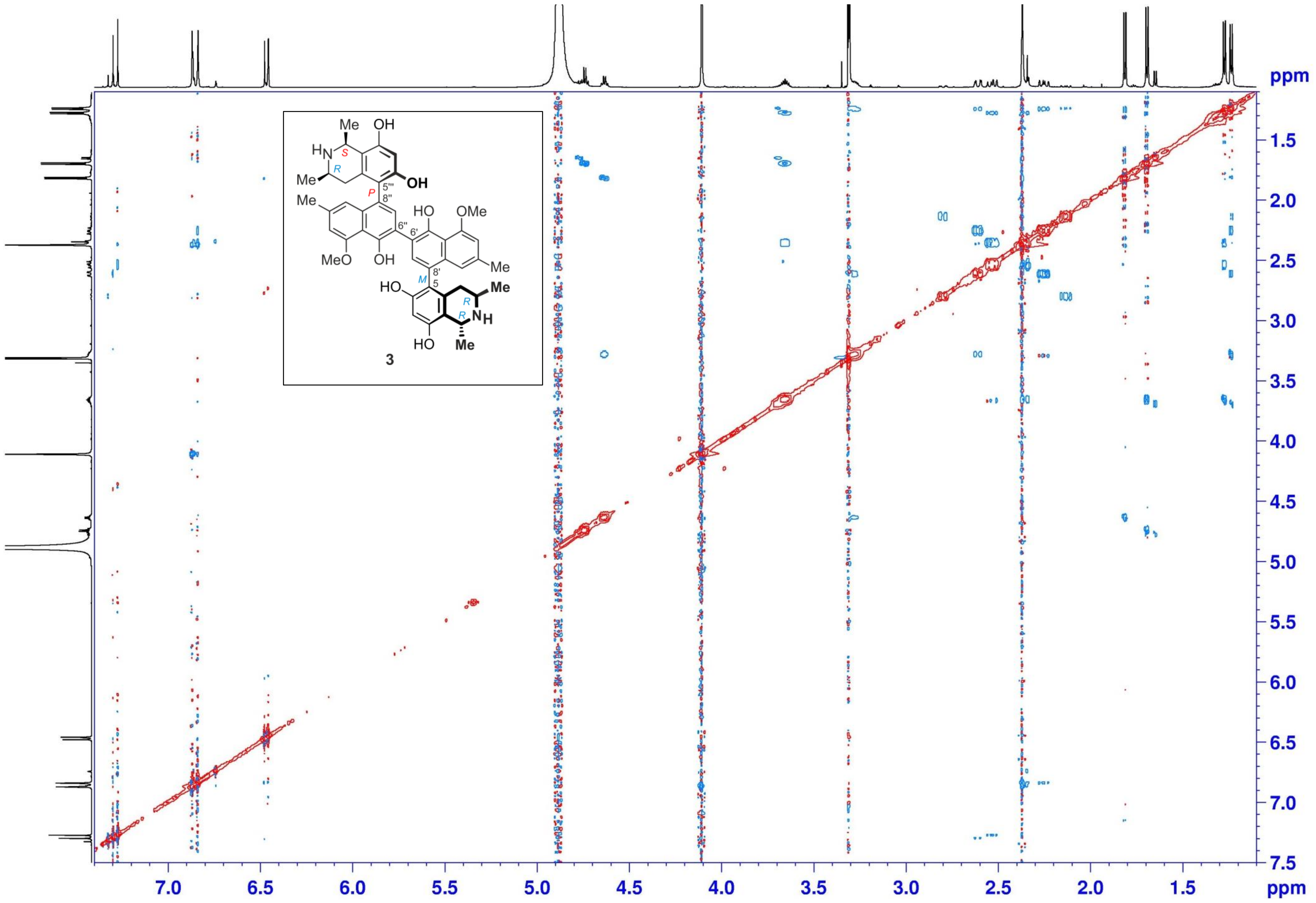

Fig. S29 ROESY NMR spectrum of compound **3** in methanol-d_4

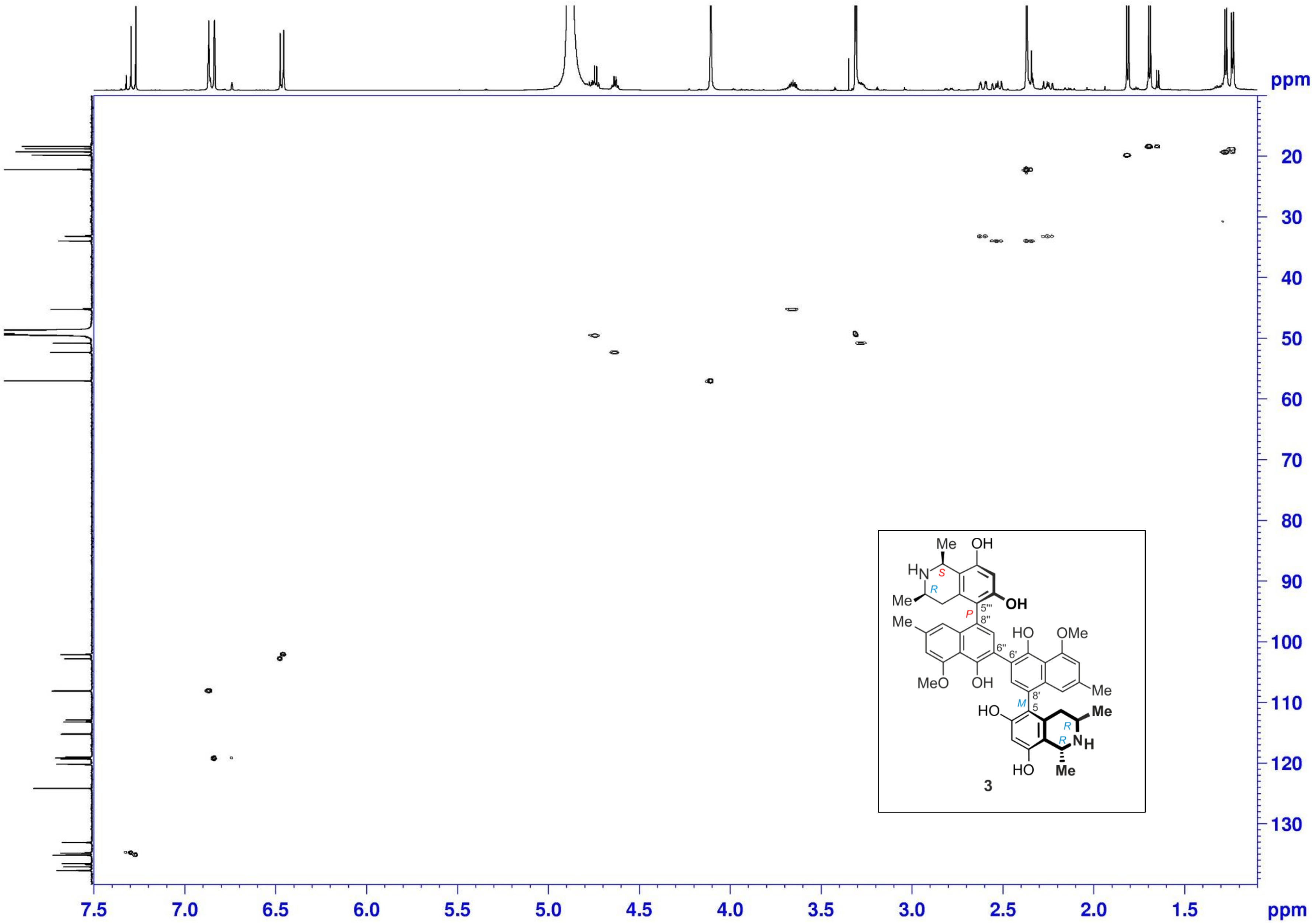

Fig. S30 HSQC NMR spectrum of compound **3** in methanol-d_4

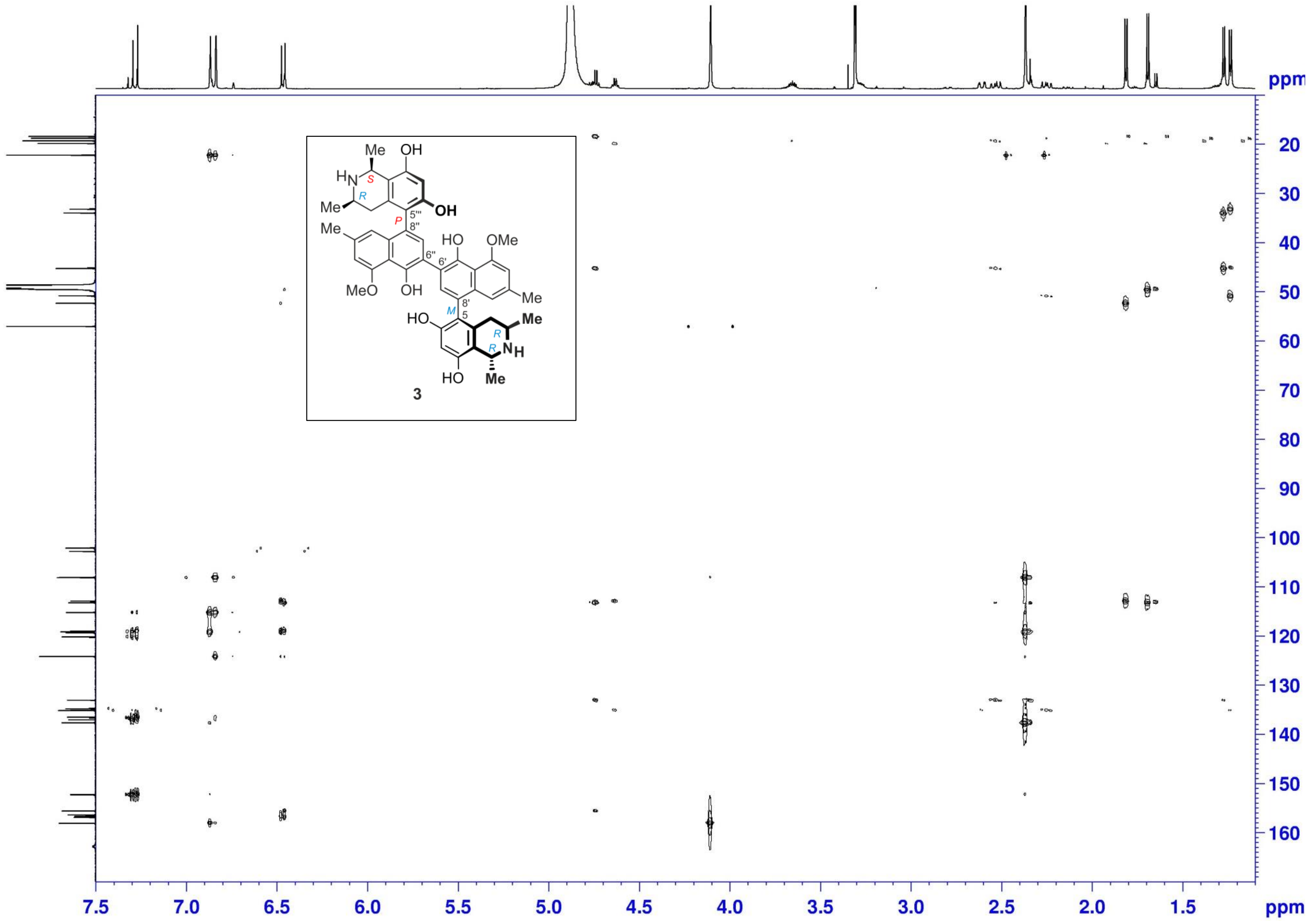

Fig. S31 HMBC NMR spectrum of compound **3** in methanol-d_4

Bruker Daltonics DataAnalysis 3.3

Mass Spectrum Molecular Formula Report

Analysis Info

Analysis Name	D:\Data\Spektren2015\2015_2654_BRI.d	Acquisition Date	17.12.2015 14:30:04
Method	esi_tune_pos_wide.m	Operator	Administrator
Comment	Blaise Kimbadi Lombe<br>B03-H2O-6<br>8 pmol/µl in MeOH	Instrument	micrOTOF 88

Acquisition Parameter

Source Type	ESI	Ion Polarity	Positive	Set Corrector Fill	48 V
Scan Range	n/a	Capillary Exit	280.0 V	Set Pulsar Pull	804 V
Scan Begin	50 m/z	Hexapole RF	480.0 V	Set Pulsar Push	807 V
Scan End	3500 m/z	Skimmer 1	50.0 V	Set Reflector	1700 V
		Hexapole 1	23.0 V	Set Flight Tube	8600 V
				Set Detector TOF	2240 V

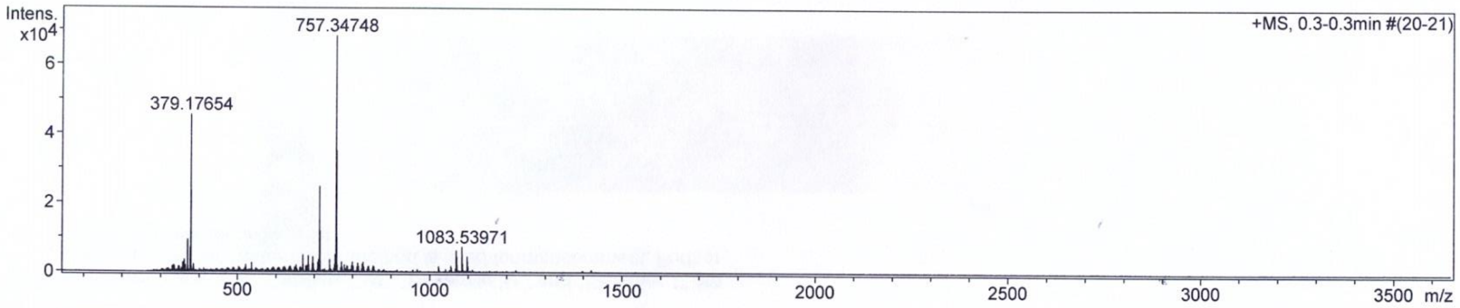

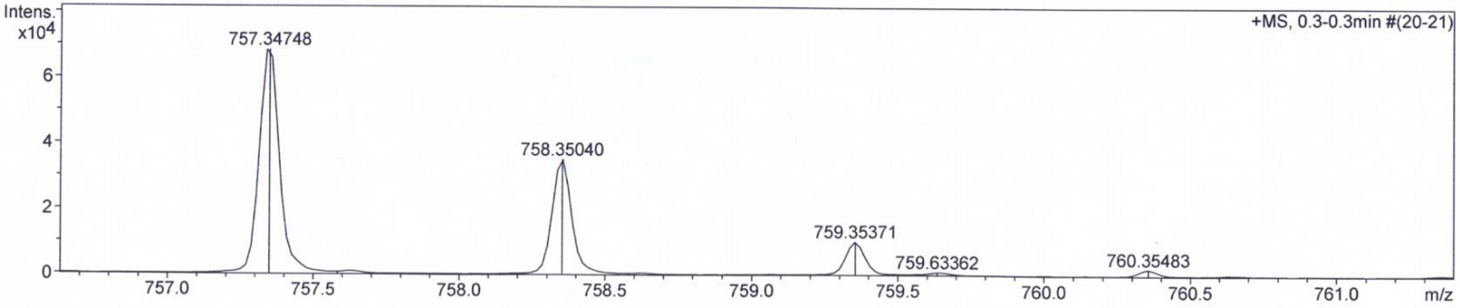

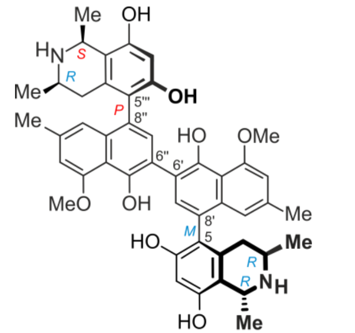

3

Sum Formula	Sigma	m/z	Err [ppm]	Mean Err [ppm]	rdb	N Rule	e⁻
C 46 H 49 N 2 O 8	0.00	757.34834	1.15	1.35	23.50	ok	even

Fig. S32 HRESIMS spectrum of compound **3**

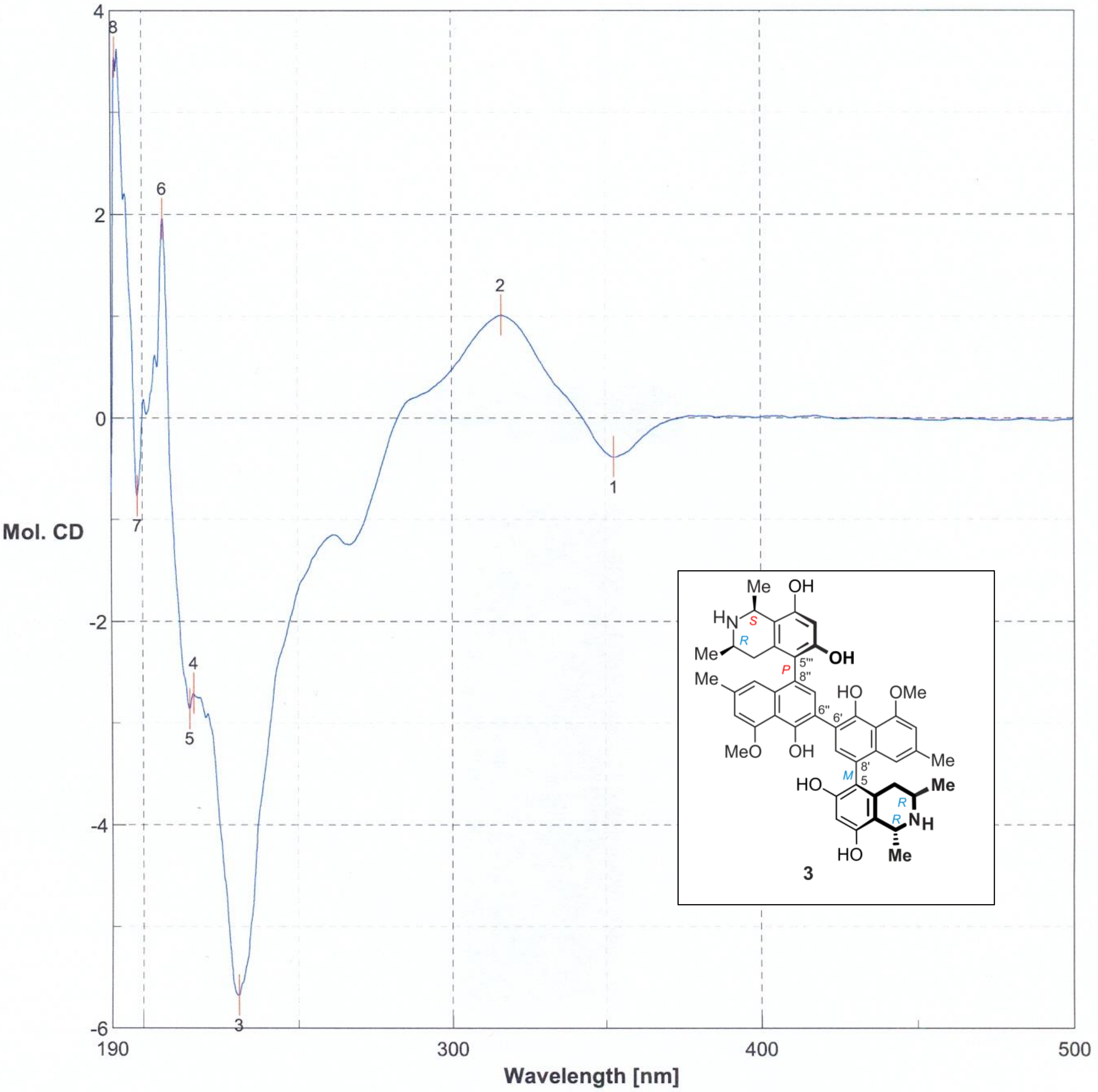

Date/Time	07.12.2015 17:57
Operator	kimbadi
File Name	BO3-H2O-6ok_0,5mm.jws
Sample Name	BO3-H2O-6_0.5mm_MeOH
Comment	offline

No.	nm	Mol. CD	No.	nm	Mol. CD	No.	nm	Mol. CD
1	352.6	-0.386955	2	316.1	1.00708	3	230.7	-5.67618
4	216.3	-2.70801	5	215	-2.85682	6	206.6	1.95653
7	198.3	-0.763366	8	191.3	3.54363			

Fig. S33 CD spectrum of compound **3** in methanol

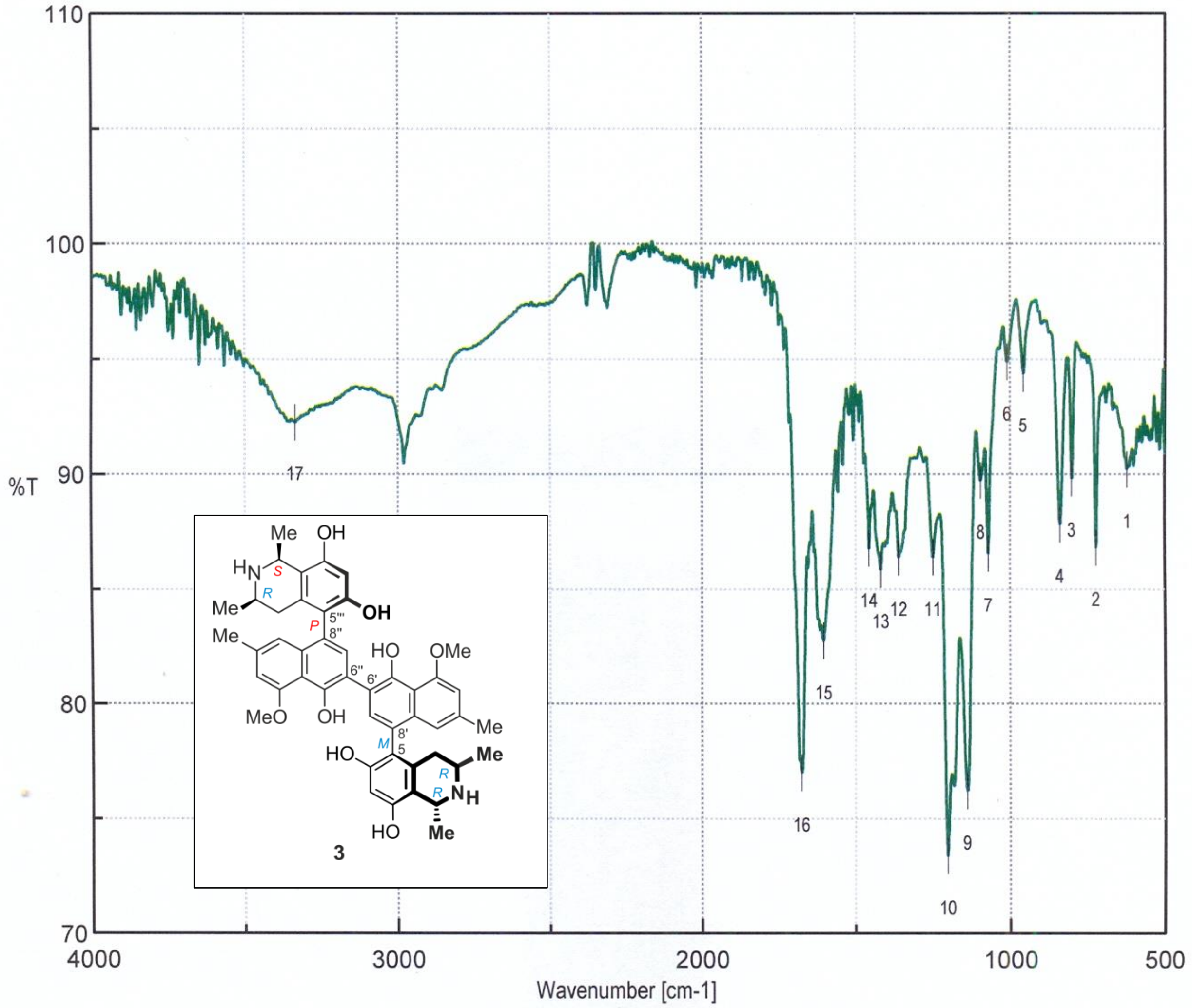

Results of Peak Find

No.	Position	Intensity	No.	Position	Intensity
1	622.895	90.1698	2	723.175	86.7789
3	801.278	89.7919	4	838.883	87.7775
5	957.484	94.3552	6	1009.55	94.8438
7	1071.26	86.5254	8	1096.33	89.6941
9	1138.76	76.1831	10	1202.4	73.3826
11	1250.61	86.3457	12	1360.53	86.3541
13	1418.39	85.8162	14	1456.96	86.7396
15	1603.52	82.7361	16	1674.87	76.9744
17	3335.28	92.2442			

[Comments]

Sample name	BO3-H2O-6
Comment	drop(MeOH)
User	Kimbadi
Division	AK Bringmann
Company	Uni Würzburg

[Measurement Information]

Model Name	FT/IR-4600typeA
Serial Number	D063461786
Accessory	ATR PRO ONE
Accessory S/N	A070661809
Incident angle	45 deg
Measurement Date	20.12.2017 14:53
Light Source	Standard
Detector	TGS
Accumulation	16
Resolution	4 cm−1
Zero Filling	On
Apodization	Cosine
Gain	Auto (4)
Aperture	Auto (7.1 mm)
Scanning Speed	Auto (2 mm/sec)
Filter	Auto (30000 Hz)

Fig. S34 IR spectrum of compound **3**

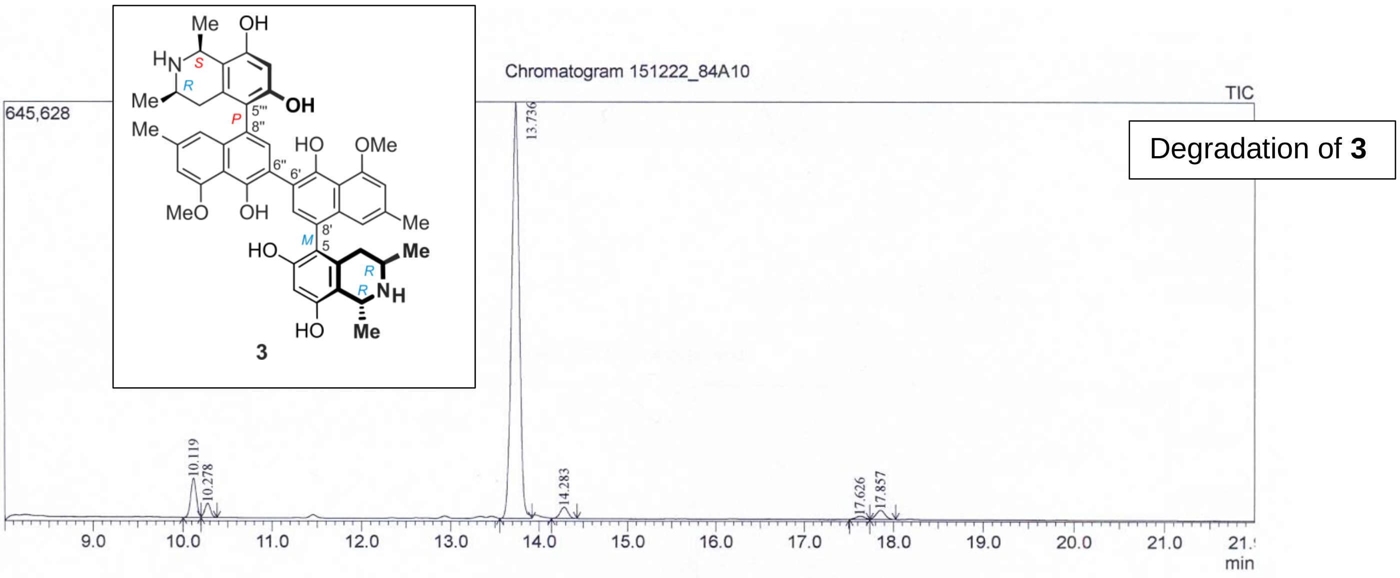

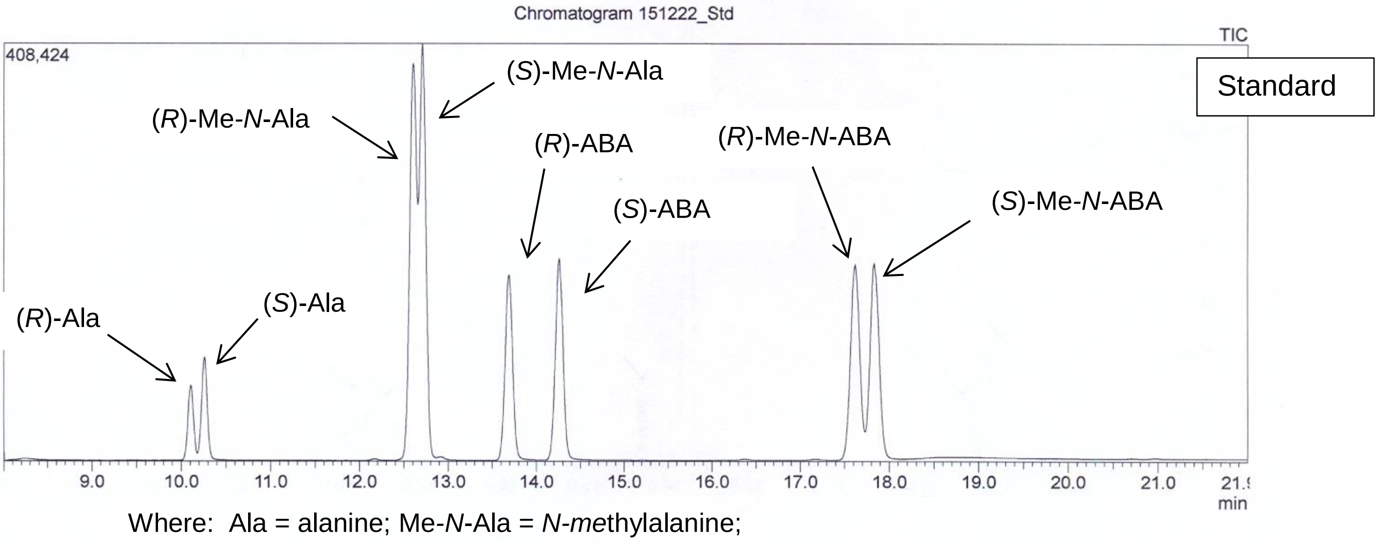

Where: Ala = alanine; Me-*N*-Ala = *N*-methylalanine;
ABA = 3-aminobyturic acid; Me-*N*-ABA = *N*-methyl 3-aminobyturic acid

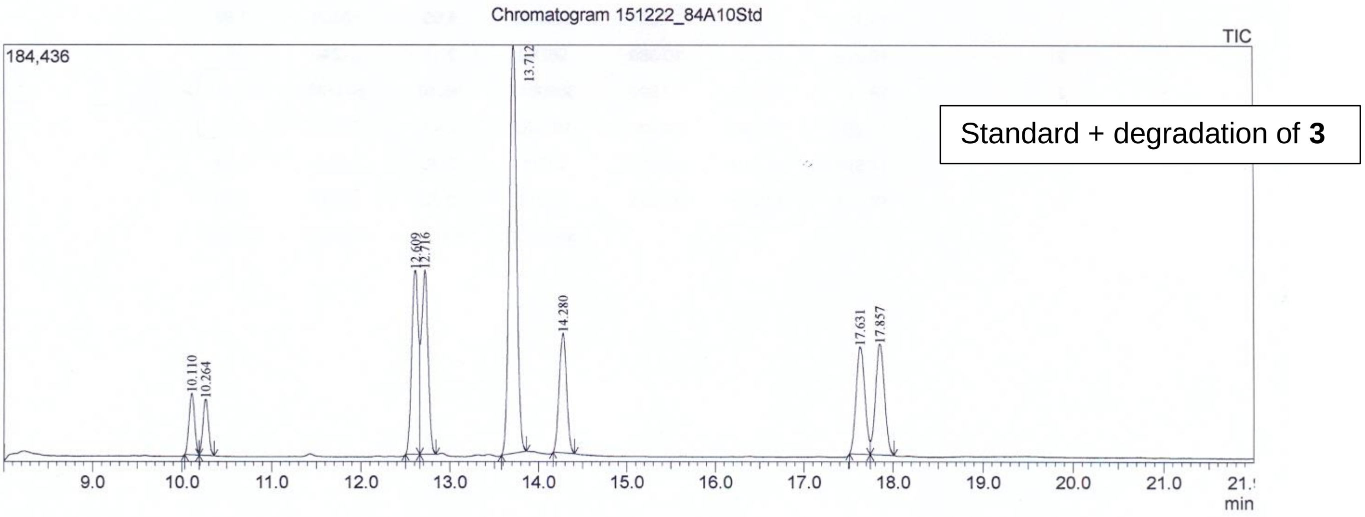

Fig. S35 Results of the oxidative degradation of compound **3**

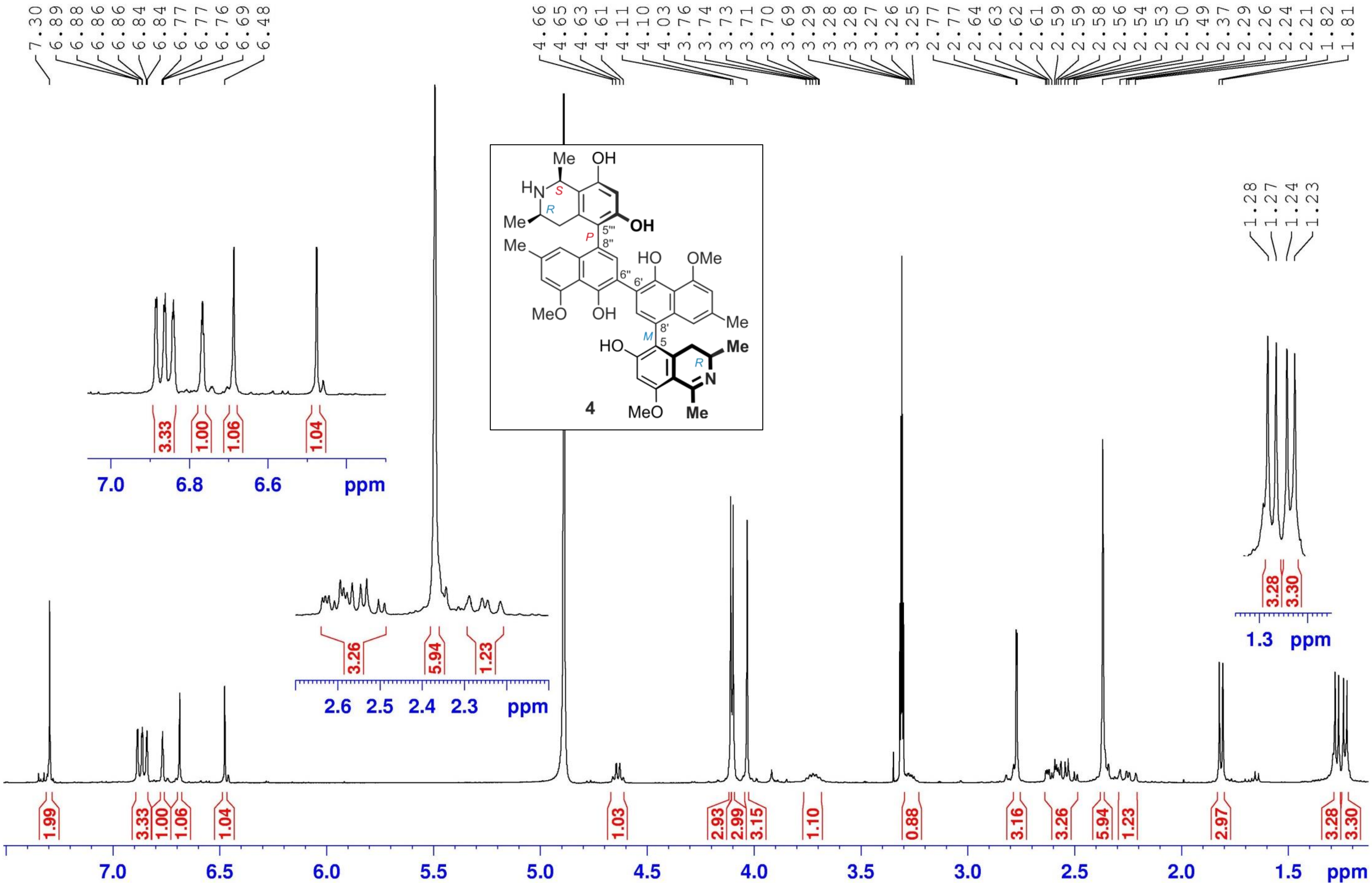

Fig. S36 ^{1}H NMR spectrum of compound **4** in methanol-d_4

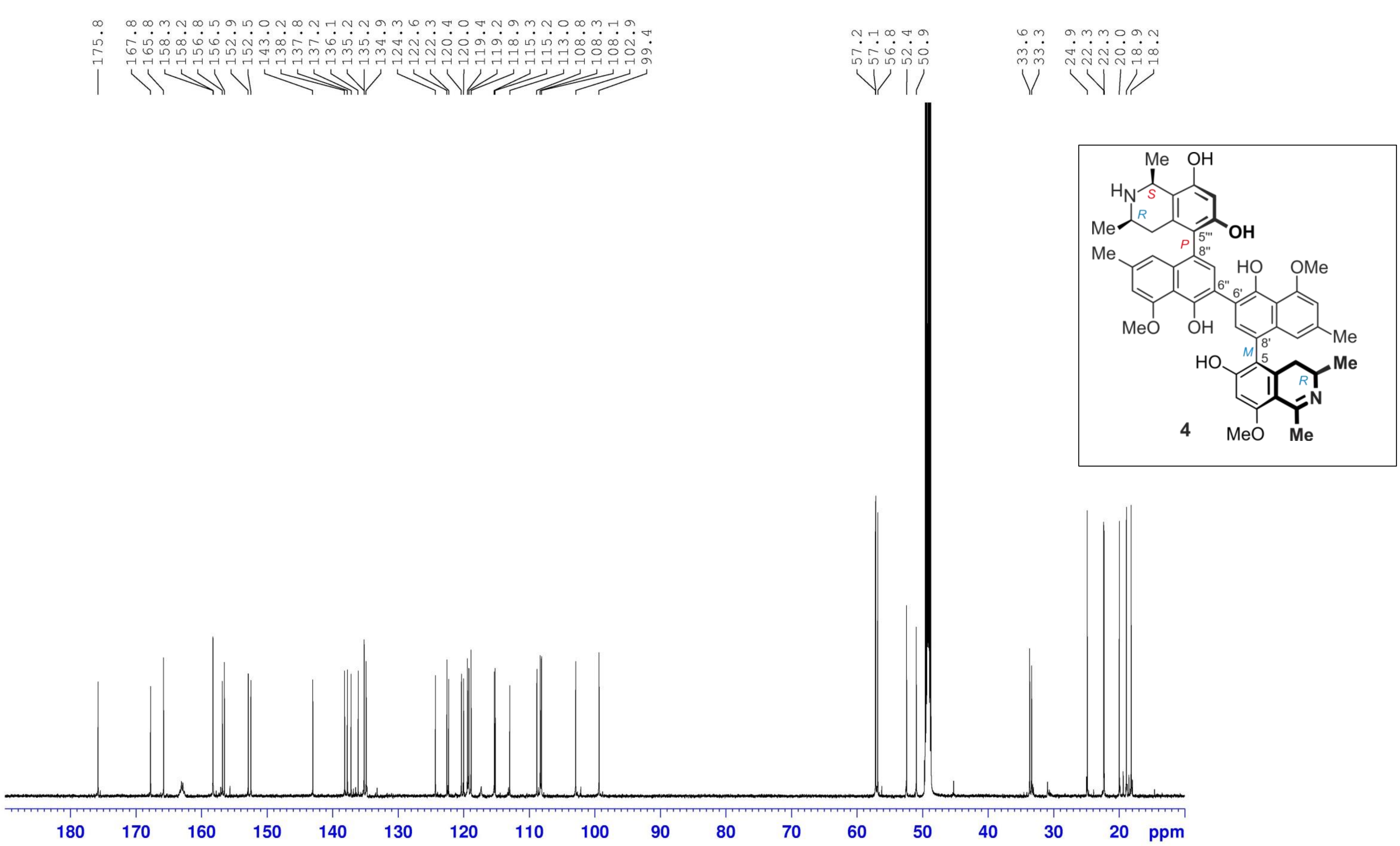

Fig. S37 ^{13}C NMR spectrum of compound **4** in methanol-d_4

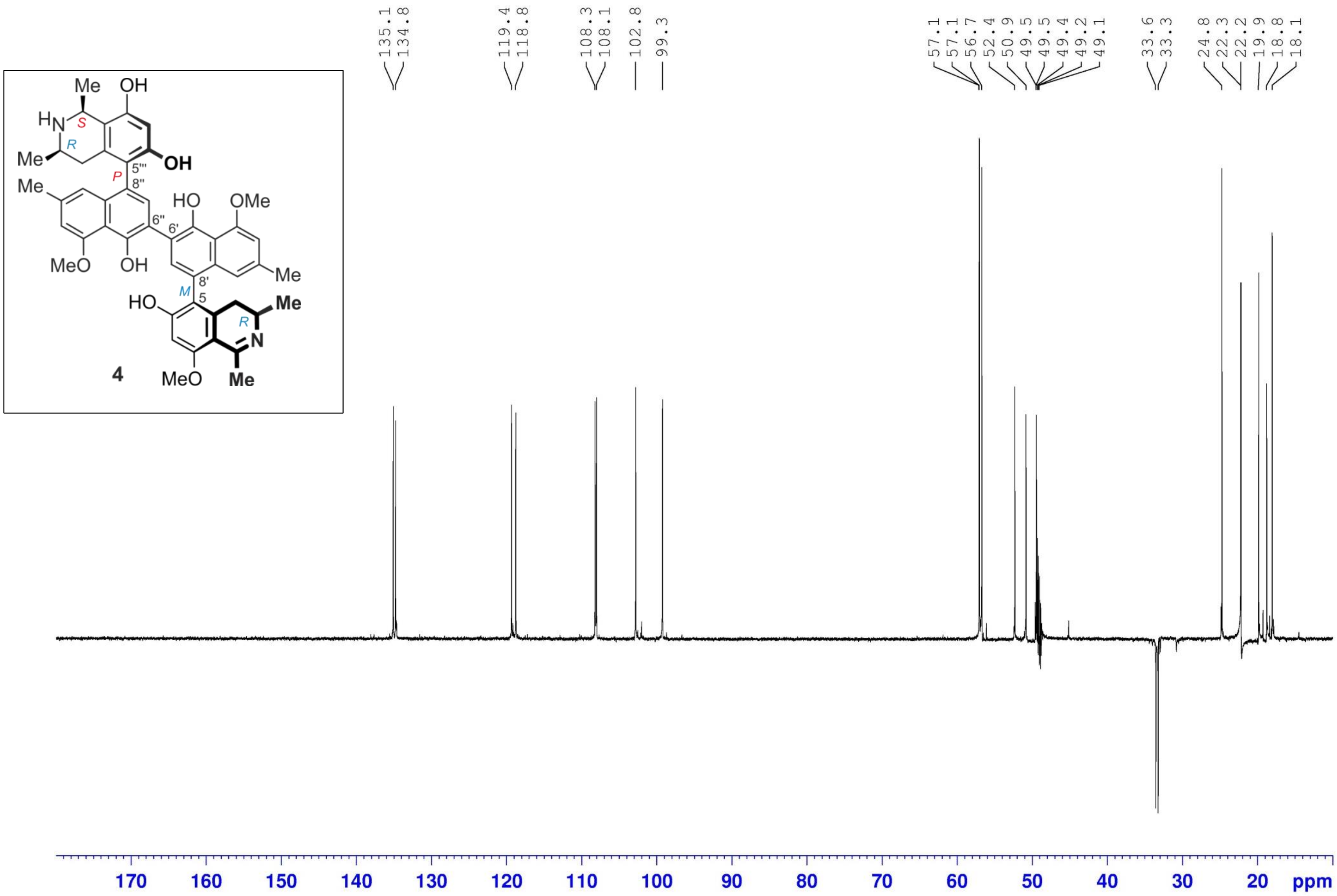

Fig. S38 DEPT-135 NMR spectrum of compound **4** in methanol-d_4

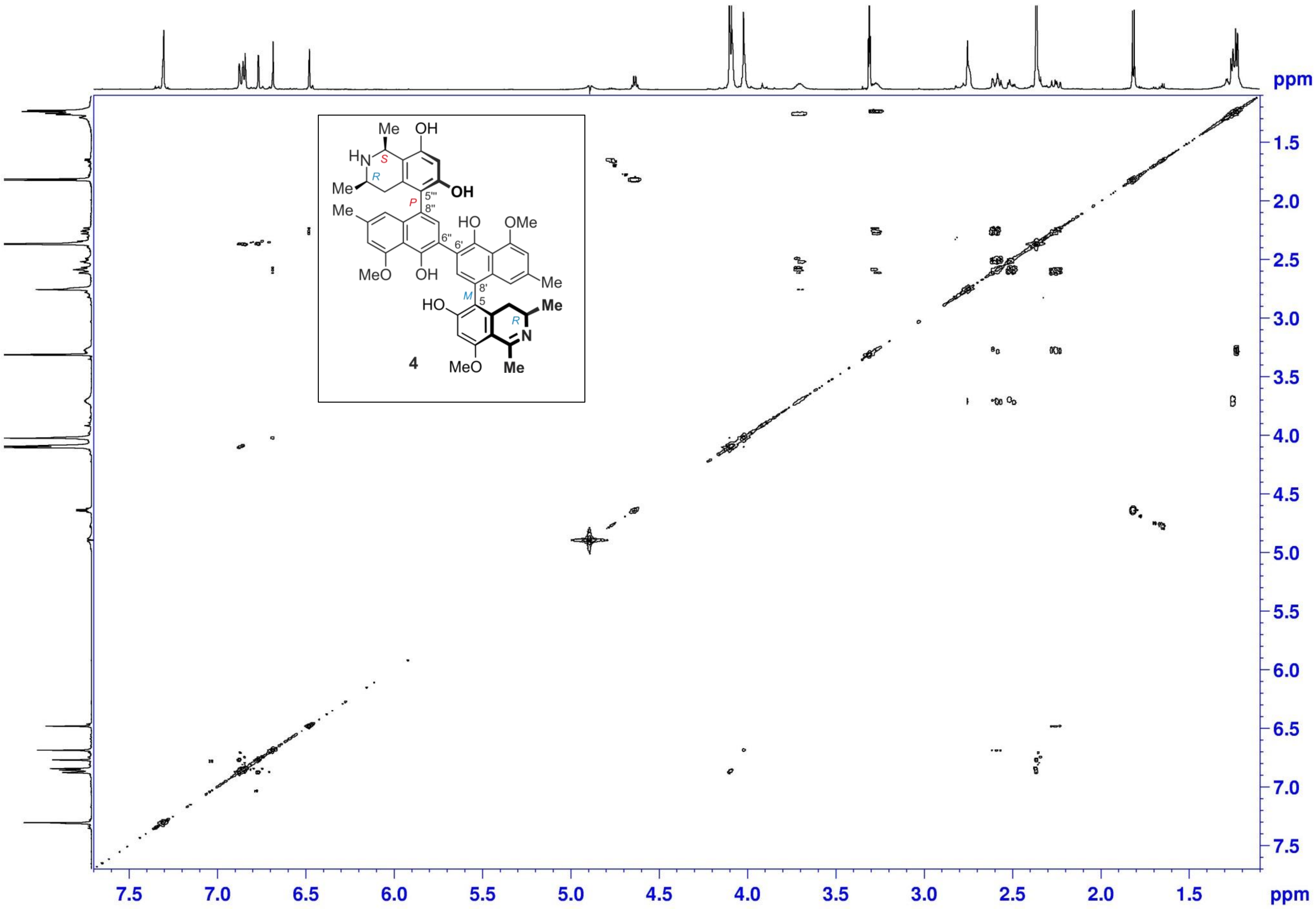

Fig. S39 COSY NMR spectrum of compound **4** in methanol-d_4

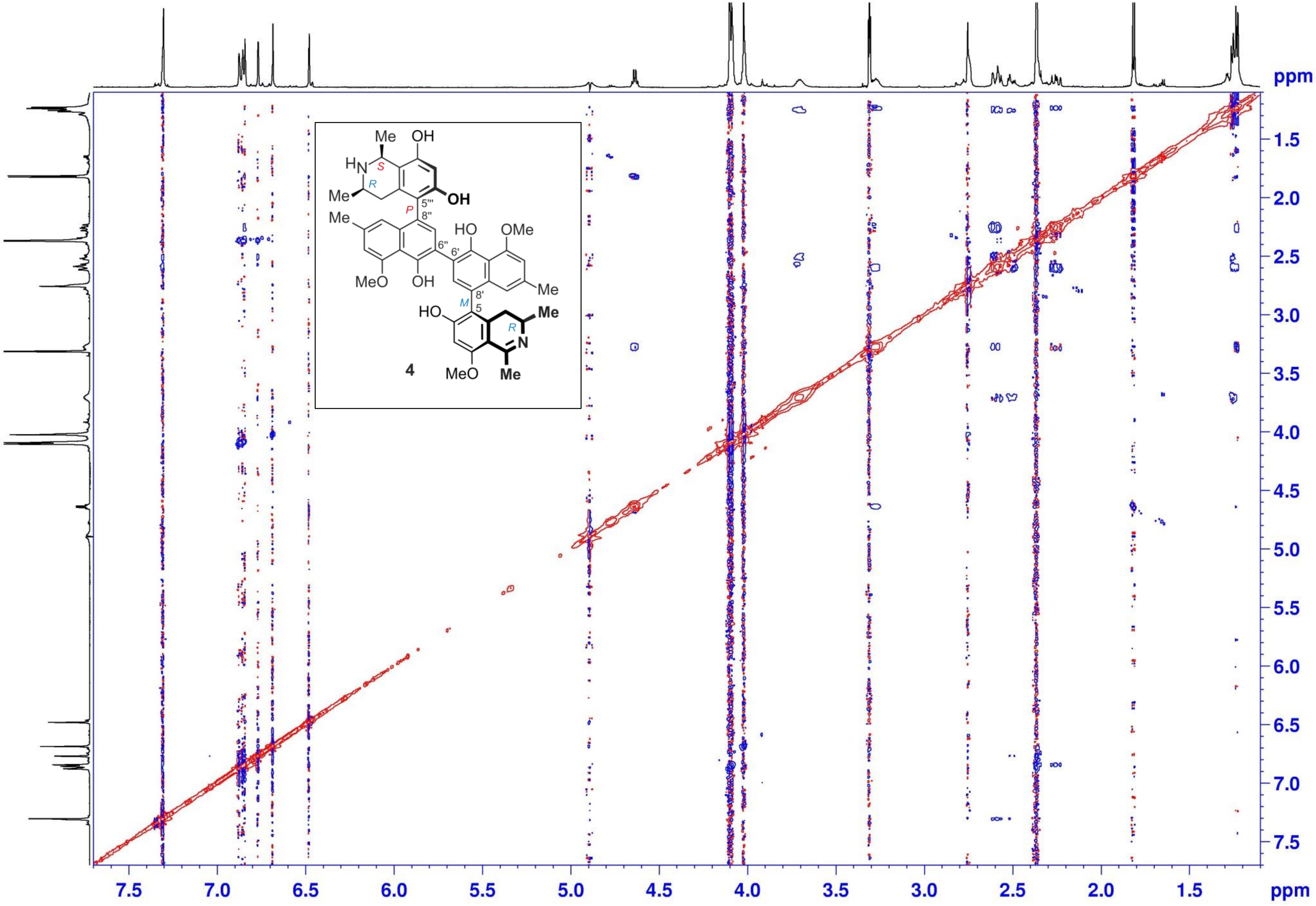

Fig. S40 ROESY NMR spectrum of compound **4** in methanol-d_4

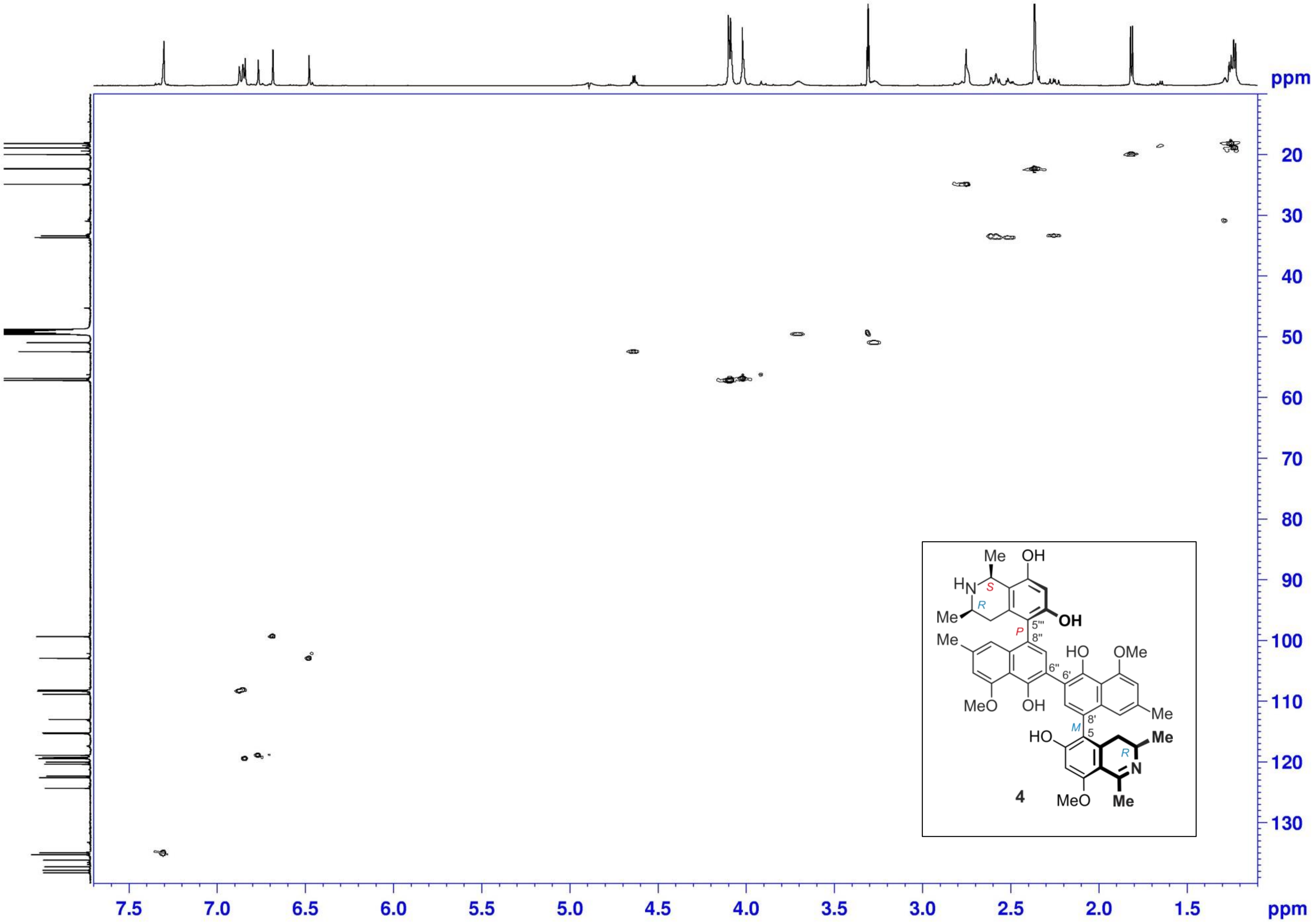

Fig. S41 HSQC NMR spectrum of compound **4** in methanol-d_4

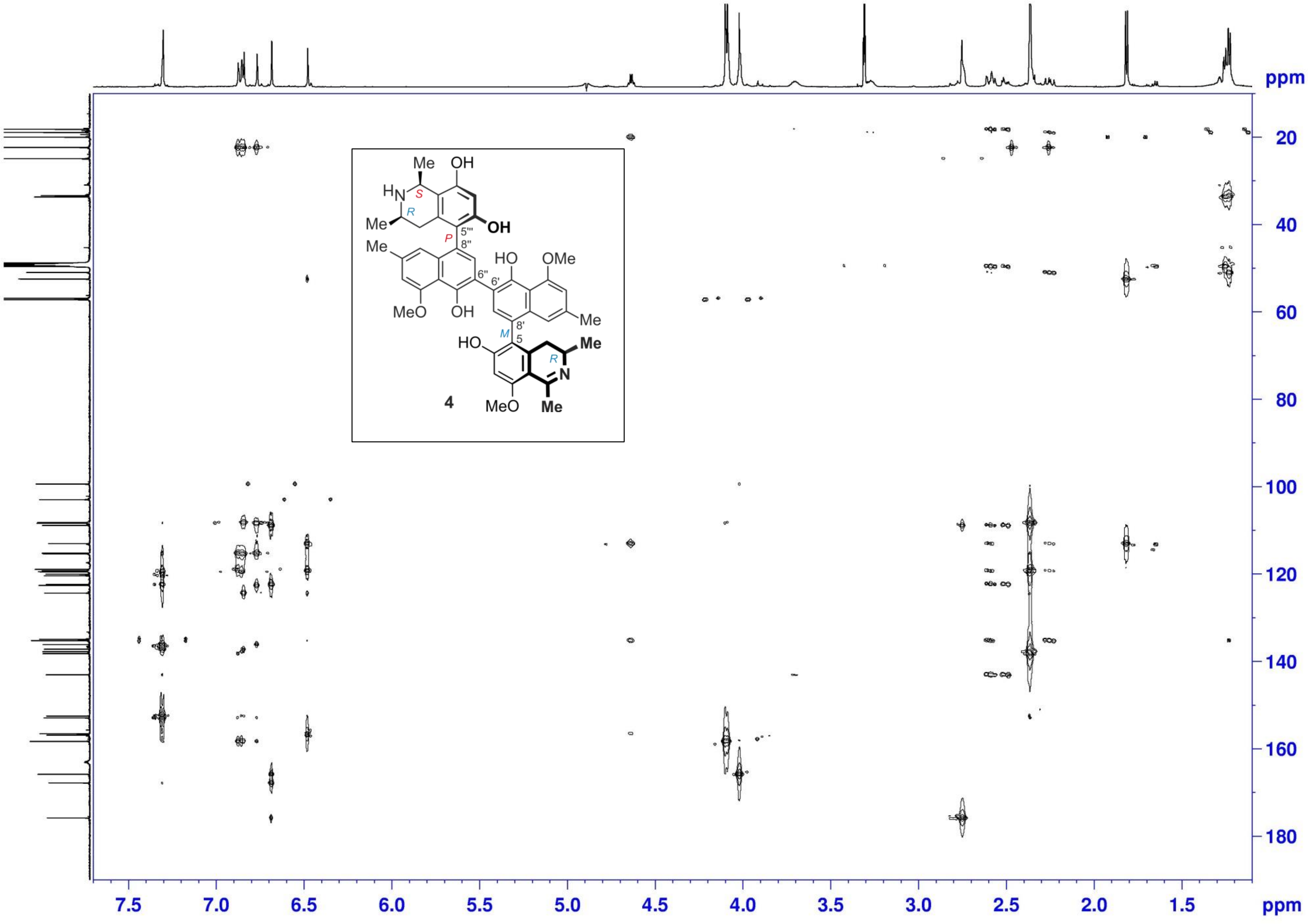

Fig. S42 HMBC NMR spectrum of compound **4** in methanol-d_4

Mass Spectrum SmartFormula Report

Analysis Info		Acquisition Date	10/18/2017 12:39:58 PM	
Analysis Name	D:\Data\Spektren2017\2017_3186_BRI_BO3-H2O-8p_27_01_2707.d			
Method	automation_esi_tune_pos_mid_ja_meoh.m	Operator	J.Adelmann	
Sample Name	2017_3186_BRI_BO3-H2O-8p	Instrument	micrOTOF-Q III	8228888.20516
Comment	Kimbadi Lombe Blaise<br>BO3-H2O-8p<br>8pmol/ul in MeOH			

Acquisition Parameter					
Source Type	ESI	Ion Polarity	Positive	Set Nebulizer	0.7 Bar
Focus	Not active	Set Funnel 1 RF	300.0 Vpp	Set Dry Heater	200 °C
Scan Begin	200 m/z	Set Funnel 2 RF	400.0 Vpp	Set Dry Gas	5.0 l/min
Scan End	3500 m/z	Set Hexapole RF	500.0 Vpp	Set Divert Valve	Source

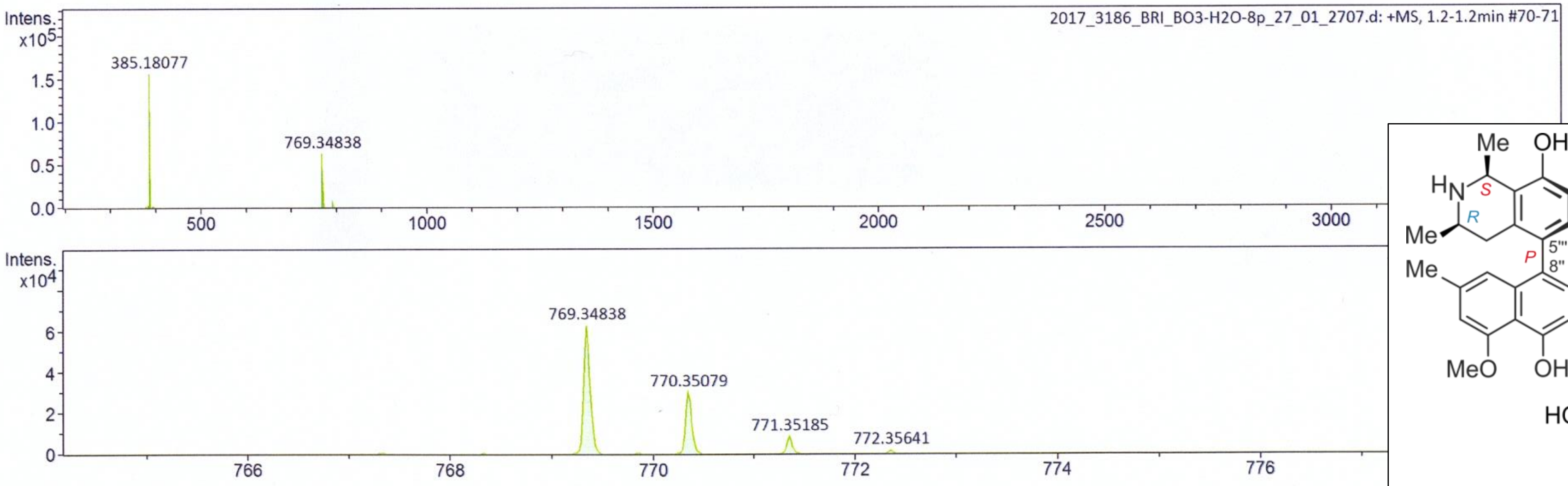

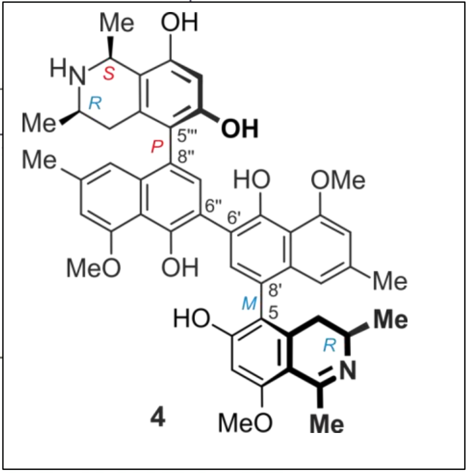

Meas. m/z	#	Ion Formula	m/z	err [ppm]	mSigma	# mSigma	Score	rdb	e⁻ Conf	N-Rule
769.34838	1	C47H49N2O8	769.34834	-0.04	25.0	1	100.00	24.5	even	ok

Bruker Compass DataAnalysis 4.2 printed: 10/18/2017 1:54:00 PM by: J.Adelmann Page 1 of 1

Fig. S43 HRESIMS spectrum of compound **4**

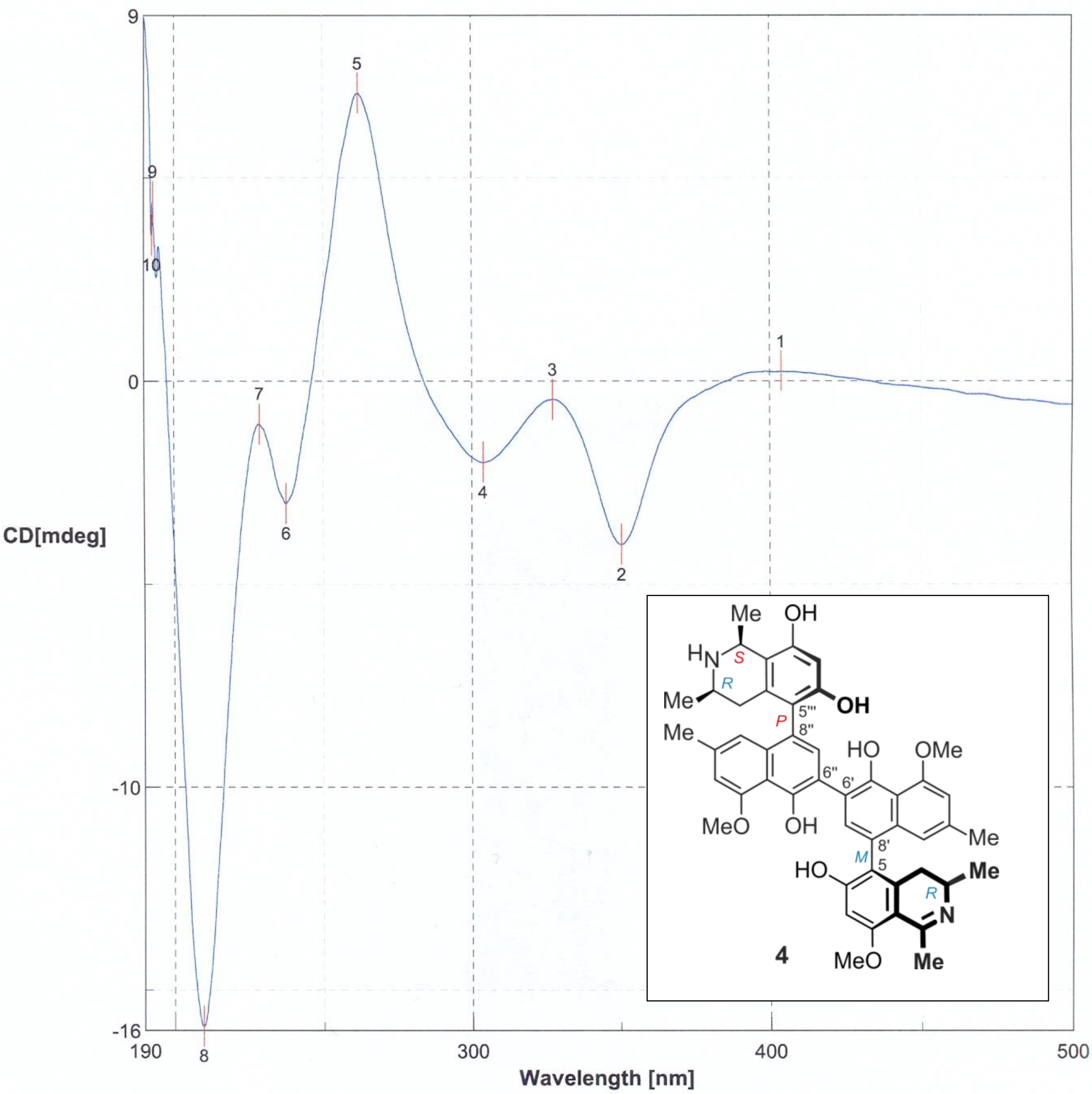

Date/Time	20.12.2017 18:28
Operator	Kimbadi
File Name	BO3-H2O-8_0#005.jws
Sample Name	BO3-H2O-8_0
Comment	offline

No.	nm	CD[mdeg]	No.	nm	CD[mdeg]	No.	nm	CD[mdeg]
1	403.8	0.232944	2	350.3	-4.02852	3	327.3	-0.458048
4	303.8	-2.00276	5	261.8	7.08695	6	237.4	-3.01912
7	228.5	-1.05888	8	209.5	-15.8847	9	193	4.42249
10	192.6	3.59684						

Fig. S44 CD spectrum of compound **4** in methanol

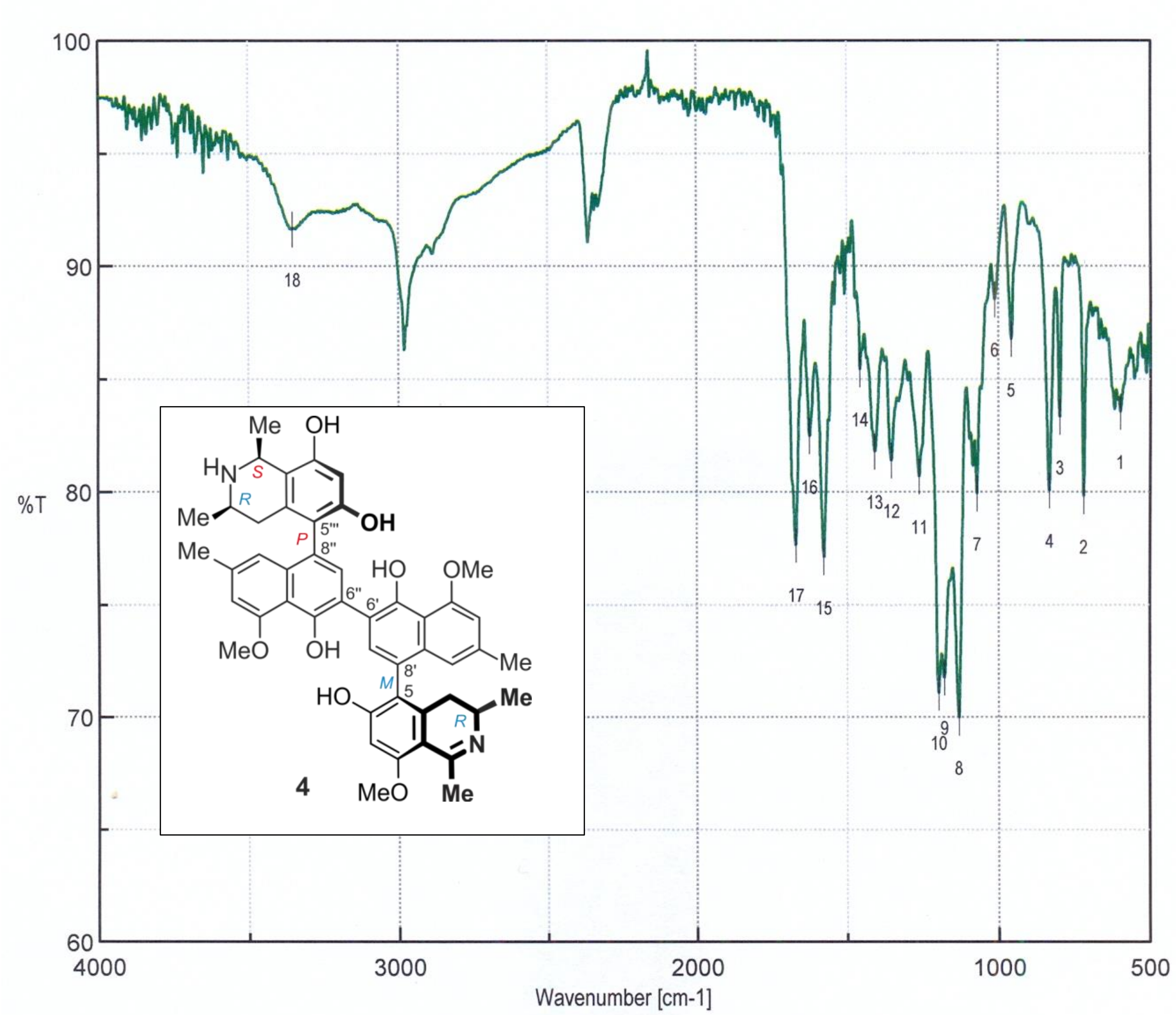

Results of Peak Find

No.	Position	Intensity	No.	Position	Intensity
1	598.789	83.5223	2	720.282	79.775
3	798.385	83.2913	4	832.133	80.0182
5	957.484	86.7518	6	1011.48	88.4989
7	1071.26	79.9095	8	1132.01	69.9563
9	1180.22	71.7542	10	1198.54	71.0595
11	1262.18	80.6436	12	1353.78	81.3499
13	1407.78	81.7623	14	1455.99	85.4004
15	1577.49	77.0809	16	1623.77	82.4294
17	1670.05	77.6268	18	3353.6	91.6289

[Comments]

Sample name	BO3-H2O-8a
Comment	drop(MeOH)
User	Kimbadi
Division	AK Bringmann
Company	Uni Würzburg

[Measurement Information]

Model Name	FT/IR-4600typeA
Serial Number	D063461786
Accessory	ATR PRO ONE
Accessory S/N	A070661809
Incident angle	45 deg
Measurement Date	20.12.2017 14:32
Light Source	Standard
Detector	TGS
Accumulation	16
Resolution	4 cm-1
Zero Filling	On
Apodization	Cosine
Gain	Auto (4)
Aperture	Auto (7.1 mm)
Scanning Speed	Auto (2 mm/sec)
Filter	Auto (30000 Hz)

Fig. S45 IR spectrum of compound **4**

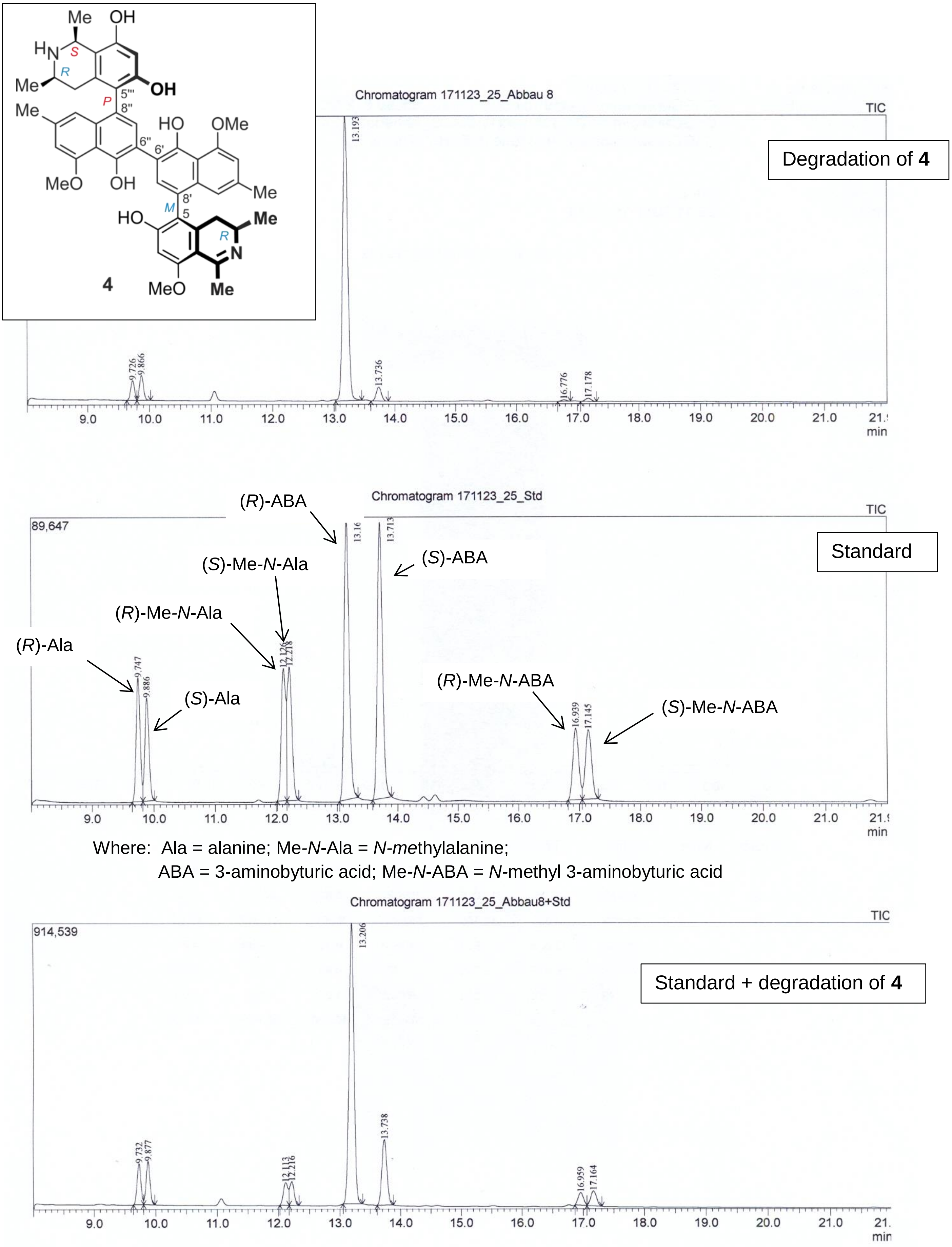

Fig. S46 Results of the oxidative degradation of compound **4**

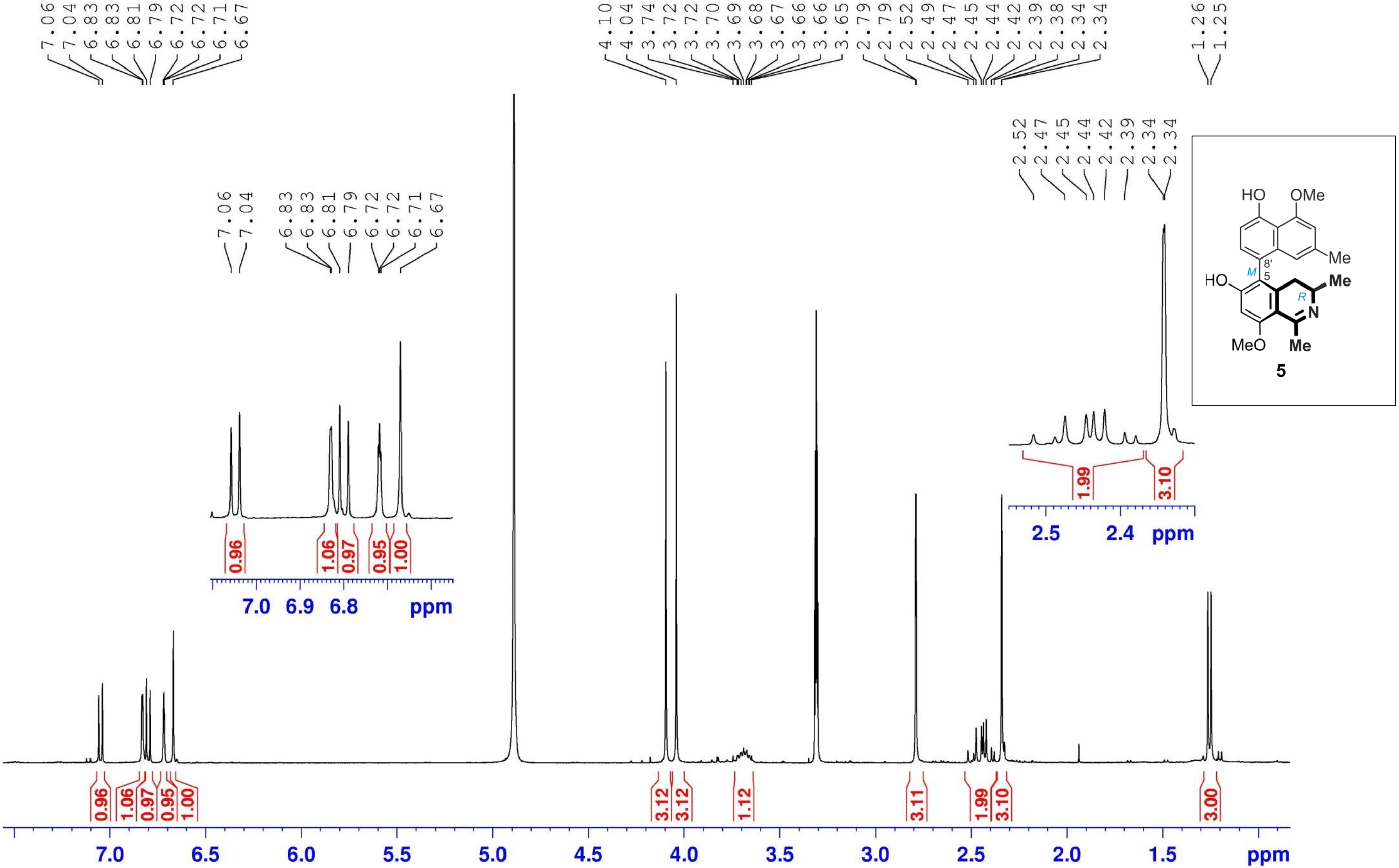

Fig. S47 ^{1}H NMR spectrum of compound **5** in methanol-d_4

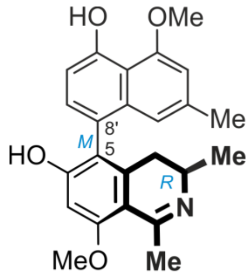

5

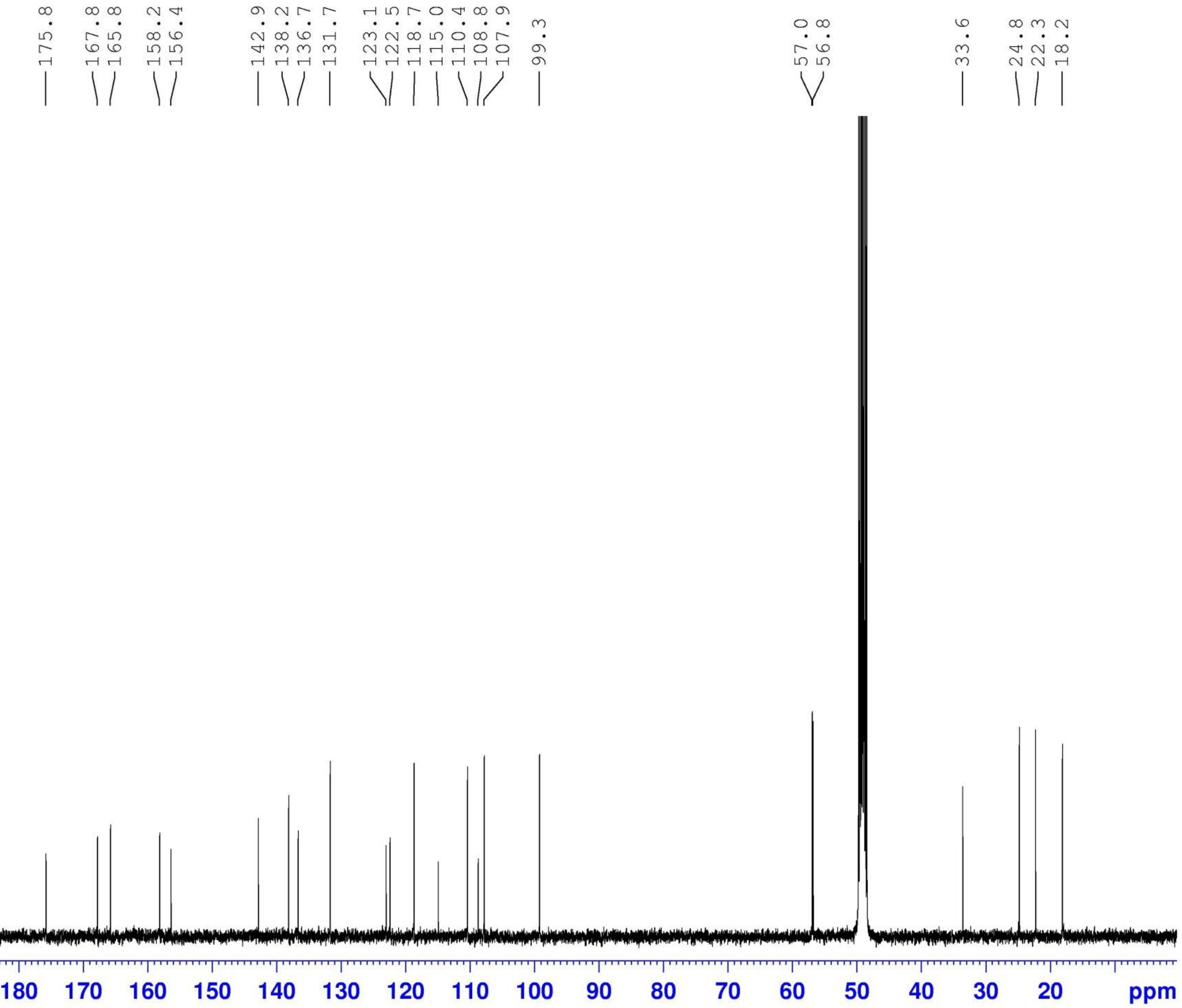

Fig. S48 ^{13}C NMR spectrum of compound **5** in methanol-d_4

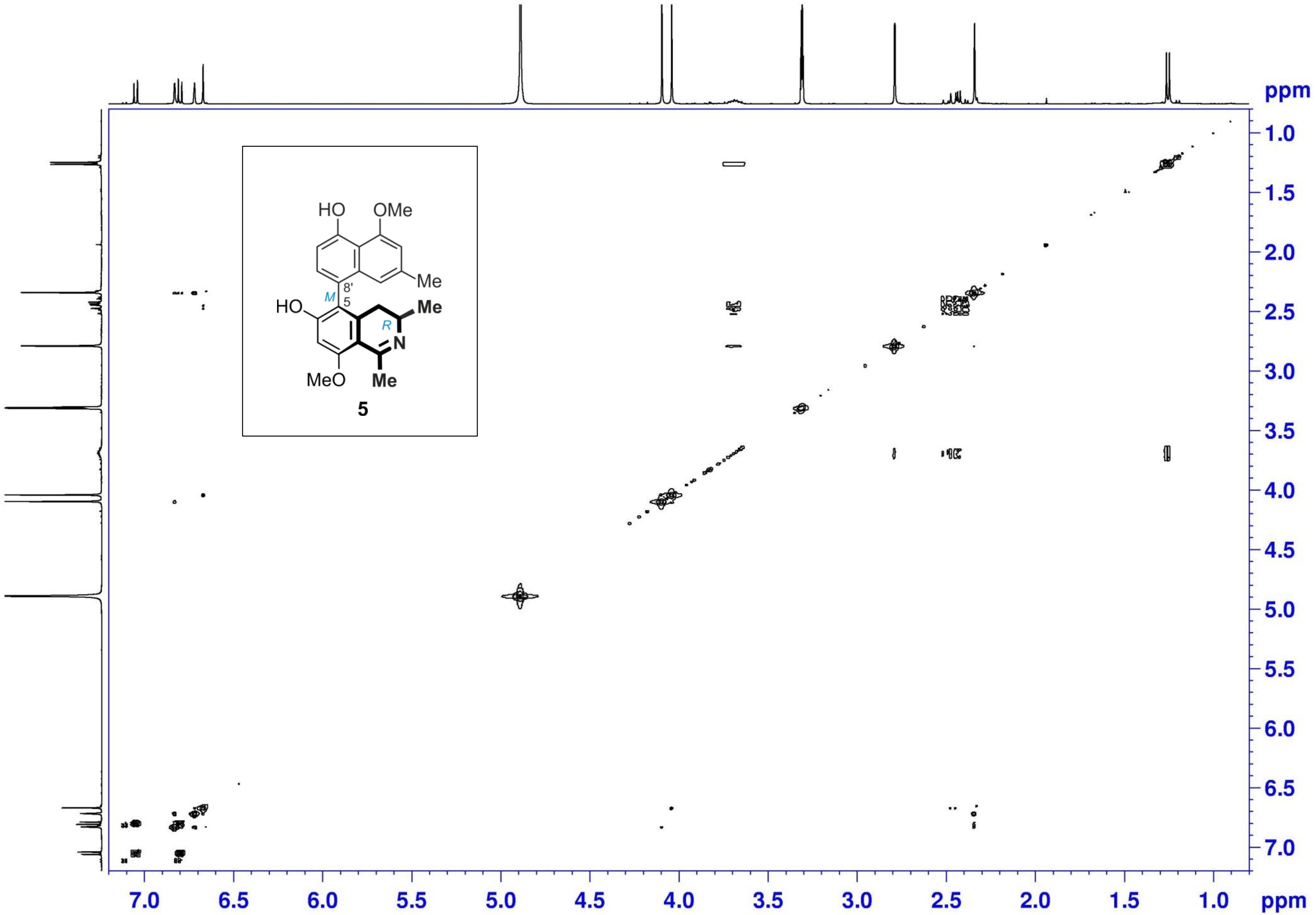

Fig. S49 COSY NMR spectrum of compound **5** in methanol-d_4

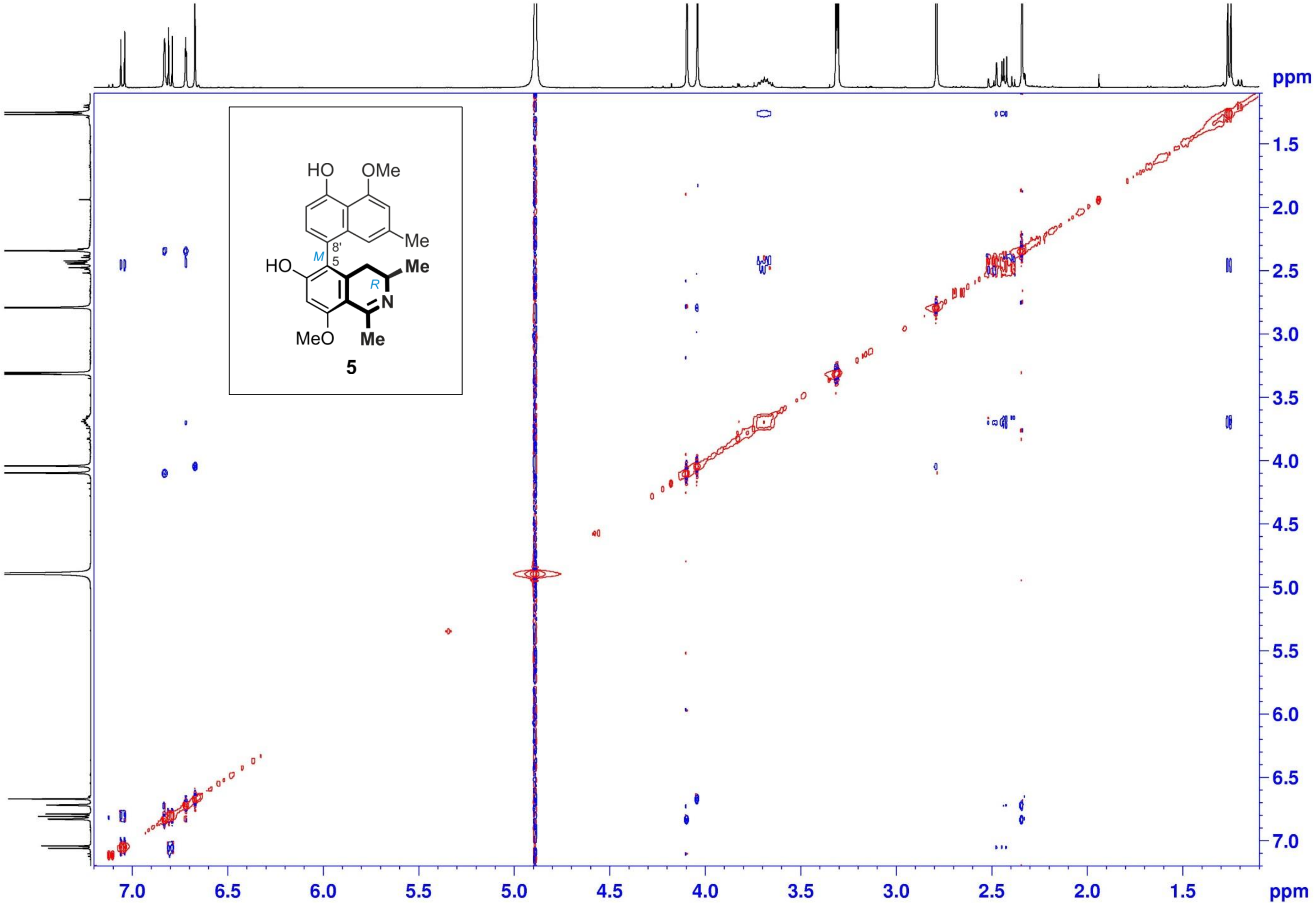

Fig. S50 NOESY NMR spectrum of compound **5** in methanol-d_4

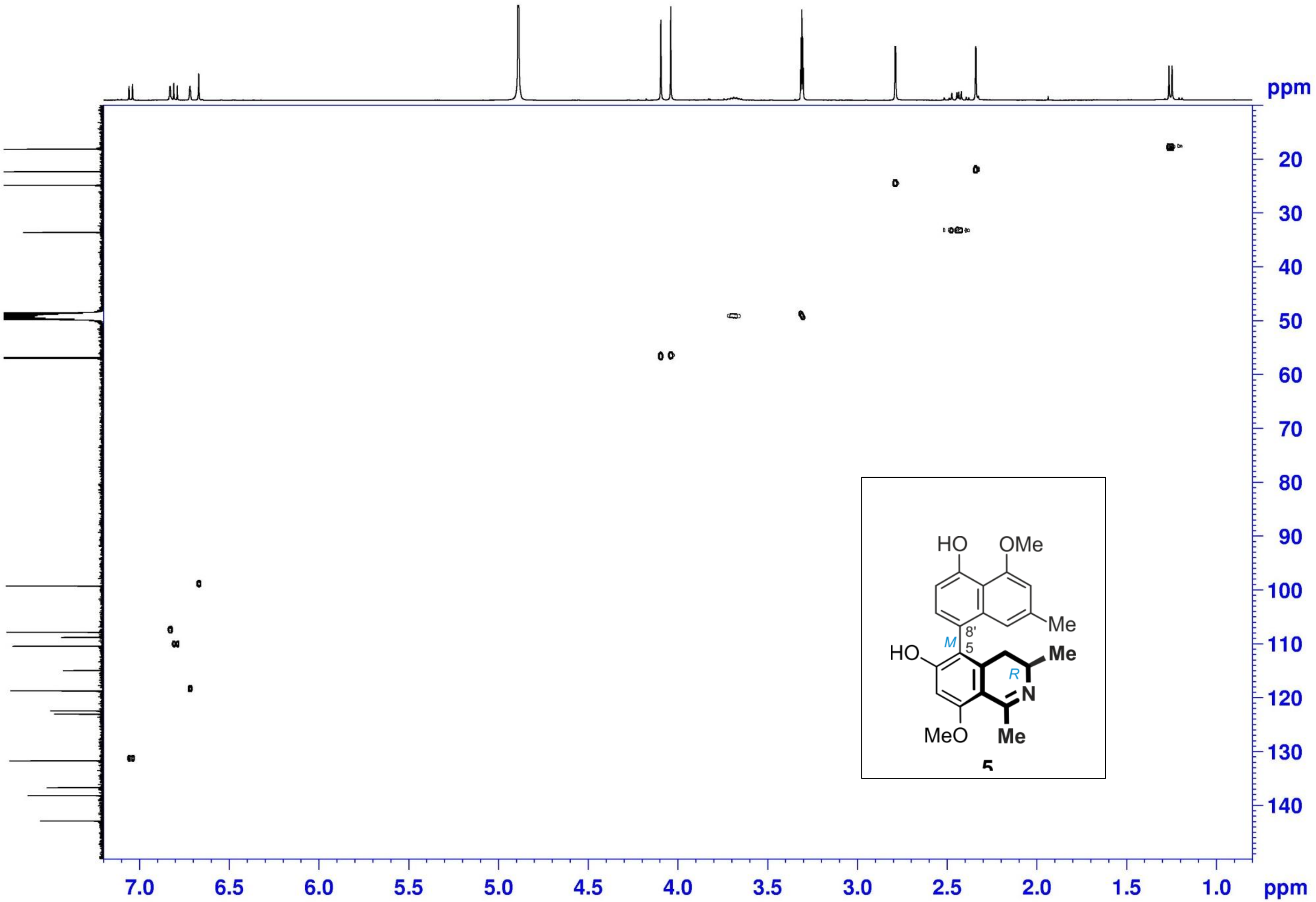

Fig. S51 HSQC NMR spectrum of compound **5** in methanol-d_4

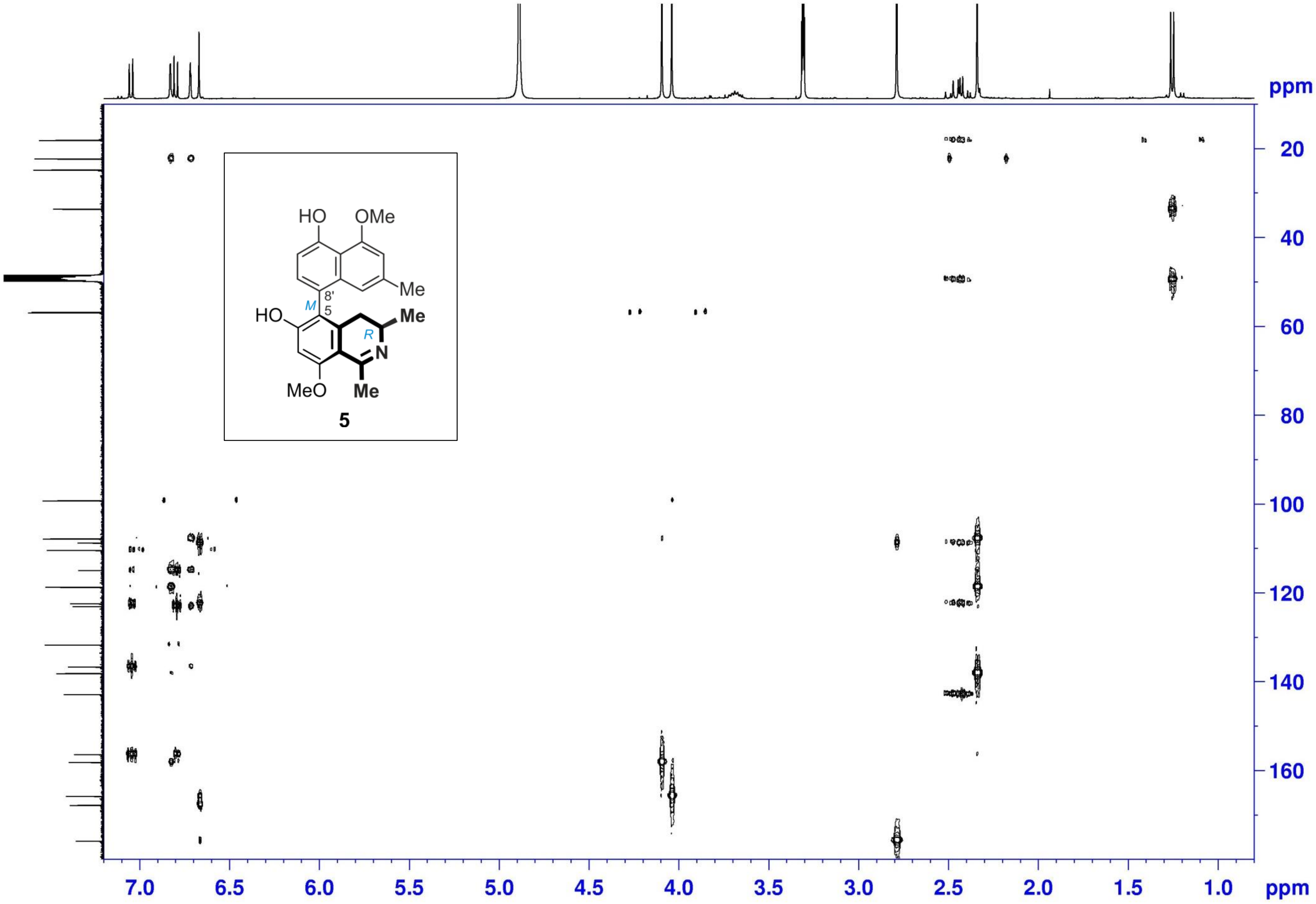

Fig. S52 HMBC NMR spectrum of compound **5** in methanol-d_4

Bruker Daltonics DataAnalysis 3.3

Mass Spectrum Molecular Formula Report

Analysis Info

Analysis Name	D:\Data\Spektren2015\2015_2669_BRI_34_01_1128.d	Acquisition Date	22.12.2015 11:25:32
Method	automation_esi_tune_pos_low_JA_MeOH.m	Operator	Administrator
Comment	Blaise Kimbadi Lombe<br>B03-DCM-1<br>4 pMol/µL in MeOH	Instrument	micrOTOF 88

Acquisition Parameter

Source Type	ESI	Ion Polarity	Positive	Set Corrector Fill	48 V
Scan Range	n/a	Capillary Exit	100.0 V	Set Pulsar Pull	804 V
Scan Begin	50 m/z	Hexapole RF	80.0 V	Set Pulsar Push	807 V
Scan End	3500 m/z	Skimmer 1	50.0 V	Set Reflector	1700 V
		Hexapole 1	23.0 V	Set Flight Tube	8600 V
				Set Detector TOF	2240 V

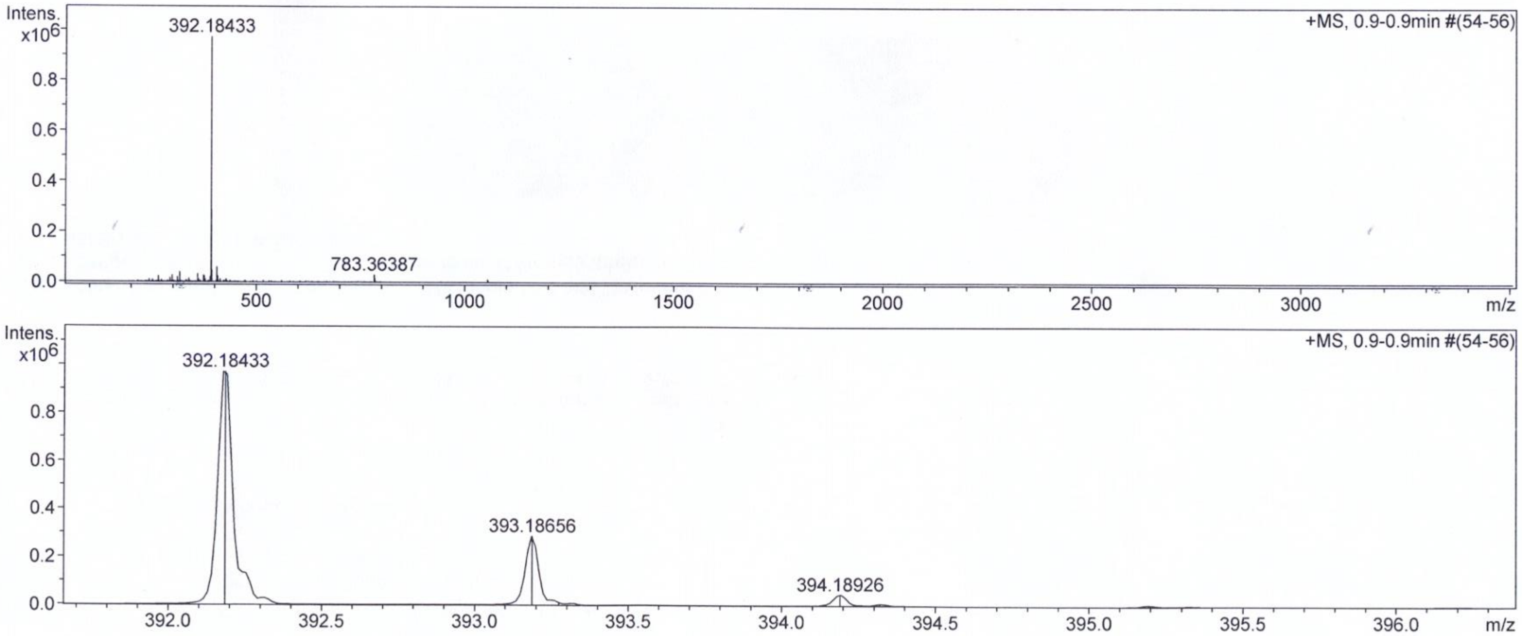

Sum Formula	Sigma	m/z	Err [ppm]	Mean Err [ppm]	rdb	N Rule	e⁻
C 24 H 26 N 1 O 4	0.01	392.18563	3.33	4.01	12.50	ok	even

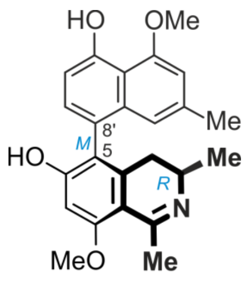

5

Fig. S53 HRESIMS spectrum of compound **5**

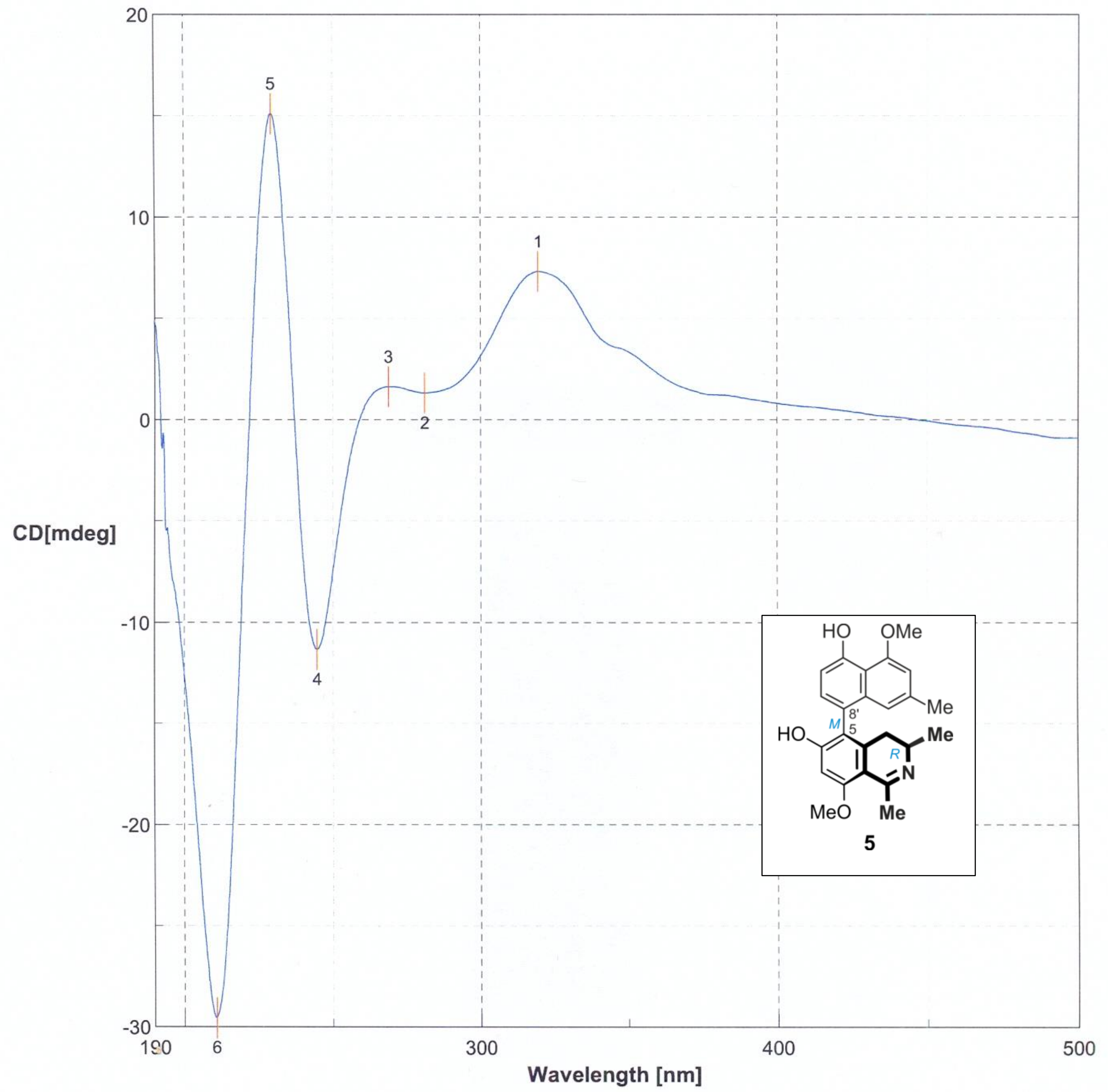

Date/Time	07.12.2015 14:49
Operator	kimbadi
File Name	BO3-DCM-1_0#005.jws
Sample Name	BO3-DCM-1_0.5mm_MeOH
Comment	offline

No.	nm	CD[mdeg]	No.	nm	CD[mdeg]	No.	nm	CD[mdeg]
1	319.6	7.31576	2	281.1	1.31883	3	268.8	1.63188
4	244.6	-11.3299	5	229.3	15.1054	6	210.8	-29.5241

Fig. S54 CD spectrum of compound **5** in methanol

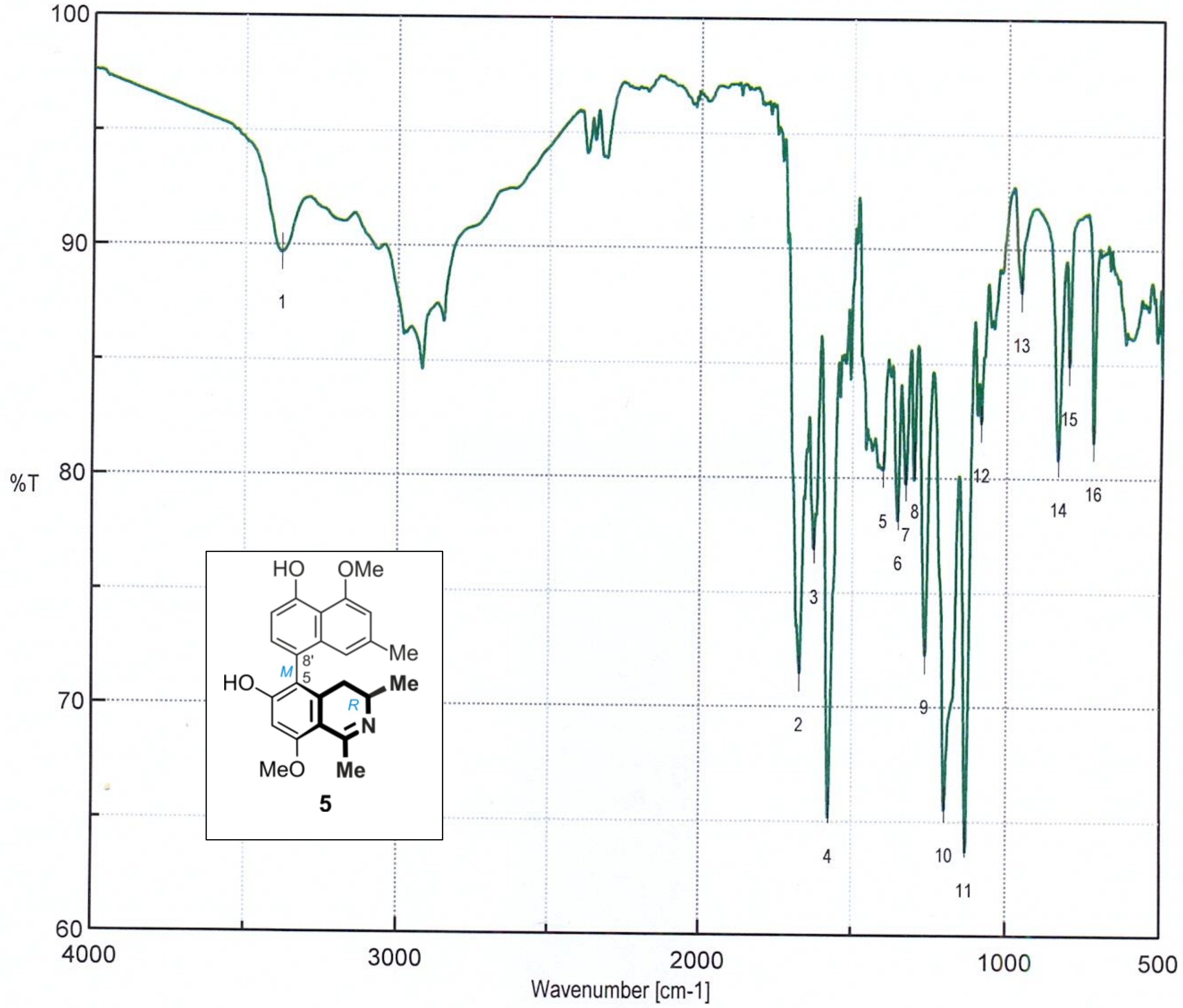

Results of Peak Find

No.	Position	Intensity	No.	Position	Intensity
1	3381.57	89.6804	2	1671.02	71.4524
3	1624.73	77.0215	4	1574.59	65.7072
5	1400.07	80.3554	6	1351.86	78.505
7	1325.82	79.7662	8	1296.89	80.7913
9	1264.11	72.2617	10	1197.58	65.779
11	1132.01	64.2582	12	1083.8	82.3768
13	955.555	88.0809	14	832.133	80.8874
15	799.35	84.884	16	720.282	81.586

[Comments]

Sample name	BO3-DCM-1
Comment	drop(MeOH)
User	Kimbadi
Division	AK Bringmann
Company	Uni Würzburg

[Measurement Information]

Model Name	FT/IR-4600typeA
Serial Number	D063461786
Accessory	ATR PRO ONE
Accessory S/N	A070661809
Incident angle	45 deg
Measurement Date	20.12.2017 15:20
Light Source	Standard
Detector	TGS
Accumulation	16
Resolution	4 cm-1
Zero Filling	On
Apodization	Cosine
Gain	Auto (4)
Aperture	Auto (7.1 mm)
Scanning Speed	Auto (2 mm/sec)
Filter	Auto (30000 Hz)

Fig. S55 IR spectrum of compound **5**

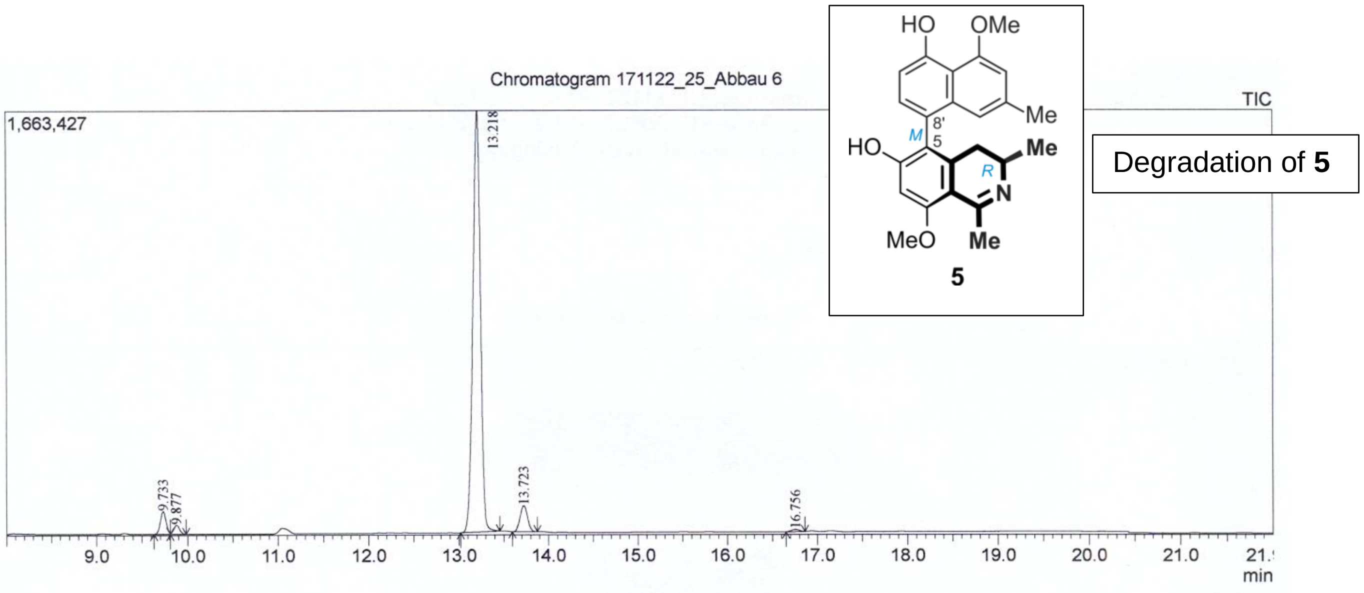

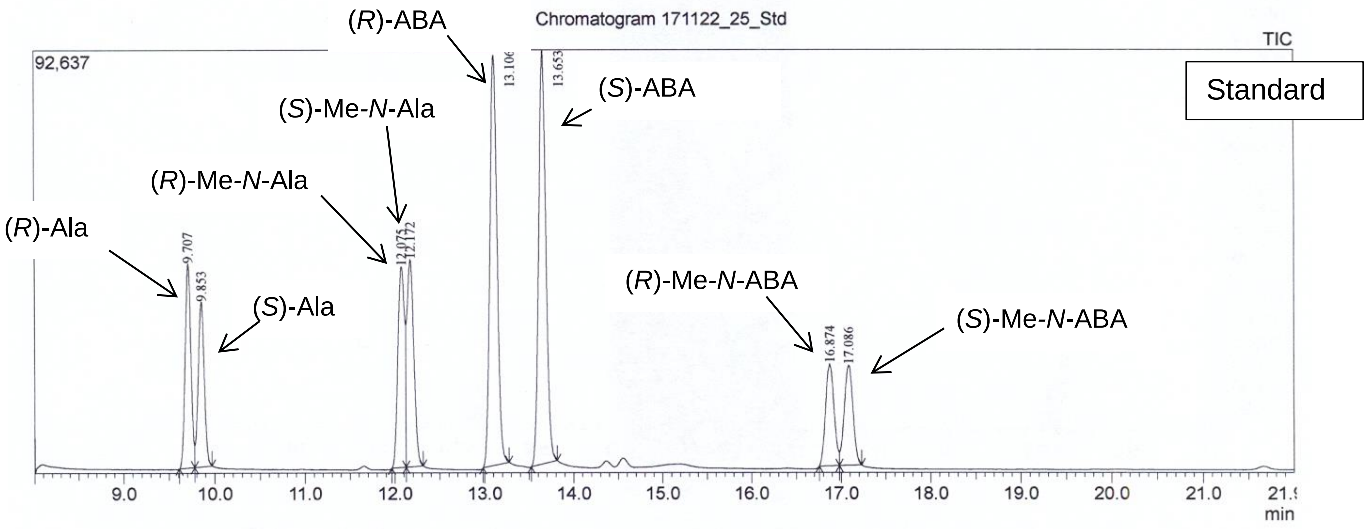

Where: Ala = alanine; Me-*N*-Ala = *N*-*me*thylalanine;
ABA = 3-aminobyturic acid; Me-*N*-ABA = *N*-methyl 3-aminobyturic acid

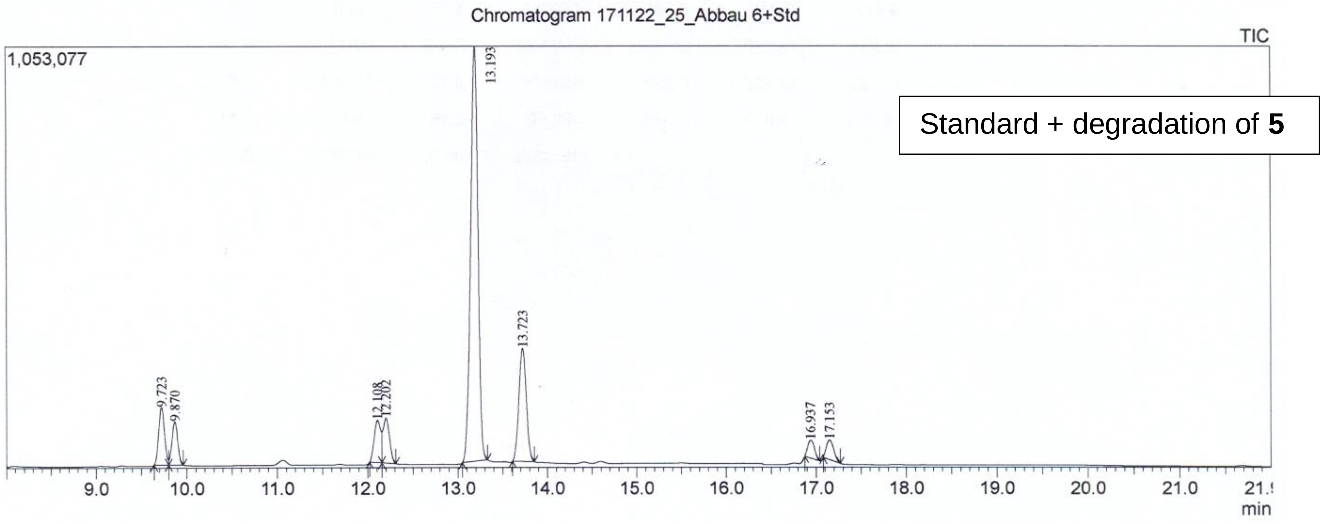

Fig. S56 Results of the oxidative degradation of compound **5**

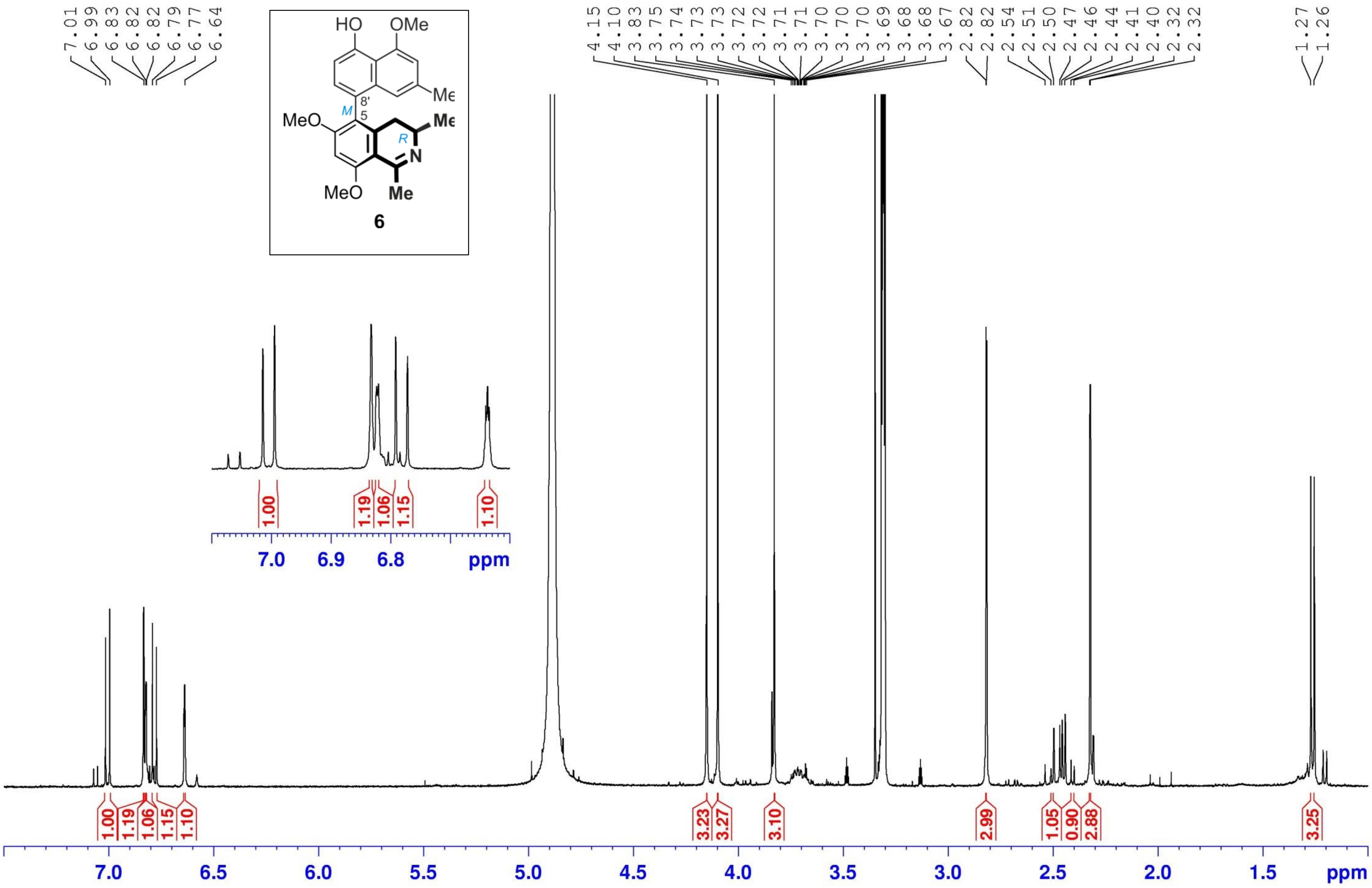

Fig. S57 ^{1}H NMR spectrum of compound **6** in methanol-d_4

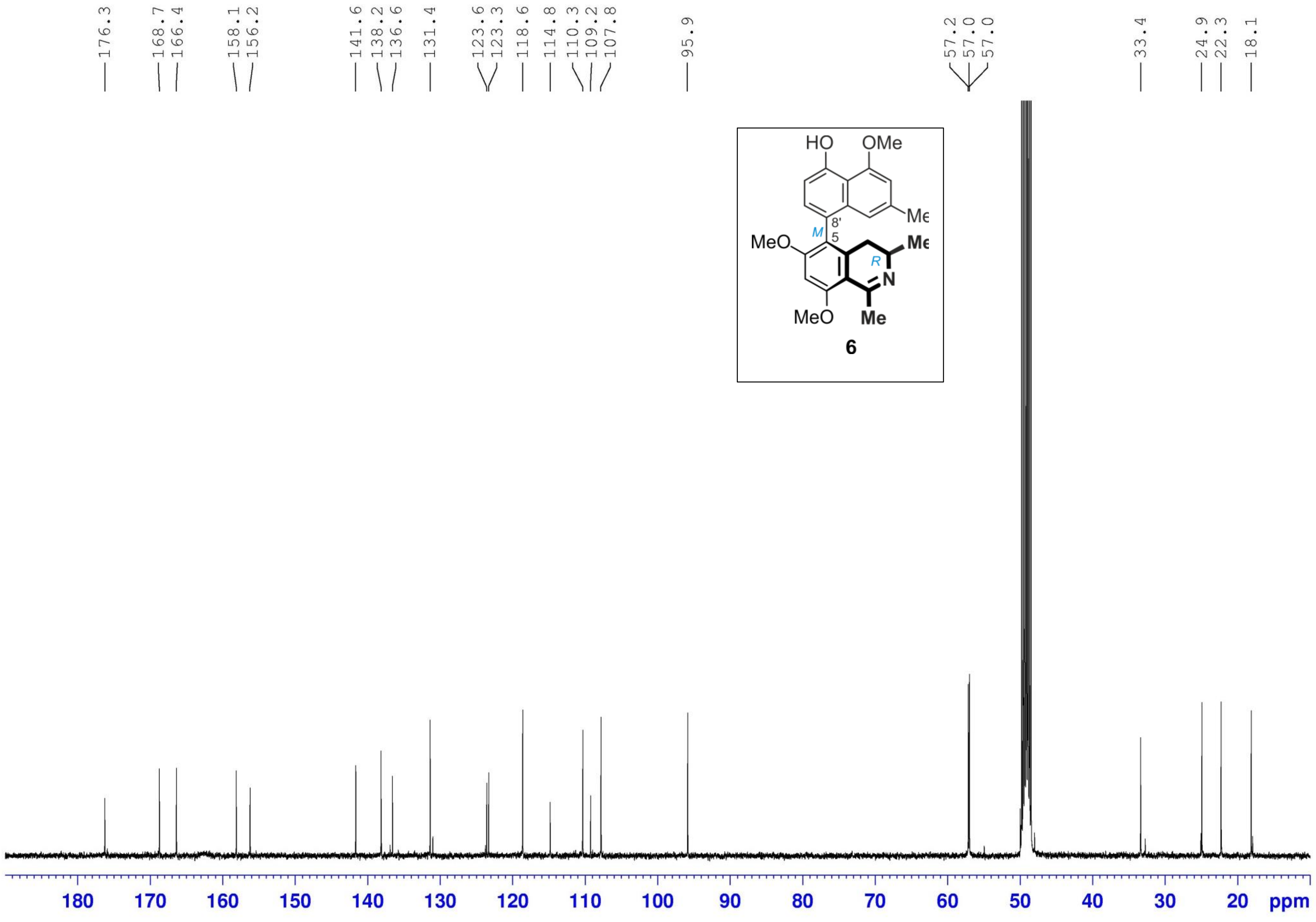

Fig. S58 ^{13}C NMR spectrum of compound **6** in methanol-d_4

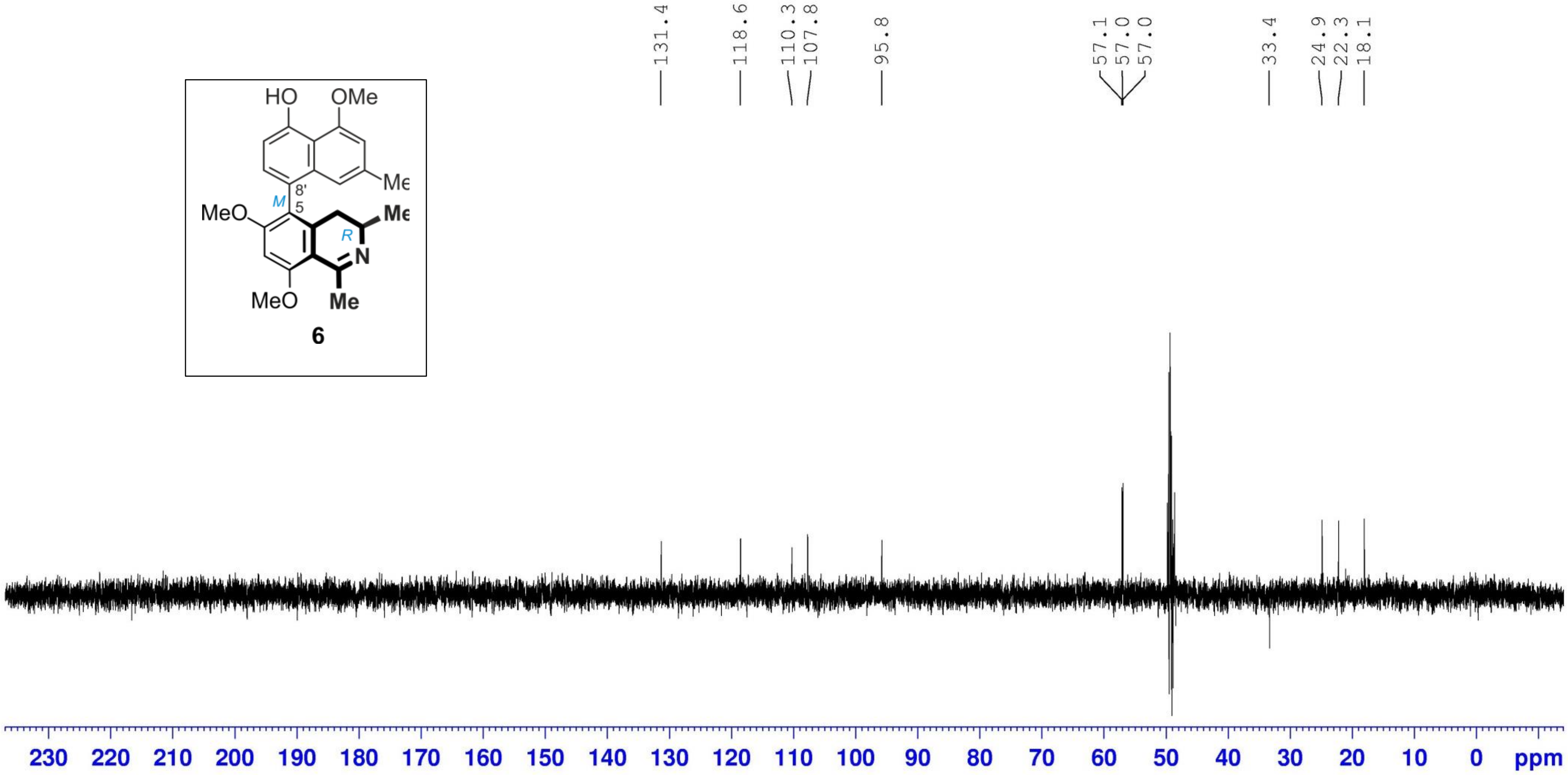

Fig. S59 DEPT-135 NMR spectrum of compound **6** in methanol-d_4

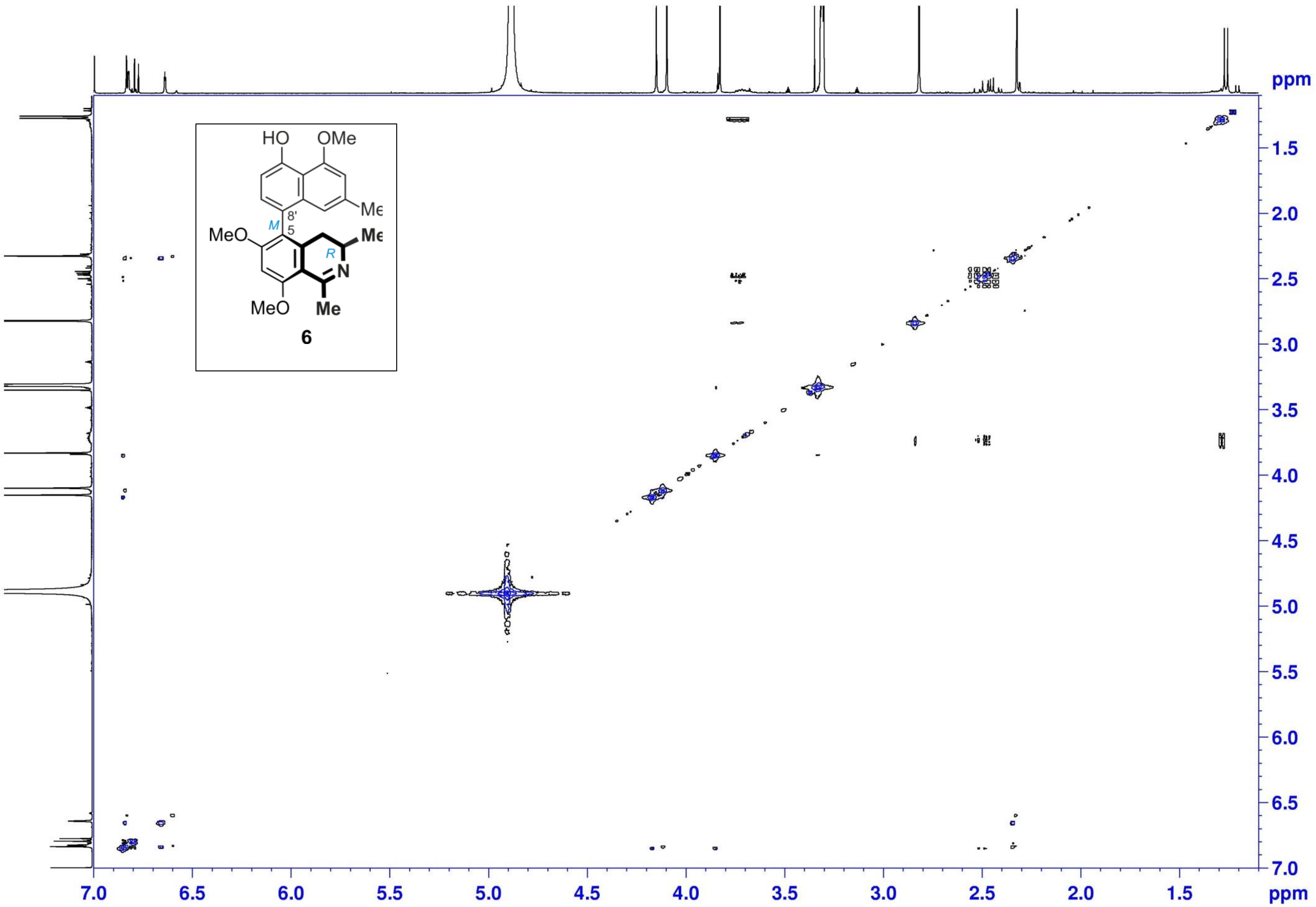

Fig. S60 COSY NMR spectrum of compound **6** in methanol-d_4

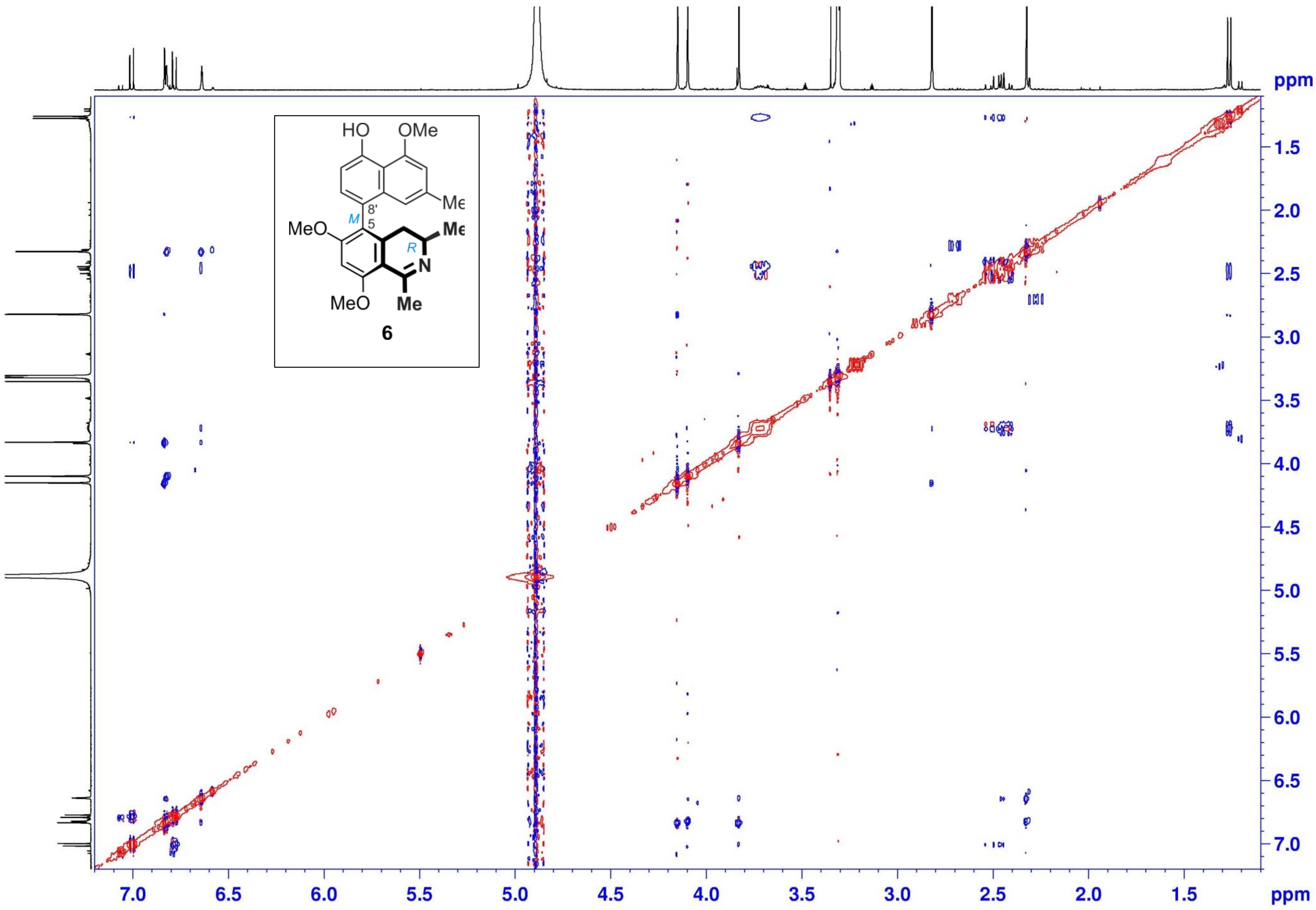

Fig. S61 NOESY NMR spectrum of compound **6** in methanol-d_4

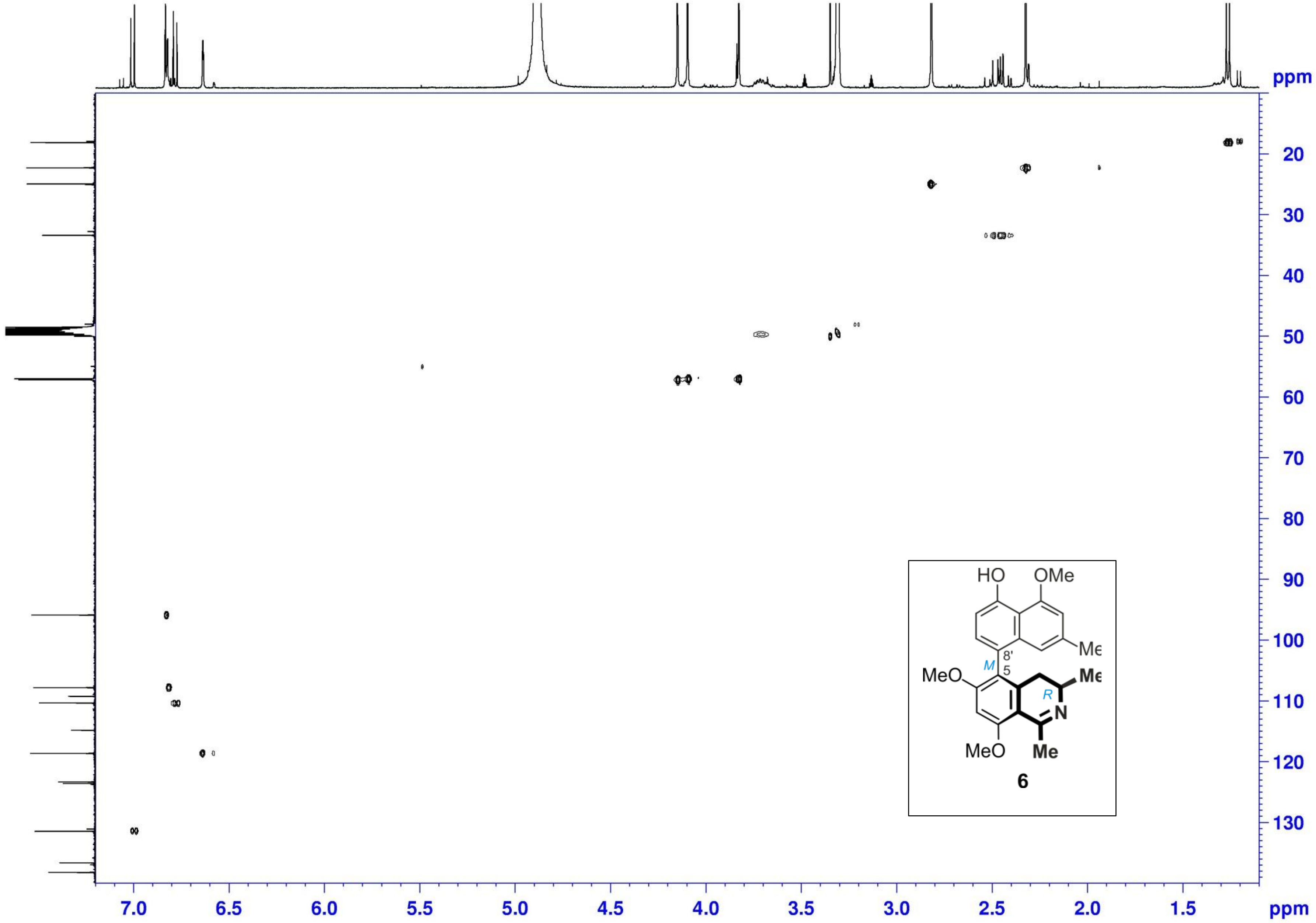

Fig. S62 HSQC NMR spectrum of compound **6** in methanol-d_4

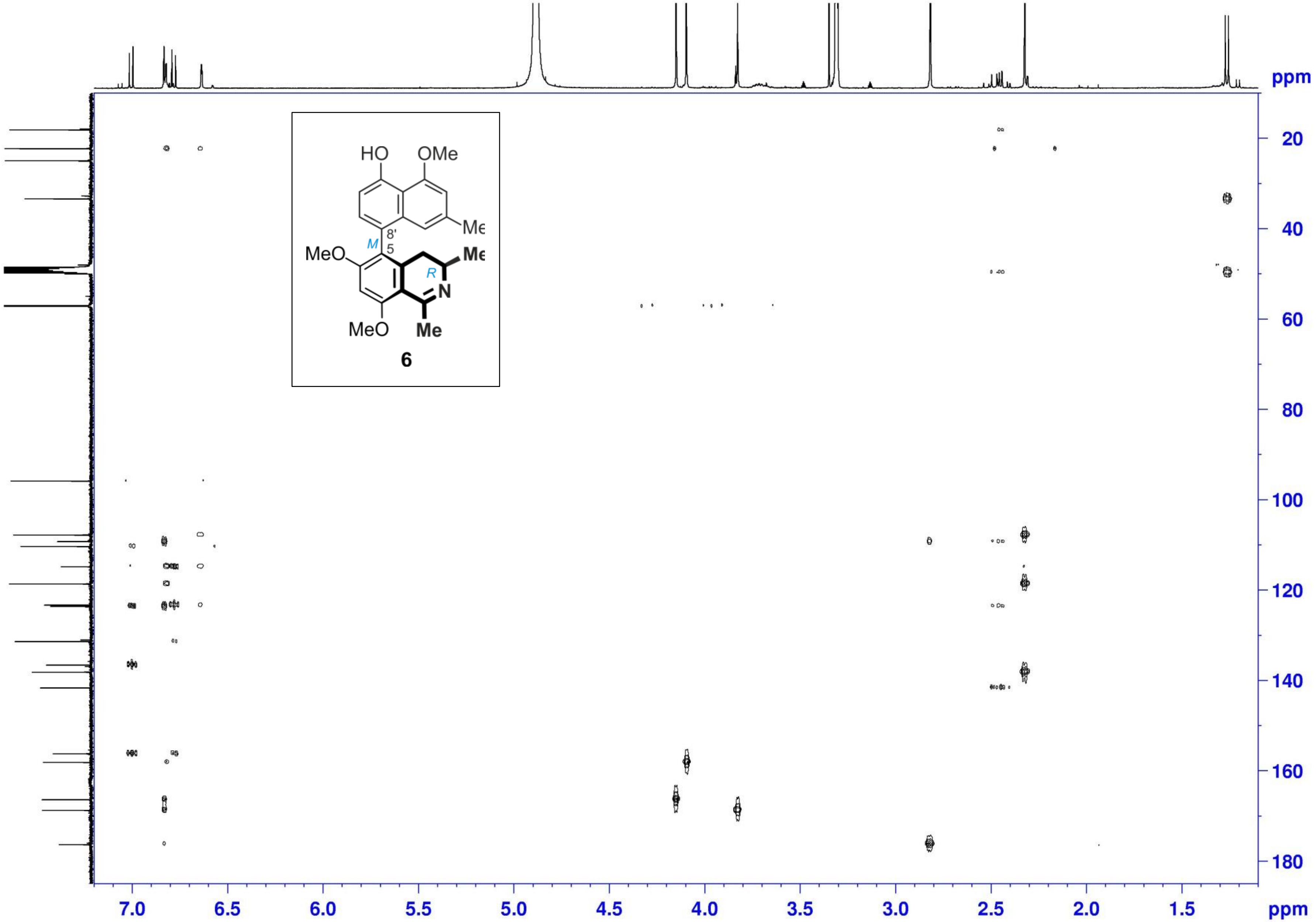

Fig. S63 HMBC NMR spectrum of compound **6** in methanol-d_4

Mass Spectrum SmartFormula Report

Analysis Info

Analysis Name	D:\Data\Spektren2017\2017_3187_BRI_BO3-DCM-2_28_01_2709.d	Acquisition Date	10/18/2017 12:51:23 PM
Method	automation_esi_tune_pos_low_ja_meoh.m	Operator	J.Adelmann
Sample Name	2017_3187_BRI_BO3-DCM-2	Instrument	micrOTOF-Q III 8228888.20516
Comment	Kimbadi Lombe Blaise<br>BO3-DCM-2<br>4pmol/ul in MeOH		

Acquisition Parameter

Source Type	ESI	Ion Polarity	Positive	Set Nebulizer	0.7 Bar
Focus	Not active	Set Funnel 1 RF	100.0 Vpp	Set Dry Heater	200 °C
Scan Begin	50 m/z	Set Funnel 2 RF	200.0 Vpp	Set Dry Gas	5.0 l/min
Scan End	3500 m/z	Set Hexapole RF	300.0 Vpp	Set Divert Valve	Source

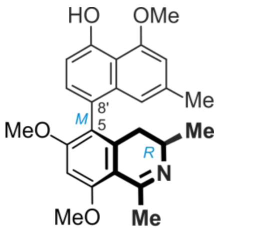

6

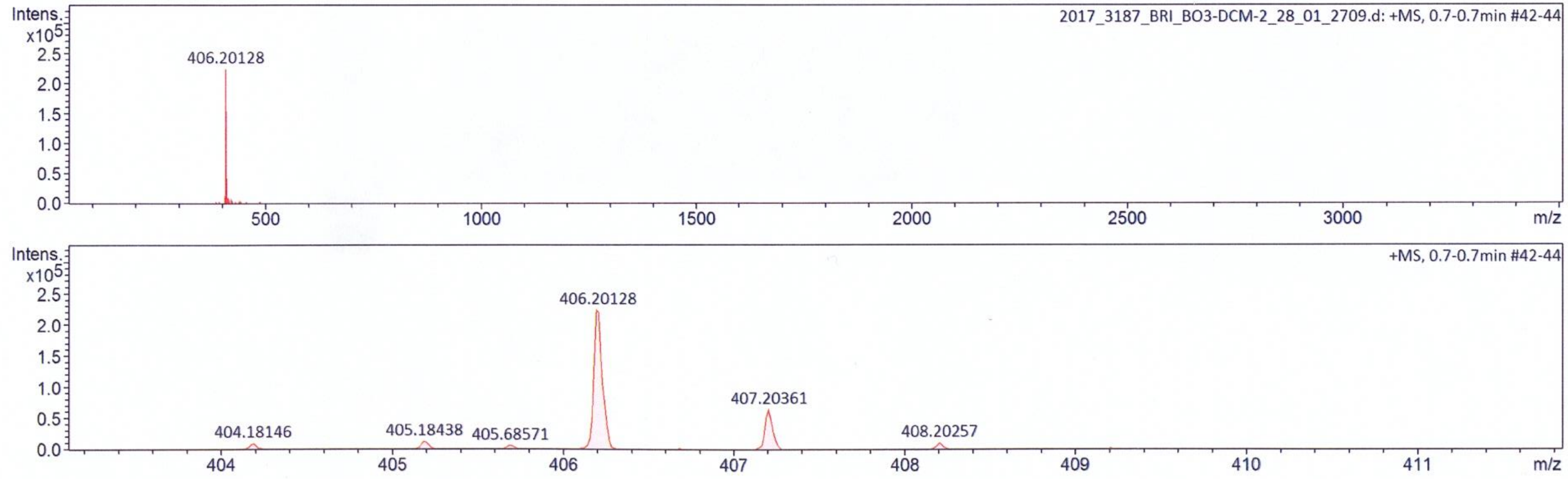

Meas. m/z	#	Ion Formula	m/z	err [ppm]	mSigma	# mSigma	Score	rdb	e⁻ Conf	N-Rule
406.20128	1	C25H28NO4	406.20128	0.01	1.9	1	100.00	12.5	even	ok

Bruker Compass DataAnalysis 4.2 printed: 10/18/2017 2:03:29 PM by: J.Adelmann Page 1 of 1

Fig. S64 HRESIMS spectrum of compound **6**

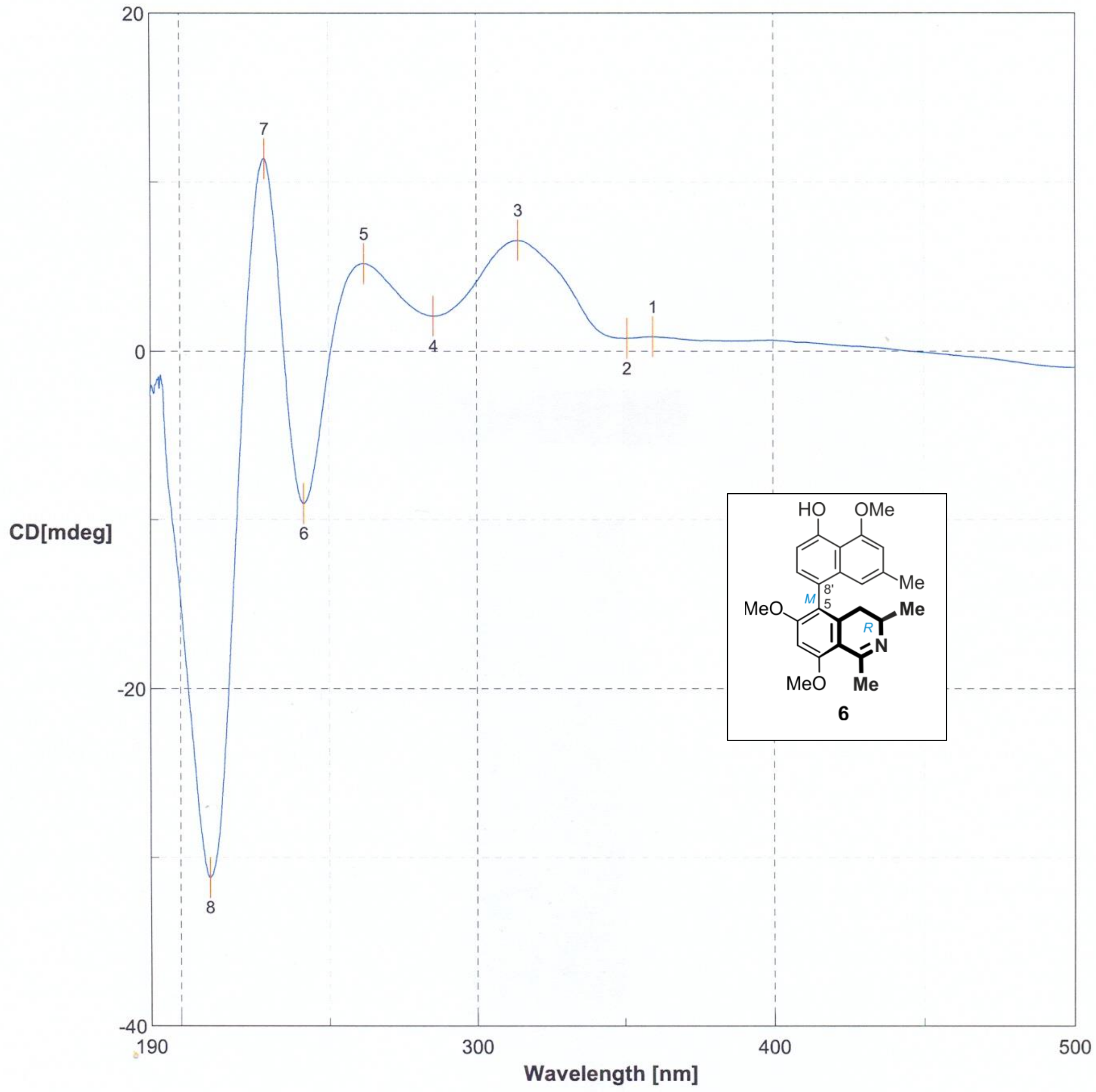

Date/Time	07.12.2015 16:07
Operator	kimbadi
File Name	BO3-DCM-2_0#008.jws
Sample Name	BO3-DCM-2_0.5mm_MeOH
Comment	offline

No.	nm	CD[mdeg]	No.	nm	CD[mdeg]	No.	nm	CD[mdeg]
1	359.8	0.84886	2	351	0.744126	3	314.2	6.54529
4	285.5	2.06942	5	261.9	5.18564	6	241.5	-9.03143
7	228.5	11.3892	8	209.9	-31.1757			

Fig. S65 CD spectrum of compound **6** in methanol

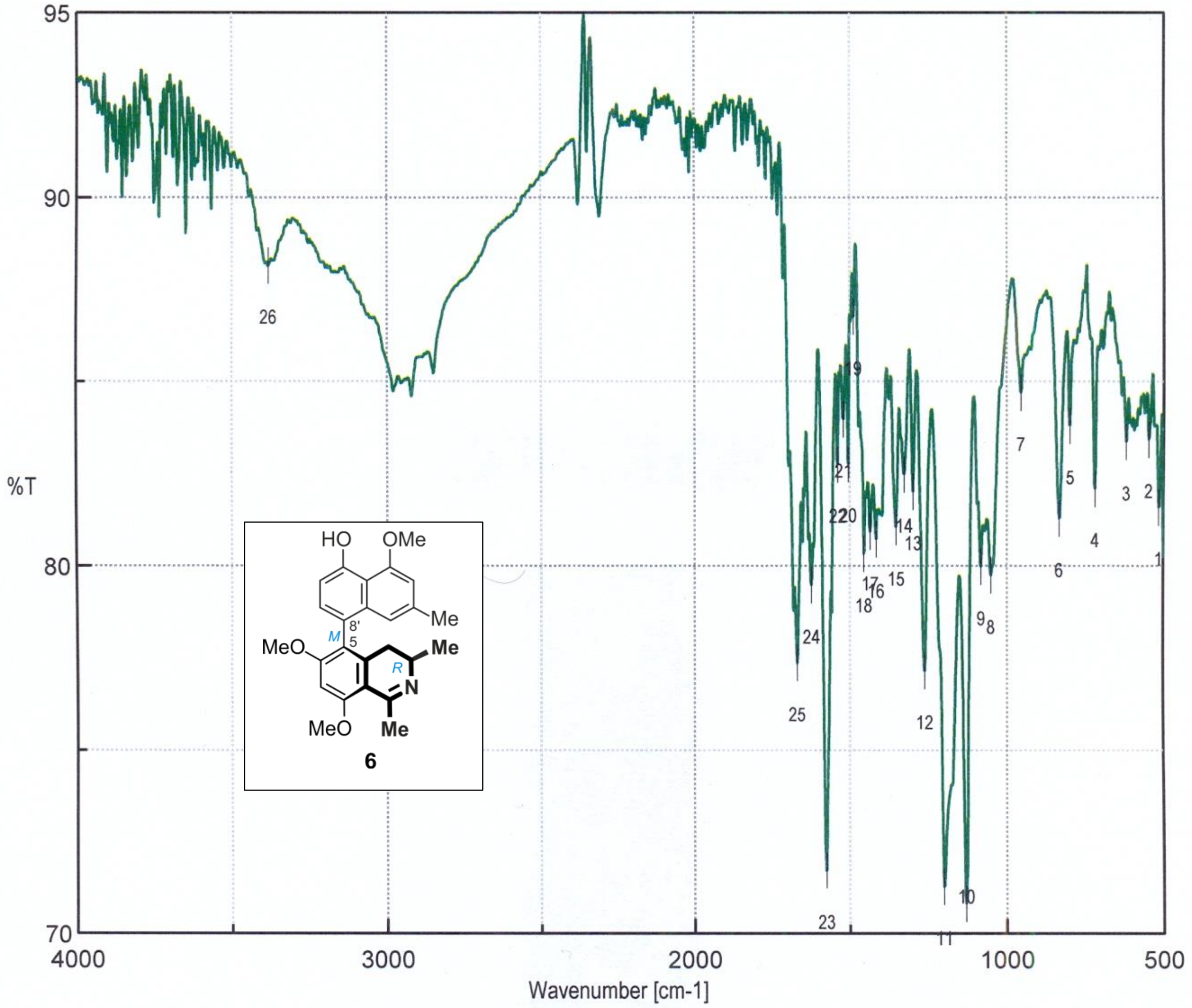

Results of Peak Find

No.	Position	Intensity	No.	Position	Intensity
1	514.901	81.5707	2	546.72	83.4091
3	618.074	83.3565	4	719.318	82.0696
5	798.385	83.7888	6	834.062	81.2748
7	955.555	84.6867	8	1051.98	79.7329
9	1083.8	79.9696	10	1130.08	70.8203
11	1199.51	71.2812	12	1263.15	77.1434
13	1298.82	81.9981	14	1327.75	82.4623
15	1353.78	81.0307	16	1418.39	80.7039
17	1436.71	80.9161	18	1456.96	80.3115
19	1488.78	86.739	20	1507.1	82.757
21	1521.56	83.9655	22	1540.85	82.7297
23	1576.52	71.7095	24	1625.7	79.471
25	1670.05	77.3638	26	3383.5	88.127

[Comments]

Sample name	BO3-DCM-2b
Comment	drop(MeOH)
User	Kimbadi
Division	AK Bringmann
Company	Uni Würzburg

[Measurement Information]

Model Name	FT/IR-4600typeA
Serial Number	D063461786
Accessory	ATR PRO ONE
Accessory S/N	A070661809
Incident angle	45 deg
Measurement Date	20.12.2017 16:02
Light Source	Standard
Detector	TGS
Accumulation	16
Resolution	4 cm-1
Zero Filling	On
Apodization	Cosine
Gain	Auto (4)
Aperture	Auto (7.1 mm)
Scanning Speed	Auto (2 mm/sec)
Filter	Auto (30000 Hz)

Fig. S66 IR spectrum of compound **6**

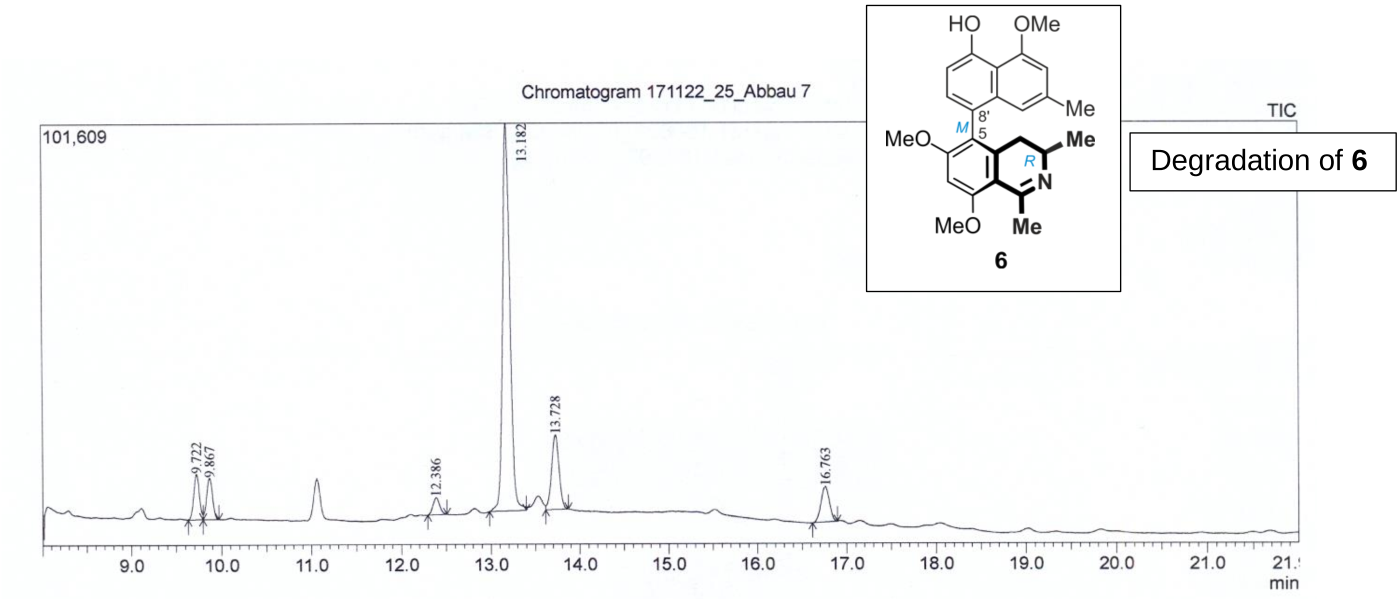

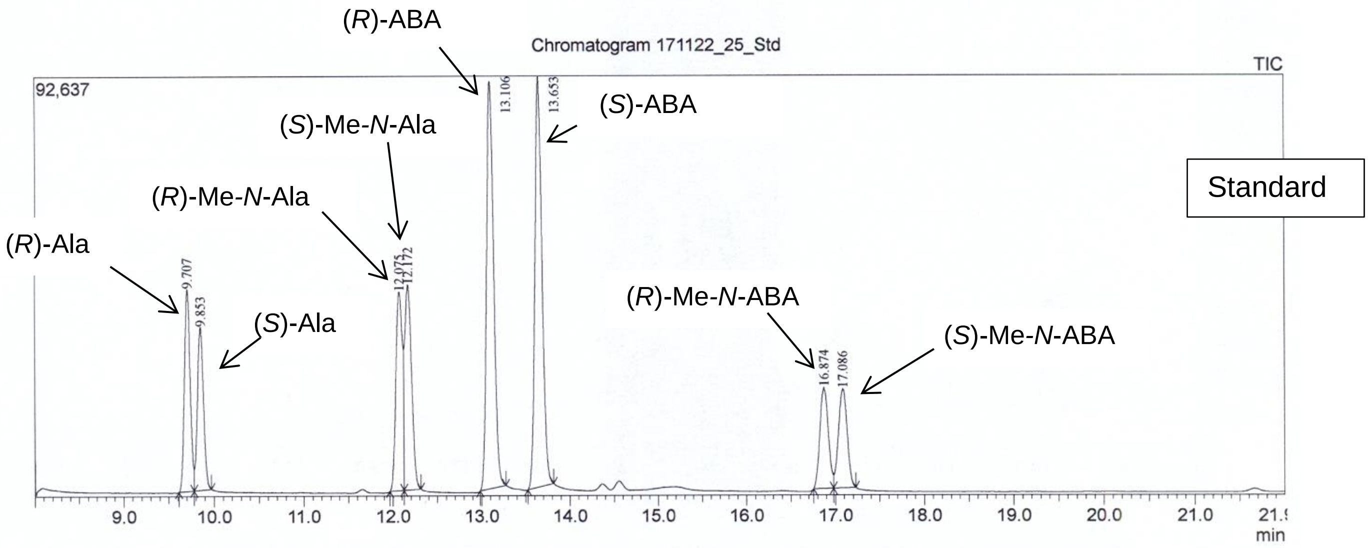

Where: Ala = alanine; Me-*N*-Ala = *N*-*me*thylalanine;
ABA = 3-aminobyturic acid; Me-*N*-ABA = *N*-methyl 3-aminobyturic acid

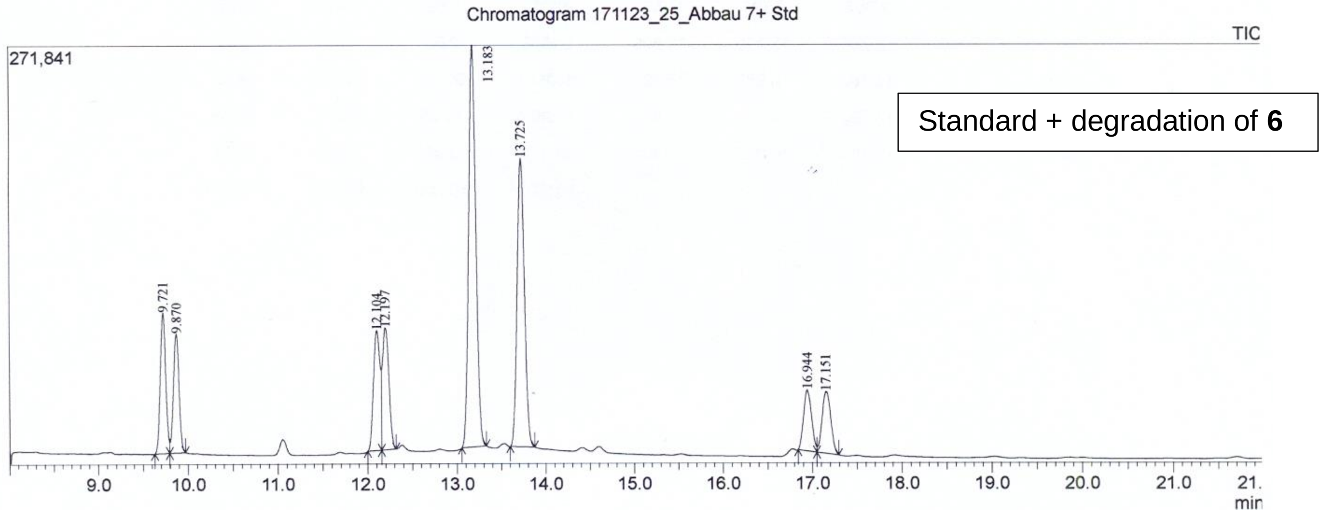

Fig. S67 Results of the oxidative degradation of compound **6**
